# Clonal evolution of acute myeloid leukemia revealed by high-throughput single-cell genomics

**DOI:** 10.1038/s41467-020-19119-8

**Published:** 2020-10-21

**Authors:** Kiyomi Morita, Feng Wang, Katharina Jahn, Tianyuan Hu, Tomoyuki Tanaka, Yuya Sasaki, Jack Kuipers, Sanam Loghavi, Sa A. Wang, Yuanqing Yan, Ken Furudate, Jairo Matthews, Latasha Little, Curtis Gumbs, Jianhua Zhang, Xingzhi Song, Erika Thompson, Keyur P. Patel, Carlos E. Bueso-Ramos, Courtney D. DiNardo, Farhad Ravandi, Elias Jabbour, Michael Andreeff, Jorge Cortes, Kapil Bhalla, Guillermo Garcia-Manero, Hagop Kantarjian, Marina Konopleva, Daisuke Nakada, Nicholas Navin, Niko Beerenwinkel, P. Andrew Futreal, Koichi Takahashi

**Affiliations:** 1 Department of Leukemia, The University of Texas MD Anderson Cancer Center, Houston, TX USA; 2 Department of Hematology and Oncology, Graduate School of Medicine, The University of Tokyo, Tokyo, Japan; 3 Department of Genomic Medicine, The University of Texas MD Anderson Cancer Center, Houston, TX USA; 4 Department of Biosystems Science and Engineering, ETH Zurich, Basel, Switzerland; 5 SIB Swiss Institute of Bioinformatics, Basel, Switzerland; 6 Department of Molecular and Human Genetics, Baylor College of Medicine, Houston, TX USA; 7 Department of Hematopathology, The University of Texas MD Anderson Cancer Center, Houston, TX USA; 8 Department of Neurosurgery, The University of Texas Health Science Center at Houston, Houston, TX USA; 9 Department of Oral and Maxillofacial Surgery, Hirosaki University Graduate School of Medicine, Aomori, Japan; 10 Department of Genetics, The University of Texas MD Anderson Cancer Center, Houston, TX USA; 11 Department of Bioinformatics and Computational Biology, The University of Texas MD Anderson Cancer Center, Houston, TX USA

**Keywords:** Cancer genomics, Acute myeloid leukaemia, Tumour heterogeneity

## Abstract

Clonal diversity is a consequence of cancer cell evolution driven by Darwinian selection. Precise characterization of clonal architecture is essential to understand the evolutionary history of tumor development and its association with treatment resistance. Here, using a single-cell DNA sequencing, we report the clonal architecture and mutational histories of 123 acute myeloid leukemia (AML) patients. The single-cell data reveals cell-level mutation co-occurrence and enables reconstruction of mutational histories characterized by linear and branching patterns of clonal evolution, with the latter including convergent evolution. Through xenotransplantion, we show leukemia initiating capabilities of individual subclones evolving in parallel. Also, by simultaneous single-cell DNA and cell surface protein analysis, we illustrate both genetic and phenotypic evolution in AML. Lastly, single-cell analysis of longitudinal samples reveals underlying evolutionary process of therapeutic resistance. Together, these data unravel clonal diversity and evolution patterns of AML, and highlight their clinical relevance in the era of precision medicine.

## Abstract (web summary)

Understanding the evolutionary trajectory of cancer samples may enable understanding resistance to treatment. Here, the authors used single cell sequencing of a cohort of acute myeloid leukemia tumours and identify features of linear and branching evolution in tumours.

## Introduction

A growing body of evidence supports the role of clonal diversity in therapeutic resistance, recurrence, and poor outcomes in cancer^1^. Clonal diversity also reflects the history of the accumulation of somatic mutations within a tumor. Thus, a precise characterization of clonal diversity reveals not only the extent of a tumor’s clonal complexity but also the evolutionary history of the tumor’s development. Much of the work characterizing the clonal architecture of tumors has been done by computational inference using variant allele fraction (VAF) data from massively parallel DNA sequencing of bulk tumor samples^2–4^. However, the ability to infer clonal heterogeneity and tumor phylogeny from bulk sequencing data is inherently limited, because bulk sequencing techniques cannot reliably infer mutation co-occurrences and hence often fail in accurately reconstructing clonal substructure.

Single-cell DNA sequencing (scDNA-seq) can address some of these challenges^5–9^. However, until recently, the available methods required laborious single-cell isolation protocols and suffered from low cell throughput, limited gene coverage, and technical artifacts from whole-genome amplification that hindered their ability to characterize clonal architecture precisely^10^. Recent technological advances in microfluidics and molecular barcoding now allow rapid single-cell genotyping of targeted cancer-related genes in thousands of cells. We previously described the performance and feasibility of a scDNA-seq platform (Tapestri®, Mission Bio, Inc.) in primary samples from two patients with acute myeloid leukemia (AML)^11^. Here, using this method, we conducted scDNA-seq in 154 AML samples from 123 patients. In 26 of these patients, we have simultaneously profiled DNA and cell surface proteins. Our study uncovered the landscape of AML clonal architecture at single-cell resolution and revealed clonal relationships of AML driver mutations. Using the data, we reconstructed the mutational history of driver genes and demonstrated linear as well as branching clonal evolution patterns in AML. Simultaneous DNA and protein profiling enabled genotype-to-phenotype correlation at single-cell resolution. In addition, scDNA-seq of longitudinal samples in 15 patients allowed illustration of clonal evolution in response to therapeutic selective pressures.

## Results

### The cellular-level landscape of driver mutations in AML

**Fig. 1:**
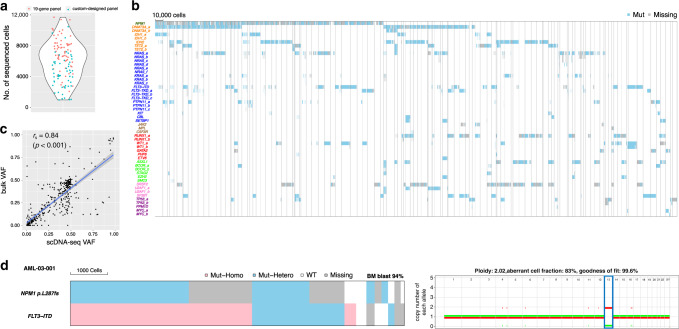
The Genetic landscape of AML based on single-cell DNA sequencing. **a** Distribution of the number of total sequenced cells. Each point represents a sample from unique patients. **b** Somatic mutations in 735,483 cells from 123 AML patients detected by single-cell DNA sequencing (scDNA-seq). Each column represents a cell at the indicated scale, and cells from the same case are clustered together within the areas surrounded by the gray lines. Cells that were genotyped as being mutated or wild type for the indicated gene are colored in blue and white, respectively. Cells with missing genotypes are colored in gray. When one sample has multiple different mutations in the same gene, they were annotated differently (e.g., *DNMT3A*_a and *DNMT3A*_b). Mutated genes are colored based on the affected molecular pathway (nucleophosmin colored in green, DNA methylation in orange, RTK/RAS/MAP kinase pathway in blue, JAK-STAT pathway in brown, transcription factor in red, chromatin/cohesin in light green, splicing in pink, and apoptosis in purple). A total of 76,549 cells that were genotyped as wild type for all the variants screened are not shown. **c** Correlation of the variant allele fraction (VAF) from bulk-sequencing and scDNA-seq. The *x*-axis shows the VAF from scDNA-seq (scDNA-seq VAF). The *y*-axis shows the VAF from the bulk sequencing (bulk VAF). Each dot represents a detected variant. The line represents a linear regression line. The shaded area represents the 95% confidence intervals. **d** A representative case with highly homozygous variant involving copy-neutral loss of heterozygosity (CN-LOH). Heat map (left) shows the genotype of each sequenced cell for each variant, with clustering based on the genotypes of driver mutations. Each column represents a cell at the indicated scale. Cells with homozygous mutation, heterozygous mutation, and wild-type cells are indicated in red, blue, and white, respectively. Cells with missing genotypes are indicated in gray. The allele counts distribution is shown to the right. The allele count is shown on the vertical axis, and the chromosomes are shown on the horizontal axis. Chromosome 13 involving highly homozygous *FLT3*-ITD is highlighted with a blue rectangle. Mut-Homo homozygously mutated, Mut-Hetero heterozygously mutated, WT wild type, Missing missing genotype.

We analyzed 154 samples of bone marrow mononuclear cells (BMMCs) from 123 AML patients, of which 88 (72%) were previously untreated, and 35 (28%) had relapsed or refractory disease (clinical characteristics are summarized in Table 1). The median bone marrow blasts percentage was 46% (interquartile range [IQR]: 30–67%). All samples were concurrently analyzed by conventional bulk next-generation sequencing (bulk-seq) and scDNA-seq. Based on the mutation profiles of the samples determined by the conventional bulk-seq, scDNA-seq was conducted by one of the two targeted panels (Mission Bio’s predesigned 19 genes panel [90 samples, Supplementary Table 1] or by a custom-designed panel interrogating 37 genes with recurrent mutations in cancer [64 samples, Supplementary Data 2], Supplementary Methods). A median of 6102 BMMCs (IQR: 4066–7790) per sample were sequenced by the scDNA-seq platform (Fig. 1a). scDNA-seq resulted in a median of 48× coverage per amplicon per cell (IQR: 23×–87×, Supplementary Fig. 1). The amplicons covering guanine–cytosine (GC)-rich sequences, such as *GATA2*, *SRSF2*, and parts of *RUNX1* and *TP53*, had lower coverage compared with other regions, such that relatively large numbers of cells had inconclusive genotype information for the mutations covered by these amplicons (Supplementary Fig. 2). The estimated median allele dropout (ADO) rate was 5.8% (IQR: 4.8–7.0%) (Supplementary Fig. 3). The estimated lower limit of detection of the platform was 0.1% of the cellular population based on the serial dilution assay of a cell line and also from mutation validation by droplet digital polymerase chain reaction (PCR) (Supplementary Table 2 and Supplementary Fig. 4).

**Table 1:** Clinical and demographic characteristics of the study cohort (*N* = 123).

Characteristics	Median	IQR
WBC (×10^3^/L)	8	3.5–30.0
HGB (g/dL)	9.1	8.5–10.0
PLT (×10^3^/μL)	55	29–86
BM blasts (%)	46	30–67
PB blasts (%)	27	5–57
LDH (U/L)	689	478–1154
Age (y)	61	52–73
	No.	%
*Ontogeny*		
De novo	93	76
Secondary/therapy related	30	24
*Prior treatment*		
Untreated	88	72
Treated	35	28
*Karyotype*		
Normal karyotype	91	74
Complex karyotype	12	10
Others	20	16
*Treatment*		
IA-based chemotherapy	49	40
AraC-based chemotherapy	12	10
decitabine and venetoclax	27	22
HMA without venetoclax	24	20
Others	11	9
*Sex*		
Female	48	39

Abbreviations: *IQR* interquartile range, *WBC* white blood cells, *HGB* hemoglobin, *PLT* platelets, *BM* bone marrow, *PB* peripheral blood, *LDH* lactate dehydrogenase, *AML* acute myeloid leukemia, *IA* idarubicin and cytarabine, *AraC* cytarabine, *HMA* hypomethylating agents.

In total, we sequenced 735,483 BMMCs from 123 AML patient samples (Fig. 1b). The scDNA-seq approach detected 543 somatic mutations in 31 cancer-associated genes, which included 388 (71%) single-nucleotide variants and 155 (29%) small insertion–deletions (indels). Among these, 530 mutations (98%) were orthogonally validated: 489 (92%) by conventional bulk-seq^12^ (median 397×), 29 (5%) by droplet digital PCR (ddPCR), and 12 (2%) by a quantitative PCR assay (all *FLT3*-internal tandem duplication [ITD]). Of the 13 unvalidated mutations, 3 were negative by ddPCR, and the remaining 10 were not tested due to the lack of remaining specimens. The subsequent analyses used a final set of 530 validated mutations (Supplementary Data 1). Of note, among the shared genomic regions covered by the scDNA-seq and the bulk-seq platforms, all mutations called by the bulk-seq were also detected by scDNA-seq. The VAF from bulk-seq (bulk VAF) and the VAF inferred from the scDNA-seq data (scDNA-seq VAF) were in good concordance (*r*_s_ = 0.84, *p* < 0.001) suggesting that the sequenced cells are representative of the total bulk samples (Fig. 1c and Supplementary Fig. 5).

The most frequently detected mutations by scDNA-seq in the 123 patients were in *NPM1* (*N* = 49, 40%), followed by *FLT3* (*N* = 47, 38%; 36 [29%] with ITD and 22 [18%] with non-ITD mutations), *DNMT3A* (*N* = 45, 37%), *NRAS* (*N* = 45, 37%), *IDH2* (*N* = 33, 27%), *RUNX1* (*N* = 25, 20%), *SRSF2* (*N* = 25, 20%), *TET2* (*N* = 20, 16%), and *KRAS* (*N* = 19, 15%). scDNA-seq detected substantially more *FLT3* mutations (12 [80%] ITD and 3 [20%] non-ITD) than bulk-seq (Supplementary Fig. 6a). This is likely due to the capability of the scDNA-seq platform in detecting cryptic *FLT3* mutations in small cellular subpopulations (Supplementary Fig. 6b), which has been reported previously for a different single-cell technology^13^.

scDNA-seq calls mutations in individual cells with zygosity state, which allows to observe additional layer of diversity. However, the lack of the validation method in previous studies has made the interpretation of zygosity difficult^5^. In the current cohort, we sought to validate the zygosity state by concurrently performing single-nucleotide polymorphism (SNP) arrays in selected samples. We detected copy-neutral loss of heterozygosity (CN-LOH) in samples with cells having homozygous *FLT3*-ITD, *RUNX1*, and *TET2* mutations (Fig. 1d and Supplementary Fig. 7a, b), confirming that the observation of homozygously mutated cells in these samples was likely true and was as a result of CN-LOH. In contrast, none of the samples with cells homozygous for *SRSF2* or *NPM1* (Supplementary Fig. 7b–d) mutations had copy number alterations involving the mutated loci. These results do not rule out the possibility that SNP arrays failed to detect the subclonal allelic imbalance. However, the cells that were genotyped as homozygous had significantly lower sequencing depth than did the cells that were genotyped as heterozygous (Supplementary Fig. 7), suggesting that the homozygous calls in these mutations may have resulted from low sequencing depth and ADO. These results indicate that concurrent copy number analysis is necessary for the accurate interpretation of the zygosity data from scDNA-seq.

### Clonal relationships of AML driver mutations

**Fig. 2:**
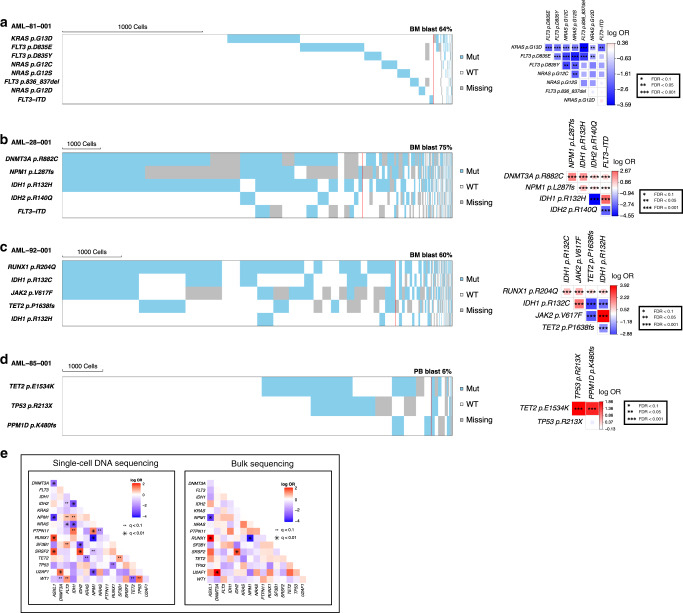
The cellular-level mutual exclusivity of AML driver mutations. **a**–**d** Cell-level mutual exclusivity patterns of driver mutations in individual samples for four representative cases. **a** *KRAS*, *NRAS*, *FLT3*-non-ITD, and *FLT3*-ITD, **b** *IDH1* and *IDH2*, **c** *IDH1* p.R132C, *IDH1* p.R132H, and *TET2*, **d** *TP53* and *PPM1D* variants did not co-occur in the same cellular populations. Mut mutated, WT wild type, Missing missing genotype. Heat maps (left) show the genotype of each sequenced cell for each variant, with clustering based on the genotypes of driver mutations. Each column represents a cell at the indicated scale. Cells with mutations and wild-type cells are indicated in blue and white, respectively. Cells with missing genotypes are indicated in gray. The subclones located to the right of the red line comprised <1% of the total sequenced cells, and such small subclones can represent false positive or negative genotypes as a result of allele dropout or multiplets. The figures on the right show the pairwise association of mutations. The color and size of each panel represent the degree of the logarithmic odds ratio (log OR). The bar on the right side is a key indicating the association of the colors with the log OR. Co-occurrence and mutual exclusivity are indicated by red and blue, respectively. The statistical significance of the associations based on the false discovery rate (FDR) is indicated by the asterisks (*FDR < 0.1, **FDR < 0.05, ***FDR < 0.001). **e** Pairwise association of driver mutations in AML based on single-cell DNA sequencing (left) and bulk sequencing data (right). For each pair of mutations, their dependency was summarized as log OR, with positive values (red) indicating a degree of co-occurrence and negative values (blue) indicating a degree of mutual exclusivity. The statistical significance of the associations based on the *q* value is indicated by the dots and asterisks (^**^q < 0.1, *q < 0.01).

Single-cell mutation data unambiguously revealed the cellular-level co-occurrence and mutual exclusivity among driver mutations. Multiple different mutations (often subclonal) involving receptor tyrosine kinase (RTK)/Ras GTPase (RAS)/MAP Kinase (MAPK) signaling pathway genes (*FLT3*, *NRAS*, *KRAS*, *PTPN11*, *KIT*, and *MYC*) were detected in the same patients, and they were often present in mutually exclusive clones at the cellular level (Fig. 2a and Supplementary Fig. 8a). A similar mutually exclusive relationship was observed among other functionally redundant mutations (e.g., *IDH1* and *IDH2*; *TET2* and *IDH*, Fig. 2b, c and Supplementary Fig. 8b)*. TP53* and *PPM1D* mutations were also found to be mutually exclusive by scDNA-seq (Fig. 2d). This is in contrast to the findings from previous bulk-seq studies that showed significant co-occurrence of the two mutations at the population level^14,15^. However, because of their functional redundancy in DNA damage response pathway, the true co-occurrence (i.e., cellular-level co-occurrence) between the two mutations has been debated. The result from the scDNA-seq is biologically more consistent with the functional redundancy of the two mutations. *DNMT3A*, *WT1*, and *TET2* were often found to carry two different mutations co-occurring in the same cells, which is consistent with the previously reported biallelic involvement of these tumor suppressor genes (Supplementary Fig. 8c)^16–18^. Pair-wise analysis of mutation co-occurrence using pooled single-cell data identified more significant co-occurrence and mutually exclusive relationships among AML driver genes compared to the same analysis using bulk-seq data from the same samples (Fig. 2e). Taken together, these single-cell genotype data provide cell-level evidence of mutation co-occurrence, which not only validates previous findings by bulk-sequencing studies but also corrects previously mischaracterized relationships (e.g., *TP53* and *PPM1D*).

### Reconstructing evolutionary histories and mutation order

**Fig. 3:**
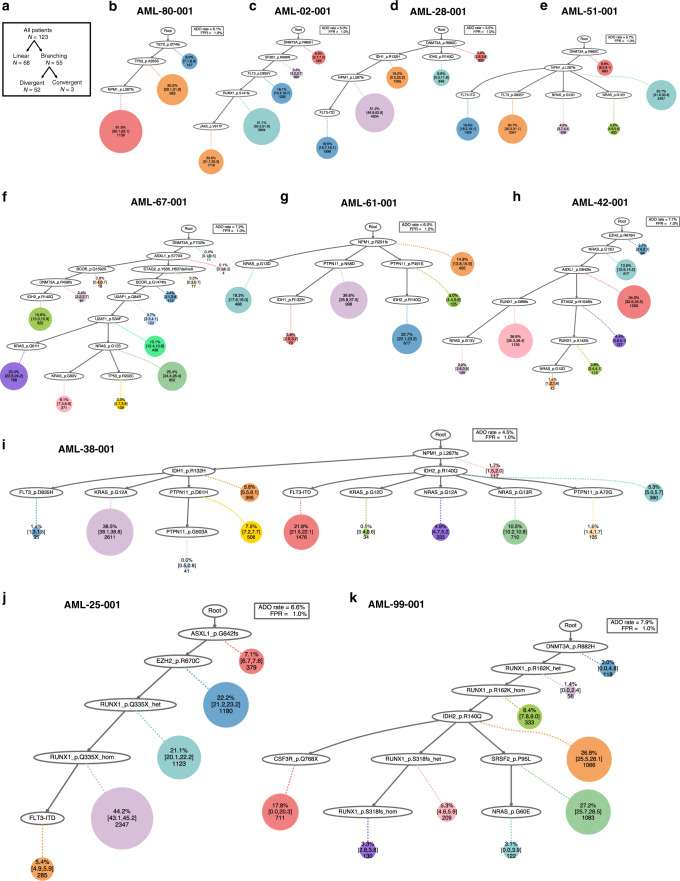
Inference of mutational history in AML. **a** Summary of the clonal evolution patterns. Three of the 55 cases showing branching evolution patterns presented convergent evolution patterns. **b**–**i** Inference of mutation phylogeny based on the single-cell DNA seqeuncing (scDNA-seq) data using the SCITE algorithm. Representative cases illustrating distinct patterns of clonal evolution are shown. Each node represents a mutational event, and each circle represents a subclone with cumulative mutational events, which can be traced with a dotted line and solid lines towards the root. The size of the circle is proportional to the clonal population, and the numbers within each circle are the number of cells and the percentage of each clone among the total tumor cells. The 95% credible intervals from the posterior sampling are shown to illustrate the uncertainty in the subclone sizes. The wild-type cells which did not carry any driver mutations are not shown. **b**, **c** Linear clonal evolution pattern, in which a subset of cells from the founder clone acquired additional mutations in a stepwise manner. The trunk clone exhibits a forked evolution pattern based on the status of additional mutations. **d**–**i** Branching clonal evolution pattern including convergent evolution patterns with molecular alterations in **g** *NPM1*-*RAS/MAPK-IDH*, **h** chromatin-*RUNX1-RAS*, and **i** *NPM1-IDH-FLT3/RAS/MAPK* pathways. The clonal evolution patterns are characterized by the parallel acquisition of multiple functionally redundant mutations in different cell populations. **j**, **k** Inference of the relative timing of loss of heterozygosity (LOH). Zygosity state based on the scDNA-seq data was incorporated into phylogeny reconstruction. Two representative cases with homozygous *RUNX1* mutations involving LOH are shown. In both cases, each *RUNX1* mutation was initially heterozygous and sequentially developed into homozygous state, without acquiring any additional mutations during LOH events. ADO allele dropout, FPR false-positive rate.

To reconstruct evolutionary histories in individual AML, we used single cell inference of tumor evolution (SCITE), a probabilistic model to infer phylogenetic trees from single-cell mutation data that involves a flexible Markov-chain Monte Carlo (MCMC) learning algorithm^19^. SCITE-based phylogenies demonstrated the sequence of mutation acquisition and distinct patterns of clonal evolution in AML including linear and branching models of evolution (Fig. 3a–i). Among the 123 AML patients analyzed, 68 (55%) showed linear clonal evolution, whereas 55 (45%) exhibited branching clonal evolution (Fig. 3a and Supplementary Fig. 9a). As expected, samples with branching evolution showed significantly higher clonal diversity compared to those with linear evolution (median Shannon index 1.83 [IQR: 1.45–2.22] vs. 1.35 [IQR: 1.00–1.57], *p* < 0.001), whereas there were no significant differences in other clinical characteristics between the two models (Supplementary Fig. 9b). When we correlated the clonal diversity index with clinical characteristics, we found a modest positive correlation between patient age and clonal diversity (*r*_s_ = 0.21, *p* = 0.02, Supplementary Fig. 9c). In three cases with branching patterns, we found an evolutionary history that is consistent with convergent evolution (Fig. 3g–i)^20^. For example, in AML-38-001, a putative founding mutation, *NPM1* p.L287fs, diverged into two independent branches with mutations *IDH1* p.R132H and *IDH2* p.R140Q, respectively. Each of these branches then separated into *FLT3* p.D835H, *KRAS* p.G12A, or *PTPN11* p.D61H mutated clones and *FLT3*-ITD, *KRAS* p.G12D, *NRAS* p.G12A, *NRAS* p.G13R, or *PTPN11* p.A72G mutated clones, respectively. As a result, the sample harbored 12 individual clones, each with a combination of functionally similar, but separately evolved, molecular alterations (*NPM1-IDH-RAS/RTK/MAPK* signaling pathway alteration, Fig. 3i). By contrast, bulk-seq data from the same cohort of patients was not able to provide a definitive model of clonal evolution with the same resolution (Supplementary Fig. 10a–d).

While single-cell data provides more definitive models of phylogeny and clonal architecture compared to bulk-seq data, variability in sequencing coverage among cells and amplicons generates uncertainties. For example, poor sequencing coverage in *SRSF2* and other GC-rich amplicons resulted in relatively large numbers of cells with inconclusive genotype (Supplementary Fig. 2), which can lead to inaccurate inference of mutation order and phylogeny. In such cases, we integrated bulk-seq data and scDNA-seq data into consensus phylogenies using the B-SCITE algorithm^21^, suggesting complementarity between the two platforms (Supplementary Fig. 10e). We also incorporated the zygosity state into phylogeny modeling, which revealed the relative timing of LOH events during clonal evolution (Fig. 3j, k).

### Clonal diversity in AML leukemia initiating cells (LICs)

**Fig. 4:**
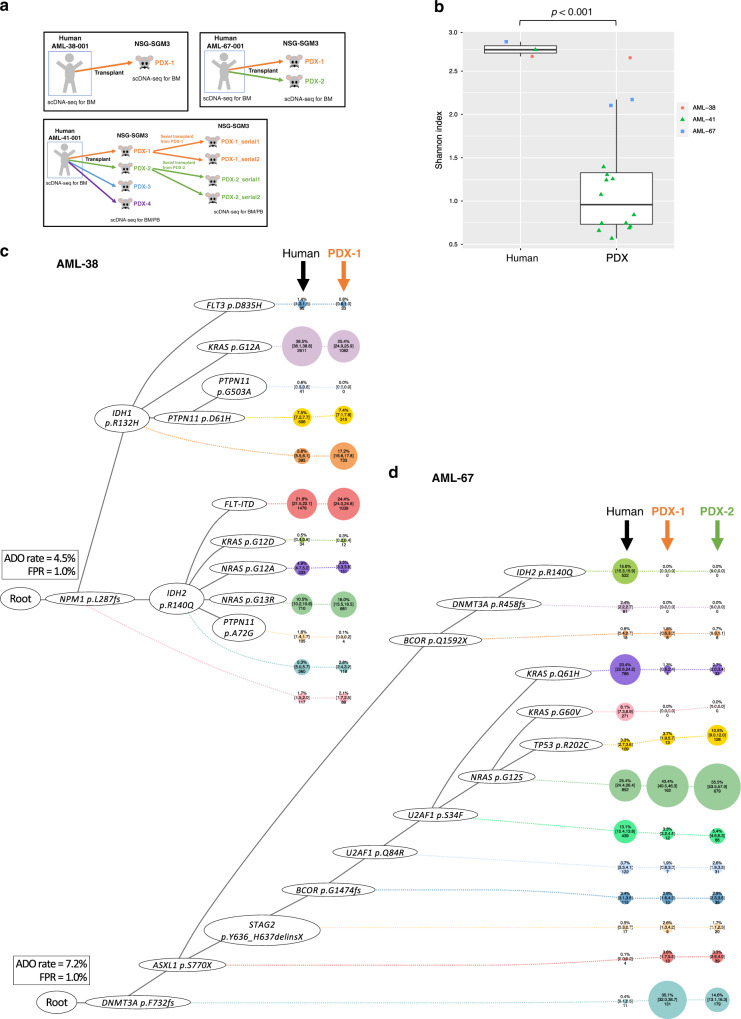
Clonal architecture in xenotransplanted models. NSG-SGM3 mice engrafted with aliquotes of AML-38-001, AML-67-001, and AML-41-001 were analyzed by single-cell DNA sequencing (scDNA-seq). **a** Schematic figures of xenotransplant assay. PDX patient derived xenograft, BM bone marrow, PB peripheral blood. **b** Change in clonal diversity between human and xenotransplanted models. The types of samples are shown on the *x*-axis. The *y*-axis shows Shannon index. The thick line within each box represents the median, and the top and bottom edges of the box represent the 25th and 75th percentiles, respectively. The upper and lower whiskers represent the 75th percentile plus 1.5 times the interquartile range and the 25th percentile minus 1.5 times the interquartile range, respectively. Two-sided Student’s *t* test was used without adjustment for multiple comparisons (*p* = 0.000557). *N* = 19 samples from 3 cases. All data points are shown colored by the donors. PDX patient derived xenograft. **c**, **d** Clonal structure based on scDNA-seq data in human and xenotransplanted samples in **c** AML-38 and **d** AML-67. The phylogenetic trees visualize the estimated order of mutation acquisition and the proportion of subclones with a different combination of mutations at each timepoint. The wild-type cells which did not carry any driver mutations are not shown. ADO allele dropout, FPR false-positive rate.

We then studied clonal diversity and architecture of AML leukemia initiating cells (LICs), as defined by their ability to initiate (or regenerate) AML in immunocompromised mice, at single-cell resolution. We xenotransplanted aliquots of three AML samples with highly branching clonal structure (AML38-001, AML-41-001, and AML67-001) into immunodeficient mice (PDX: patient derived xenograft) and analyzed engrafted human CD45^+^ cells using scDNA-seq (Fig. 4a and Supplementary Fig. 11a). While regenerated AML had contracted diversity compared to the original bulk AML samples (Fig. 4b), it consisted of substantially diverse genetic populations (Fig. 4c, d and Supplementary Fig. 11b). For example, in AML-38-001, which exhibited convergent evolution of multiple AML subclones, 11 of 12 subclones were detected in the engrafted sample (Fig. 4c). In addition, AML subclones showed variable leukemia regenerating capacity, which might reflect the fitness landscape of AML subclones (Fig. 4d and Supplementary Fig. 11b). For instance, the original AML sample from AML-67-001 showed two subclones carrying combinations of *DNMT3A*-*ASXL1*-*STAG2*-*BCOR*-*U2AF1* p.Q84R-*U2AF1* p.S34F with *NRAS* p.G12S or *KRAS* p.Q61H, that were similar in clonal size. Two PDX models were generated from this sample (AML-67-001-PDX1 and AML-67-001-PDX2), and in both models, we detected clonal expansion of the *NRAS* p.G12S clone and regression of the *KRAS* p.Q61H clone (Fig. 4d). Intriguingly, similar clonal dynamics were observed in the actual patient after therapy, suggesting that clonal expansion in PDX models may reflect the functional fitness of the subclones (Supplementary Fig. 12). These data suggest that scDNA-seq of PDX models can reconstruct the heterogeneity of LIC populations and their functional fitness.

### Single-cell mapping of genetic-phenotypic evolution in AML

**Fig. 5:**
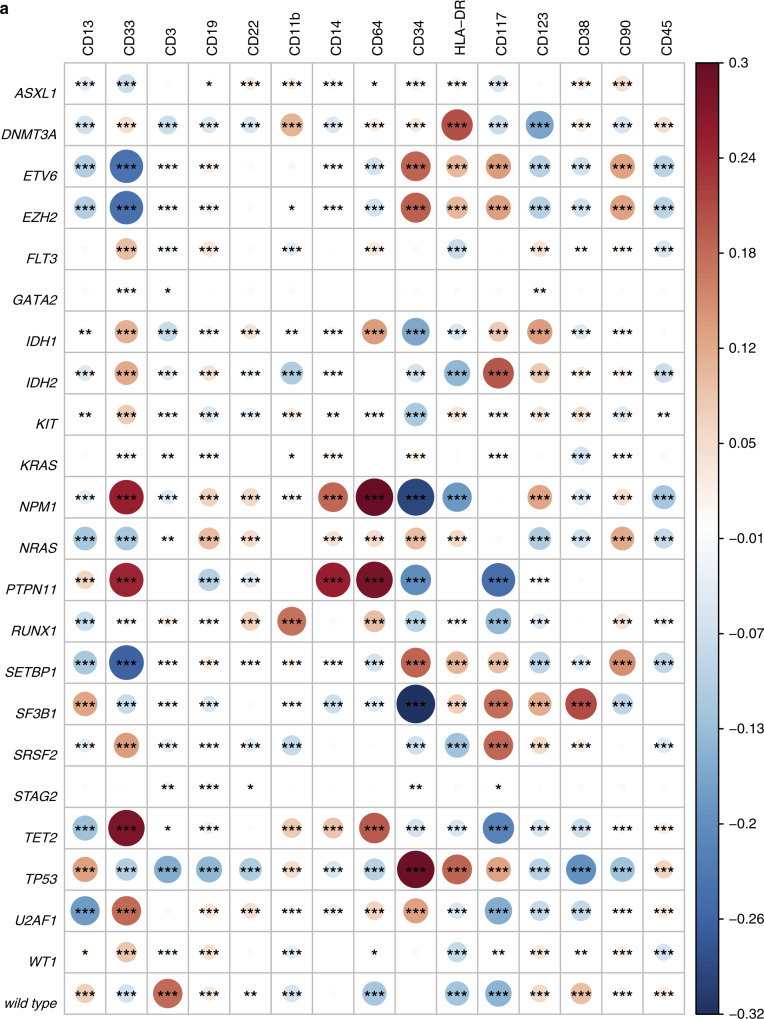

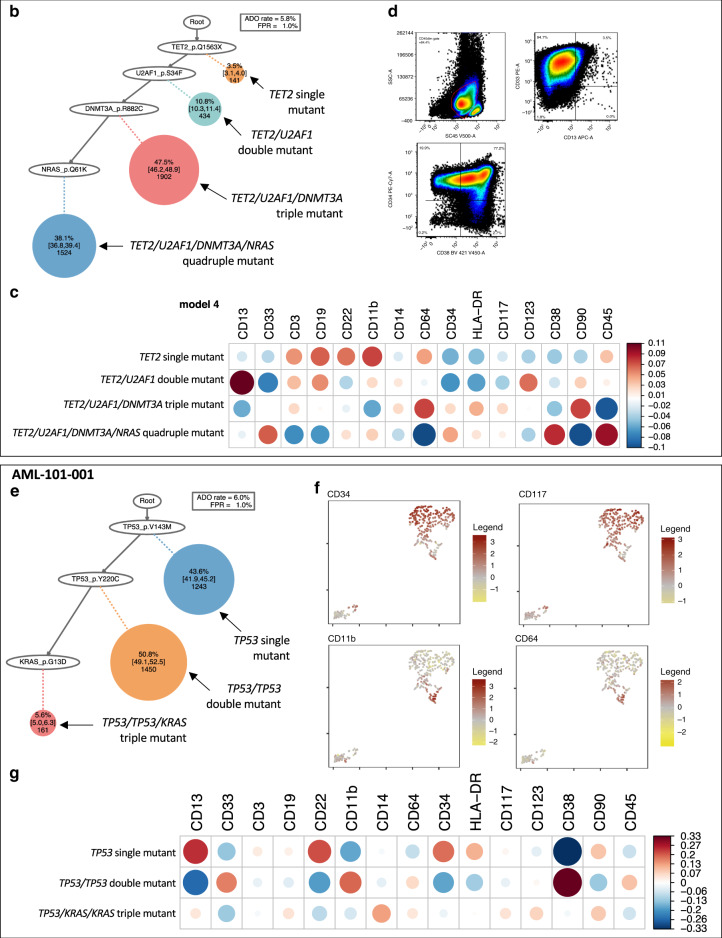
The single-cell genotype–phenotype correlation. **a** A heat map showing the cellular-level correlation between immunophenotype and genotype based on the entire sequenced cells. Each circle is colored by the *r* value of coefficient (red if positively correlated and blue if negatively correlated), with the size reflecting the absolute *r* value (*r < 0.05, **r < 0.01, ***r < 0.001). **b**–**d** A representative case (AML-103-001) showing a stepwise mutation acquisition along with hematopoietic differentiation. **b** SCITE-inferred model 2 phylogeny tree showing a linear evolution pattern of driver mutations. **c** A heat map showing the immunophenotype of each genotype-defined subclone shown in Fig. 5b. **d** Flow cytometry data from the same patient. A cellular population delineated with a red line indicates CD45-dim cells. The blasts were CD34^+^CD33^+^CD13^-^ myeloblasts. A subset of CD34^+^ blasts showed CD38 expression. Detailed flow cytometry data is available in Supplementary Fig. 13c, d. **e**–**g** A representative case (AML-101-001) showing two distinct blasts populations determined by the simultaneous single-cell DNA and protein profiling. **e** SCITE-inferred model 2 phylogeny tree showing a linear evolution pattern. **f** The single-cell immunophenotyping data for selected cell surface markers. Each dot represents a sequenced cell. Relative expression of each cell surface marker is normalized by the degree of the logarithmic odds ratio (log OR, brown if high expression, yellow if low expression). **g** A heat map showing the immunophenotype of each genotype-defined subclone determined by the SCITE model from Fig. 5e. ADO allele dropout, FPR false-positive rate.

To further dissect the intra-tumor heterogeneity in AML, we performed simultaneous profiling of single-cell DNA and cell surface proteins (scDNA+protein-seq) in 26 AML patients using the Tapestri platform (Fig. 5). Immunophenotyping data by scDNA+protein-seq was orthogonally validated by concurrently performed multi-color flow cytometry of the same samples (Supplementary Fig. 13, Methods). We assessed genotype-phenotype correlation across all sequenced cells (Fig. 5a). As expected, wild-type cells were significantly associated with higher expression of CD3 (*r*_s_ = 0.18, *p* < 0.001) and CD19 (*r*_s_ = 0.04, *p* < 0.001), suggesting that most of these cells represent normal T and B lymphocytes, respectively. We also observed that *NPM1* or *IDH* mutations were significantly associated with lower expression of CD34 (*r*_s_ = −0.29 for *NPM1*, *r*_s_ = −0.16 for *IDH1*, *r*_s_ = −0.07 for *IDH2*) and HLA-DR (*r*_s_ = −0.18 for *NPM1*, *r*_s_ = −0.05 for *IDH1*, *r*_s_ = −0.14 for *IDH2*), whereas *TP53* mutations were associated with higher CD34 expression (*r*_s_ = 0.30) (all *p* < 0.001). These data are consistent with the previous findings of the association between these mutations and immunophenotypes^22,23^.

Using the data obtained from mutational history analysis, we then analyzed interplay between genetic and phenotypic evolution in AML. In AML-103-001, *TET2*, *U2AF1*, *DNMT3A*, and *NRAS* mutations were linearly acquired (Fig. 5b). The analysis of cell surface protein expression in each genotype-defined subclone revealed that *TET2* single-mutated cells were associated with both myeloid and lymphoid markers (CD3, CD19, CD22, and CD11b), supporting the preleukemic origin of this mutation^24^. Double mutant (*TET2*-*U2AF1*) cells were still associated with these markers however with lower extent, and also were more strongly associated with early myeloid markers such as CD123 and CD13. Then, triple mutant (*TET2*-*U2AF1*-*DNMT3A*) cells were associated with hematopoietic stem cell markers (CD34 and CD117). Finally, quadruple mutant (*TET2*-*U2AF1*-*DNMT3A*-*NRAS*) cells showed myeloblastic phenotype (CD33^+^, CD34^+^, and CD38^+^, Fig. 4c), which was consistent with the observed blast phenotype by flow cytometry (Fig. 5d). Similarly, AML-101-001 had a linear clonal structure with two different *TP53* mutations and a *KRAS* mutation (Fig. 5e). scDNA+protein-seq identified two phenotypically aberrant populations: one with CD34^+^CD117^+^ myeloblasts and another small population with monocytic differentiation (CD11b^+^CD64^+^) (Fig. 5f). Genotype–phenotype correlation revealed that the cells with single *TP53* mutation (*TP53* p. V143M-mutant) manifested a CD34^+^CD117^+^ phenotype, whereas double *TP53* mutant cells (*TP53* p. V143M and p.Y220C double mutant) were associated with a monocytic immunophenotype (Fig. 5g). These data illustrate a stepwise acquisition of driver mutations in the context of malignant hematopoiesis hierarchy.

### Illustrating clone-by-clone response to AML therapies

**Fig. 6:**
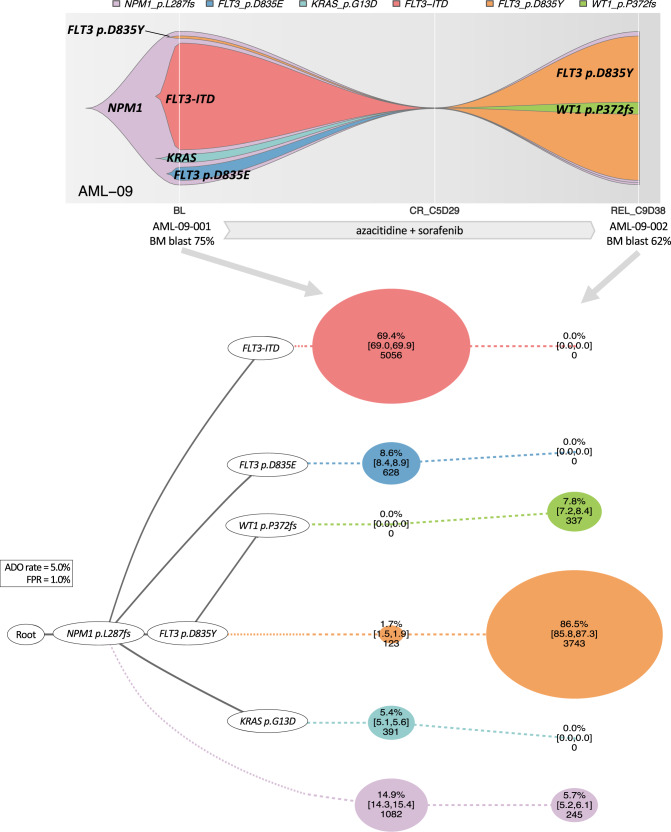
Clonal selection in response to FLT3 inhibitor-containing therapy. A 74-year-old man with newly diagnosed therapy-related acute myelomonocytic leukemia showing a selection of *FLT3* p.D835Y clone during a FLT3 inhibitor-containing therapy. The fish plot shows the inferred clonal evolution pattern based on the single-cell genotype data. The phylogenetic trees visualize the estimated order of mutation acquisition and the proportion of subclones with a different combination of mutations at each timepoint. The wild-type cells which did not carry any driver mutations are not shown. BL baseline, CR complete remission, C cycle, D day, REL relapse, ADO allele dropout, FPR false-positive rate. Full case description is available in Supplementary Methods.

**Fig. 7:**
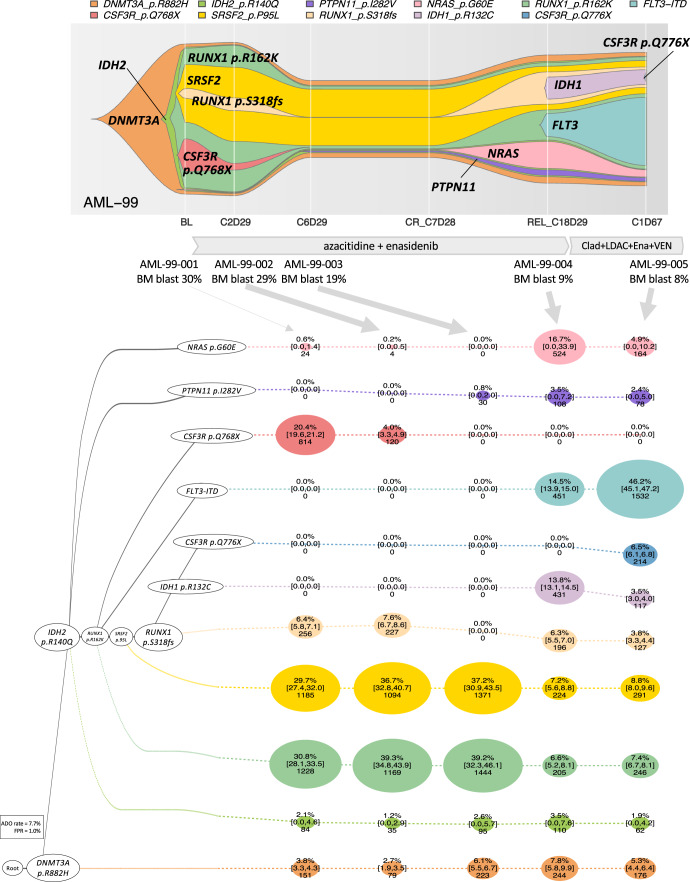
Emergence of *IDH1/FLT3/NRAS* clones during IDH2 inhibitor-containing therapy. A 76-year old woman with AML showing the parallel evolution of *IDH1* p.R132C, *FLT3*-ITD, *NRAS* p.G60E, and *PTPN11* p.I282V clones during an IDH2 inhibitor-containing therapy. Clad cladribine, LDAC low dose cytarabine, Ena enasidenib, VEN venetoclax, DAC decitabine. The fish plot shows the inferred clonal evolution pattern based on the single-cell genotype data. The phylogenetic trees visualize the estimated order of mutation acquisition and the proportion of subclones with a different combination of mutations at each timepoint. The wild-type cells which did not carry any driver mutations are not shown. BL baseline, CR complete remission, C cycle, D day, REL relapse, ADO allele dropout, FPR false-positive rate. Full case description is available in Supplementary Methods.

**Fig. 8:**
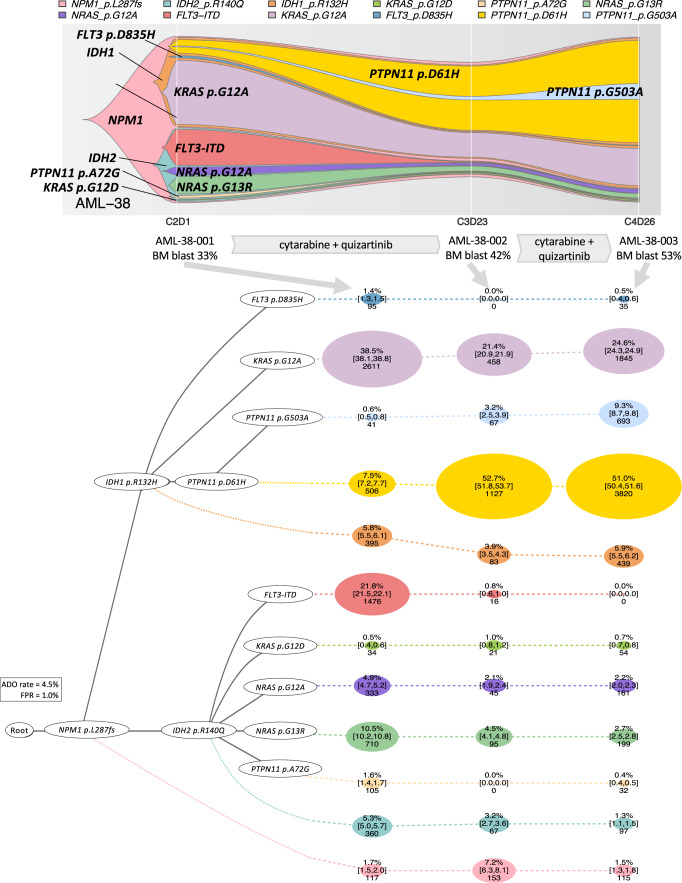
Parallel evolution of *RAS/PTPN11* clones during FLT3 inhibitor-containing therapy. A 58-year-old-man with refractory AML showing the clearance of *FLT3*-ITD clone with an expansion of *PTPN11/RAS* clones during a FLT3 inhibitor-containing therapy. The fish plot shows the inferred clonal evolution pattern based on the single-cell genotype data. The phylogenetic trees visualize the estimated order of mutation acquisition and the proportion of subclones with a different combination of mutations at each timepoint. The wild-type cells which did not carry any driver mutations are not shown. C cycle, D day, ADO allele dropout, FPR false-positive rate. Full case description is available in Supplementary Methods.

**Fig. 9:**
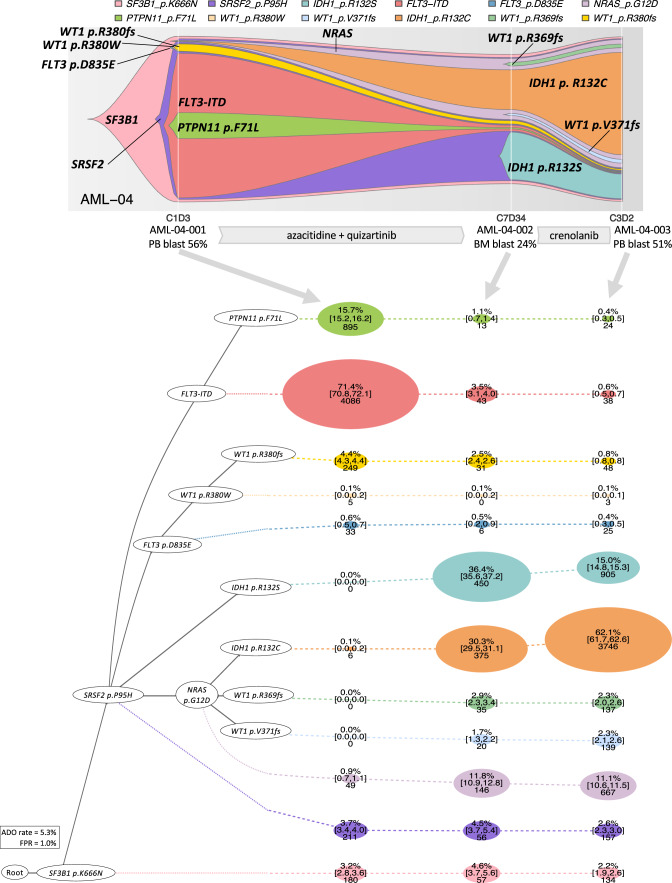
Selection of *IDH1/RAS* clones during FLT3 inhibitor-containing therapy. A 76-year-old man with refractory secondary AML showing the clearance of *FLT3*-ITD clone with an expansion of *IDH/RAS* clones during a FLT3 inhibitor-containing therapy. The fish plot shows the inferred clonal evolution pattern based on the single-cell genotype data. The phylogenetic trees visualize the estimated order of mutation acquisition and the proportion of subclones with a different combination of mutations at each timepoint. The wild-type cells which did not carry any driver mutations are not shown. C cycle, D day, ADO allele dropout, FPR false-positive rate. Full case description is available in Supplementary Methods.

We then analyzed 46 longitudinal samples from 15 patients (13 with baseline and relapse pairs and 2 with multiple refractory timepoints) by scDNA-seq to study the evolution of clonal architecture in response to different therapies (Figs. 6–9 and Supplementary Fig. 14). For instance, in AML-09, we observed a selection of a small subclone with *FLT3* p.D835Y during azacitidine and sorafenib (a FLT3 inhibitor) treatment, which was associated with relapse (Fig. 6). This clonal selection is consistent with the known in vitro differential sensitivity of various *FLT3* mutations to sorafenib^25^; indeed, the *FLT3* p.D835Y mutation is more resistant to sorafenib than the D835E and ITD mutations (subclones with the two mutations were effectively cleared by sorafenib in this patient). Similarly, in AML-99, we observed the selection of subclones with *NRAS* mutation along with the acquisition of *PTPN11*, *FLT3*-ITD, and *IDH1* mutations during treatment with azacitidine and enasidenib (an IDH2 inhibitor). The selection of subclones with RTK/RAS/MAPK signaling pathway mutations as well as *IDH1* mutation is consistent with the previously reported resistance mechanism to IDH2 inhibitor (Fig. 7)^26,27^. The analysis of two treatment-refractory AML cases showed complicated clonal dynamics during therapy. Both AML-38 (Fig. 8, the same case in Figs. 3i and 4c) and AML-04 (Fig. 9) had AML with multiple branching subclones. In both cases, treatment with a FLT3 inhibitor-containing therapy reduced clones with *FLT3* mutations, however, with a concurrent expansion or selection of other clones frequently involving RAS/MAPK signaling pathway mutations, which is in line with a recent study utilizing the same scDNA-seq platform in gilteritinib-treated AML patients^28^. Taken together, scDNA-seq of longitudinal AML samples allowed meticulous illustration of clonal response to therapies that revealed underlying evolutionary dynamics associated with therapeutic resistance.

## Discussion

Using a high-throughput scDNA-seq platform, we have described the landscape of AML clonal architecture with breadth and high resolution. Cell-level mutation co-occurrence and mutual exclusivity data obtained from this study provide a validation for the clonal relationship among AML driver mutations previously inferred by bulk-sequencing studies, but also revealed novel clonal relationships, such as between *TP53* and *PPM1D* that was previously mischaracterized by the population-based analysis^14,15^. Reconstruction of mutational history based on the single-cell data provided evidence for both linear and branching evolution patterns in AML with some cases exhibiting convergent evolution, which is similar to the observations in other studies utilizing multi-region sequencing or single-cell analysis for different tumors^7,20,29–31^. Xenotransplantation of several AML samples including the one with convergent evolution showed leukemia initiating capabilities of multiple parallel subclones, albeit with variable capabilities, consistent with the previous observations that AML LIC populations also consist of genetically diverse cells^7,32^. These clonally diverse LIC populations likely form the basis of emergence and selection of resistant subclones under therapies, a process we have further illustrated meticulously through single-cell analyses of longitudinal specimens (Figs. 6–9 and Supplementary Fig. 14). Using emerging single-cell multi-omics technology, we have simultaneously profiled single-cell mutations and cell surface proteins in AML samples. This analysis allowed correlation of genetic and phenotypic heterogeneity in AML, and also advanced our understanding of how mutation history corroborates with the phenotypic changes during the clonal evolution.

This work represents the largest cohort of AML patients yet examined at single-cell resolution and contributes to a growing body of data^5,7,33^ enabling a deeper understanding of the fundamental clonal architectures of AML. The depth of both patient numbers and cells sequenced allowed a robust analysis of the clonal relationship and phylogeny in this study despite the technical challenges associated with single-cell sequencing, such as ADO, multiplets, coverage inconsistency, false positives, and others. Here, we interrogated up to 37 known leukemia driver genes that have given rise to a remarkable level of clonal complexity in AML. It is noteworthy that this is still an underestimation of the true extent of clonal diversity. Future studies with even more cells, broader coverage of the genome, and integration with single-cell transcriptomic and epigenomic states, which is becoming a reality with recent technological advancements^33–35^, will further elucidate the clonal diversity and evolutionary trajectories of AML. Such studies should be performed ideally in samples collected from a large clinical trials, which would allow systematic investigation of predictive and prognostic impact of clonal diversity in AML.

## Methods

### Patients and samples

We included in the analysis 154 samples (140 BMMCs and 14 peripheral blood mononuclear cells) from 123 patients with AML who had at least one somatic mutation covered by the targeted panel for single-cell DNA sequencing (scDNA-seq). Of the 123 patients, 108 patients were analyzed for the single-timepoint sample collected at pre-treatment (*N* = 98) or relapsed/refractory timepoint (*N* = 10). For the remaining 15 patients, we analyzed the longitudinal samples obtained at pre-treatment and relapse (*N* = 8), pre-treatment, during treatment including remission, and relapse (*N* = 5), and 3 random refractory timepoints (*N* = 2). Among 123 patients, 97 were analyzed by scDNA-seq, 23 were analyzed by the simultaneous single-cell DNA and cell surface protein sequencing (scDNA+protein-seq), and 3 were analyzed by both scDNA-seq and scDNA+protein-seq. All the patients provided written informed consent for sample banking and analysis. We had permission to publish the details of the individual patients. The study was approved by the MD Anderson institutional review board and was in accordance with the Declaration of Helsinki.

### Variant detection by single-cell DNA sequencing

We used a microfluidic approach with molecular barcode technology to amplify the DNA from individual cells. Briefly, cryopreserved BMMCs were thawed, and cells were quantified using a Countess Automated Cell Counter (Thermo Fisher Scientific). The cells were resuspended in cell buffer and diluted to a concentration of 2,000,000–4,000,000 cells/mL. Next, 35–100 μL of cell suspension was loaded onto a microfluidics cartridge and cells were encapsulated on the Tapestri instrument followed by the cell lysis and protease digestion on a thermal cycler within the individual droplet. The cell lysate was then barcoded such that each cell had a unique label^11^. The barcoded samples were then thermocycled using either 50 primer pairs specific to a panel of 19 mutated genes covering known AML-related hotspot loci and 10 commonly heterozygous SNP loci for ADO determination (19-gene panel, Supplementary Table 1), or 279 primer pairs specific to a panel of 37 mutated cancer genes (custom-designed panel, Supplementary Data 2).

The pooled library was sequenced by one of the Illumina’s sequencing platforms (MiSeq, HiSeq 4000, or NovaSeq 6000) with 150- or 250-base pair (bp) paired-end multiplexed runs. Detailed methods are provided in the Supplementary Methods. Briefly, fastq files generated by the sequencers were processed using the Tapestri Analysis Pipeline for adapter trimming, sequence alignment, barcode correction, cell finding, and variant calling. Loom files that were generated by the pipeline via GATK-based haplotype calling were then processed using in-house filtering criteria. We included cells for downstream analysis that met the following criteria for genotyping: total read count (depth, DP) ≥ 10× and alternative allele count ≥3 (scVAF ≥ 15% if 20× ≤ DP ≤ 99×; scVAF ≥ 10% if DP ≥ 100×)^36^. Cells that did not satisfy these criteria were considered to have missing genotypes.

The ADO rate for each sample was calculated on the basis of common SNP information. The VAF from single-cell genotype data (scDNA-seq VAF) was calculated as follows based on the sequencing reads from the pooled single cells: (number of the single-cell sequencing reads with alternate allele)/(number of total single-cell sequencing reads).

### Mutation detection by bulk sequencing

As an orthogonal validation, all samples were concurrently sequenced by conventional bulk next-generation sequencing (bulk-seq) using target-capture deep sequencing (*N* = 111, median coverage: 421×, IQR: 319×–610×) or whole-exome sequencing (*N* = 12, median coverage: 150×, IQR: 86×–160×). Target-capture next-generation sequencing was performed using a SureSelect (Agilent Technologies) custom panel of 297 genes that are recurrently mutated in hematological malignancies (Supplementary Table 3). Briefly, genomic DNA was extracted using an Autopure extractor (QIAGEN/Gentra) and was fragmented and bait-captured in solution according to the manufacturer’s protocols. Captured DNA libraries were then sequenced using a HiSeq 2000 sequencer (Illumina) with 76-bp paired-end reads. Whole-exome sequencing was performed using SureSelect V4 exome probes (Agilent Technologies) and a HiSeq 2000 sequencer (Illumina) with 76-bp paired-end reads. Modified Mutect and Pindel algorithms were used for mutation calling^12^.

### Inference of mutational phylogenies

We used the SCITE software to infer phylogenetic trees of the driver mutations from scDNA-seq data. SCITE implements a statistical model and an MCMC-based Bayesian inference scheme that can be used to find a mutation tree (a partial temporal order of mutations) that best fits the observed single-cell genotypes. Encoding the evolutionary history by a mutation tree (as opposed to a cell lineage tree) makes the use of SCITE particularly efficient for use with our data which is characterized by few mutational events and many cells^19^.

SCITE operates with two parameters, one for the false-positive rate (FPR) and one for the false-negative rate (FNR), which can be either set to predefined values or inferred during MCMC along with the tree structure. We used a global estimate of the sequencing error rate as the FPR (1%) and dataset-specific estimates of the dropout rate (ADO provided by the platform) as the FNR. We ran SCITE separately for each patient, providing the table of mutation calls as the input (encoding zero for wild type, one for mutation, and three for missing data). To obtain a robust model, we ran SCITE with four different combinations of parameters: (1) using all cells including missing genotype information with 1% FPR and SCITE-inferred FNR, (2) using all cells including missing genotype information with 1% FPR and platform-provided FNR, (3) using only cells with full genotype information with 1% FPR and SCITE-inferred FNR, and (4) using only cells with full genotype information with 1% FPR and platform-provided FNR. When provided with an incomplete genotype for a cell, SCITE is still able to use the partial genotyping information in the tree inference and assigns cells into subclones based on the available information. The tree structure was mostly consistent among the 4 models (74 of 123 [60%] cases showing consistent tree structure). Phylogeny figures that are shown in Fig. 2 are based on model 2 (all cells, 1% FPR, and platform-provided FNR). In addition, we have also implemented the use of locus-specific ADO in SCITE. In the absence of locus-specific dropout estimates, we adapted the MCMC scheme to learn ADO rates independently for each mutated locus using the patient-specific estimate as prior mean (SD = 0.002). Tree models using the locus-specific ADO were similar to the ones using locus-independent ADO (Supplementary Fig. 15). Therefore, we report here only trees based on a single locus-independent ADO rate.

To incorporate zygosity states into the phylogeny inference, we represented loci with heterozygous and homozygous mutation states as two separate rows in the table of mutation calls. The first row representing the heterozygous state encodes wild-type and homozygous state at the respective locus as 0, and heterozygous state as 1. The second row representing the homozygous state encodes 0 for wild type and 1 for heterozygous and homozygous state. This encoding is based on the assumption that a homozygous mutation is more likely to emerge from a pre-existing heterozygous mutation, than directly from the wild type. We then ran SCITE on this modified mutation table restricting the MCMC search to phylogenies where the heterozygous state of any mutation precedes its homozygous state.

The inference procedure underlying SCITE is fully Bayesian, which allowed us to quantify uncertainty in the inferred clonal architectures by sampling trees from the model’s posterior distribution. We summarized the sampled trees by reporting 95% credible intervals for each inferred subclone.

For longitudinal samples, we combined the scDNA-seq data from all time points from the same patient and ran SCITE for the pooled data, and reconstructed the tumor phylogeny. When analyzing xenotransplanted samples, we first set the tree structure using human AML samples and assigned regenerated subclones to the tree structure. To obtain time point-specific estimates of subclone sizes, we performed cell-to-subclone assignment in the posterior sampling separately for each time point. As in some cases not all mutations were observed at all time points, we adjusted the assignment probabilities such that a cell cannot be placed below any mutation unobserved at the cell’s sampling time. This leads to subclones with a temporary prevalence of 0%. This does not necessarily mean that the subclone was extinct at that time, but simply reflects the lack of evidence for its existence based on the cells sampled at the respective time point. The number of subclones was defined as the number of distinct cellular populations carrying at least one mutation based on model 2.

B-SCITE was used to infer the phylogeny trees based on the combined data from scDNA-seq and bulk-seq. Briefly, the single-cell data were given as a mutation matrix, and bulk data consisted of the variant and total read count of the mutant loci. B-SCITE reported a single maximum likelihood mutation tree by clustering linear tree segments based on VAF similarity^21^. The TrAp (a tree approach for fingerprinting subclonal tumor composition) algorithm was used to infer phylogenetic trees from bulk-seq data^4^. Fifty patients whose mutations detected by scDNA-seq were all validated by bulk-seq with available read-count data were included.

### SNP array

Genomic DNA from 40 samples in which scDNA-seq data showed at least 5% of homozygously mutated clones were analyzed by Illumina Omni2.5-8 SNP array. The raw data retrieved from an Illumina Omni2.5-8 SNP array was processed using GenomeStudio 2.0. The raw log *R* ratio and B allele frequency were used for allele-specific copy number analysis of tumors algorithm^37^ to identify allele-specific copy-number alterations.

### Droplet digital PCR

We performed droplet digital PCR (ddPCR) using QX200^TM^ Droplet Digital^TM^ System (Bio-Rad Laboratories) to confirm the variants that were detected by scDNA-seq but were not detected by bulk-seq. ddPCR^TM^ Supermix for Probes (No dUTP) was used with 50 ng of genomic DNA as a template for ddPCR assay in a 96-well plate according to the manufacture’s protocol. Seven nanogram of synthesized mutant DNA (designed through Bio-Rad Laboratories and ordered through Integrated DNA Technologies) in a background of 130 ng of normal human genomic DNA (Promega) was used as a positive control. Fifty nanogram of normal human genomic DNA (Promega) was used as a negative control. Water was used instead of DNAs for no-template control reactions. Each reaction was tested in duplicate. Variant-specific primers/probes (ddPCR^TM^ Mutation Detection Assays, FAM/HEX for mutant/wildtype) were designed and ordered through Bio-Rad Laboratories and are summarized in Supplementary Table 4. Data were analyzed using Quanta-Soft Analysis Pro software v1.0.596 (Bio-Rad Laboratories).

### Xenotransplantation using immunodeficient mice

NOD.Cg-Prkdc^scid^ Il2rg^tm1Wjl^ Tg(CMV-IL3,CSF2,KITLG)1^Eav/MloySzJ^ (NSG-SGM3; JAX 013062, The Jackson Laboratory) mice were used for transplanting human bone marrow cells. NSG-SGM3 mice were irradiated at 250 cGy before transplantation. Six AML samples were transplanted into 18 NSG-SMG3 mice (in average 3 mice per sample). Among those, three AML samples (AML-38-001, AML-67-001, AML-41-001) have engrafted in total seven mice (in average two mice each), and we have performed scDNA-seq of the engrafted samples. In addition, the engrafted samples from these 3 cases were transplanted into a total 21 secondary recipient mice, of which 4 engrafted mice from AML-41-001 primary recipients were analyzed by scDNA-seq. All procedures were approved by Baylor College of Medicine Institutional Animal Care and Use Committees.

Peripheral blood samples were collected from NSG-SGM3 recipients and analyzed by flow cytometry for human CD45 to confirm engraftment. Bone marrow cells were isolated by crushing the long bones (tibias and femurs), pelvic bones, and vertebrae with mortar and pestle in Hank’s buffered salt solution without calcium and magnesium, supplemented with 2% heat-inactivated bovine serum (Gibco). Cells were triturated and filtered through nylon screen (100μm, Sefar America) or a 40 μm cell strainer (Thermo Fisher Scientific) to obtain a single-cell suspension. To isolate patient derived cells, cells from peripheral blood and bone marrow were incubated with APC-anti-human CD45 (Clone: HI30, Biolegend). Flow cytometry was performed with FACSAria II (BD Biosciences). The sorted human CD45^+^ cells were then analyzed by the scDNA-seq as described above.

### Simultaneous single-cell mutation/protein profiling

Simultaneous profiling of DNA mutation and cell-surface immunophenotype (scDNA+protein-seq) was performed according to the Mission Bio’s protocol using the custom-designed panel kit and 10–15 oligo-conjugated antibodies (CD13, CD33, CD3, CD19, CD22, CD11b, CD14, CD64, CD34, HLA-DR, CD117, CD123, CD38, CD90, and CD45) purchased from Mission Bio, Inc. CD34 and CD45 oligo-conjugated antibodies were used in a 1:2 dilution, CD33 and HLA-DR in a 1:3 dilution, CD38 in a 1:5 dilution. The remaining oligo-conjugated antibodies were used without dilution. Briefly, cryopreserved BMMCs were thawed, quantified and then stained with the pool of 10–15 oligo-conjugated antibodies. The stained cells were washed and loaded onto the Tapestri^®^ machine for single-cell encapsulation, lysis, and barcoding, following the protocol similar to the scDNA-seq except for adding an extra primer for antibody tags prior to the barcoding. The barcoded samples were then thermocycled. DNA libraries was extracted from the droplets followed by the purification using Ampure XP beads (Beckman Coulter). The supernatant from Ampure XP beads incubation contained antibody-tagged libraries, and was incubated with biotinated oligo (Integrated DNA Technologies) to capture the antibody tags, followed by the purification using streptavidin beads (Thermo Fisher Scientific). The purified DNA and antibody-tagged libraries were indexed and then sequenced on Illumina’s NovaSeq 6000 or NextSeq 500 systems with 150- or 250-bp paired-end multiplexed runs. For the DNA analysis of scDNA+protein-seq, we used the same bioinformatics analysis described in the Supplementary Method. For protein analysis from scDNA+protein-seq, fastq files were processed using the Tapestri Analysis Pipeline followed by the downstream analysis using Tapestri R Package (https://portal.missionbio.com/).

### Multicolor flow cytometry

Immunophenotypes of the bone marrow cells from AML patients were assessed using eight-color flow cytometry on FACSCanto II (BD Biosciences) as part of the routine clinical workup^38^. Briefly, BM cells that were stained with monoclonal antibodies were acquired on FACSCanto II instruments (BD Biosciences). The data were interpreted by in-house board certified hematopathologists.

### Statistics and reproducibility

Categorical variables were compared using chi-squared or Fisher’s exact tests. Continuous variables were analyzed by Student’s *t* tests or Mann–Whitney *U* test depending on the satisfaction of the statistical testing assumptions. Pearson correlation coefficient or Spearman’s rank correlation coefficient (*r*_s_) was used depending on the satisfaction of the statistical testing assumptions.

To evaluate cell-level co-occurrence and mutual exclusivity for individual cases, a contingency table was constructed to compute the log2-transformed odds ratios. Fisher’s exact test was used to evaluate the statistical significance of associations. The Benjamini–Hochberg method was used to adjust for multiple testing^39^.

To evaluate the generalized patterns of cellular-level co-occurrence/exclusivity from the bulk-seq data, the presence or absence of each mutation was recorded for each of the 123 patients. For each pair of mutations, their dependency was summarized as the log odds ratio of their contingency table with Haldane correction. To test for the independence, we computed *G*-statistics and the *p* value from the chi-squared distribution. Adjustment for multiple testing was performed controlling the false discovery rate. To evaluate the generalized patterns of cellular-level co-occurrence/exclusivity based on the scDNA-seq data from the entire cohort, the tumors were resolved at the clonal level. The pairwise dependency among the clones over 1% of a patient’s tumor cells (excluding the cells without any driver mutations) were analyzed.

To assess the clonal diversity, Shannon index was calculated based on the clonal composition determined by the model 2 phylogeny trees in sckit-bio ver 0.5.5 (http://scikit-bio.org/docs/latest/generated/skbio.diversity.alpha.shannon.html#skbio.diversity.alpha.shannon). We considered *P* value of less than 0.05 to be statistically significant. R (ver. 3.4.3) and EZR^40^ software packages were used for statistical analysis.

### Reporting summary

Further information on research design is available in the Nature Research Reporting Summary linked to this article.

## Supplementary information

Supplementary Information

Peer Review File

Description of Additional Supplementary Files

Supplementary Data 1

Supplementary Data 2

Reporting Summary

## Data Availability

Single-cell sequencing data and bulk sequencing data have been deposited at NCBI BioProject ID PRJNA648656, and SNP array data with NCBI GEO ID GSE156934. The remaining data are available within the article, supplementary information or from the author upon request.

## Code availability

In-house codes that were used for single-cell sequencing data variant calling are available at [https://github.com/farmerkingwf/Single_Cell_Codes.git].
