# Author Correction: Clonal evolution of acute myeloid leukemia revealed by high-throughput single-cell genomics

**DOI:** 10.1038/s41467-021-23280-z

**Published:** 2021-05-10

**Authors:** Kiyomi Morita, Feng Wang, Katharina Jahn, Tianyuan Hu, Tomoyuki Tanaka, Yuya Sasaki, Jack Kuipers, Sanam Loghavi, Sa A. Wang, Yuanqing Yan, Ken Furudate, Jairo Matthews, Latasha Little, Curtis Gumbs, Jianhua Zhang, Xingzhi Song, Erika Thompson, Keyur P. Patel, Carlos E. Bueso-Ramos, Courtney D. DiNardo, Farhad Ravandi, Elias Jabbour, Michael Andreeff, Jorge Cortes, Kapil Bhalla, Guillermo Garcia-Manero, Hagop Kantarjian, Marina Konopleva, Daisuke Nakada, Nicholas Navin, Niko Beerenwinkel, P. Andrew Futreal, Koichi Takahashi

**Affiliations:** 1 Department of Leukemia, The University of Texas MD Anderson Cancer Center, Houston, TX USA; 2 Department of Hematology and Oncology, Graduate School of Medicine, The University of Tokyo, Tokyo, Japan; 3 Department of Genomic Medicine, The University of Texas MD Anderson Cancer Center, Houston, TX USA; 4 Department of Biosystems Science and Engineering, ETH Zurich, Basel, Switzerland; 5 SIB Swiss Institute of Bioinformatics, Basel, Switzerland; 6 Department of Molecular and Human Genetics, Baylor College of Medicine, Houston, TX USA; 7 Department of Hematopathology, The University of Texas MD Anderson Cancer Center, Houston, TX USA; 8 Department of Neurosurgery, The University of Texas Health Science Center at Houston, Houston, TX USA; 9 Department of Oral and Maxillofacial Surgery, Hirosaki University Graduate School of Medicine, Aomori, Japan; 10 Department of Genetics, The University of Texas MD Anderson Cancer Center, Houston, TX USA; 11 Department of Bioinformatics and Computational Biology, The University of Texas MD Anderson Cancer Center, Houston, TX USA

**Keywords:** Cancer genomics, Acute myeloid leukaemia, Tumour heterogeneity

Correction to: *Nature Communications* 10.1038/s41467-020-19119-8, published online 21 October 2020.

The original version of this Article incorrectly attribute the NIH CA193235 grant to the Leukemia and Lymphoma Society. The correct attribution is the National Institute of Health (NIH R01CA193235). In addition, the following was omitted from the Acknowledgements: Cancer Prevention and Research Institute of Texas training grant (RP160283) (to T.H.). These errors have now been corrected in both the PDF and HTML versions of the Article.

