# Supplementary material for: Clonal evolution of acute myeloid leukemia revealed by high-throughput single-cell genomics
Source: Nat Commun. 2020 Oct 21;11:5327. doi: 10.1038/s41467-020-19119-8 (PMC7577981; doi:10.1038/s41467-020-19119-8)
Supplement: Supplementary file 1 — Supplementary Information [file 41467_2020_19119_MOESM1_ESM.pdf]

Supplementary Information

Supplement to: Morita K, Wang F, Jahn K, et al. Clonal Evolution of Acute Myeloid Leukemia Revealed by High-Throughput Single-Cell Genomics.

Contents

Supplementary Methods

Supplementary Table 1
Supplementary Table 2
Supplementary Table 3
Supplementary Table 4

Supplementary Figure 1
Supplementary Figure 2
Supplementary Figure 3
Supplementary Figure 4
Supplementary Figure 5
Supplementary Figure 6
Supplementary Figure 7
Supplementary Figure 8
Supplementary Figure 9
Supplementary Figure 10
Supplementary Figure 11
Supplementary Figure 12
Supplementary Figure 13
Supplementary Figure 14
Supplementary Figure 15

Supplementary References

Supplementary Methods

Variant calling and genotyping using single-cell DNA sequencing data

Fastq files generated by an Illumina sequencer were processed using the Tapestri Analysis Pipeline. Adapter sequences were trimmed from the sequenced reads using Cutadapt [1,2]. Reads that were too short were discarded, and the Tapestri Barcode structures were extracted from the reads. The reads were then mapped to human genome version hg19 using the BWA-MEM algorithm [3] [4]. The extracted barcodes on the mapped reads were error-corrected against a whitelist of known barcodes using a hamming distance approach. Reads lacking an insert sequence between gene-specific primers or mapped to off-target loci were discarded. The barcodes were identified as cells using a 2-step process. First, amplification patterns of all amplicons were reduced to principal components to separate barcodes with high read-load (putative cells) from barcodes with low read-load (noise). Then a coverage parameter of at least 10 reads for at least 60% of the amplicons was used for each barcode to discard putative cells with low data completeness from cells with high data completeness.

The cells were genotyped with the Genome Analysis Toolkit [5] using a joint calling approach that followed GATK Best Practices recommendations [6,7]. Each cell was haplotyped in reference confidence mode to enable per-base pair (bp) confidence estimates for a site's being strictly homozygous (reference). The per-bp resolution was maintained while merging the genomic-VCFs (gVCFs) for all cells using GATK's CombineGVCFs tool. Finally, joint genotyping was performed for all cells using GATK's GenotypeGVCFs tool. Loci found to be nonvariant were maintained in the final output. Genotyping parameters were optimized for high sensitivity: a maximum of 2 alternate alleles were reported for each site, the minimum base quality for variant calling was set at 10, and the heterozygosity value was set at 0.001.

Internal tandem duplications in the *FLT3* gene were identified using a custom genotyping method. We looked for insertions in the *FLT3* amplicons and mapped them to the locus. If the read did not go through the insertion, our mapper soft-clipped it at the end. For each cell, we scanned for these soft-clips and insertions; all insertions and clippings were considered as possible insertions. If the total number of reads was greater than a cutoff (10), and the number and the ratio of non-reference reads were greater than a cutoff (4 and 0.1 respectively), the cell was considered to have a non-reference allele. If the ratio of non-reference reads was greater than a cutoff (0.9), a homozygous event was called; otherwise it was considered a heterozygous event. If the cell had enough total reads but not enough alternate reads, it was considered a homozygous reference. Otherwise, it was reported as "no call." Multiallelic variants were decomposed into biallelic variants and then normalized to ensure that each VCF entry was left-aligned and parsimonious [8]. Blacklisted loci were filtered out, and all loci with a quality value <1000 were tagged for downstream processing. The positions that passed our filtering criteria were called as variants. The genotypes and the cell matrix were converted into an open-source loom format [9], which allows efficient storage, data retrieval, and sharing of large omics data sets.

Allele dropout (ADO) was calculated based on the fraction of cells that were genotyped as not heterozygous at a specific locus that is expected to be heterozygous in the general population. In Mission Bio's pre-designed 19-gene panel, 10 amplicons were dedicated specifically to cover the highly heterozygous loci. In custom-designed panel, highly heterozygous loci were obtained among the variants detected in the regions covered by the amplicons. Non-heterozygous genotype is either homozygous reference genotype when the mutant allele failed to amplify, or homozygous genotype of the alternate allele when a reference allele failed to amplify. ADO rate was defined based on the variant allele frequency, which was

calculated as (number of cells with non-heterozygous genotype at known heterozygous locus) / (number of total sequenced cells) × 0.5.

We estimated the doublet rate of Tapestri system using a 50:50 mixture data of K562 and Raji cell lines that was provided by Missino Bio, Inc. We investigated 4 loci with known truth genotype that are distinct between these cell lines for cell assignment. We assigned cells that were homozygous reference at the 4 loci to Raji, cells that were homozygous alternate at the 4 loci to K562 and cells that are heterozygous at these loci to doublet. The mean doublet rate based on the 45 Tapestri runs with 50:50 K562 Raji mixture was 5.86% with a standard deviation of 2.43%. This doublet rate correlates with the total number of cells to the bead ratio.

Case description for longitudinal single-cell analysis

AML-09 (Fig. 6): A 74 year-old-patient who had undergone radiation therapy for prostate cancer was diagnosed with therapy-related AML (AML-09-001). The patient was found to have *FLT3*-ITD and kinase domain (D835) mutations and was started on induction therapy consisting of azacitidine and sorafenib. The patient achieved complete remission, but the disease recurred after 9 cycles (AML-09-002). The patient was started on sorafenib and was lost to follow-up. The *FLT3*-ITD mutation that was found in 70% of cells at diagnosis as well as small independent branched subclones with *FLT3* p.D835E or *KRAS* mutations were cleared after azacitidine plus sorafenib therapy, whereas a small subclone with an *FLT3* p.D835Y mutation significantly expanded and acquired additional mutation in *WT1* at the time of relapse.

AML-99 (Fig. 7): A 76-year-old patient was diagnosed with AML with maturation (AML-M2) with *IDH2* R140Q mutation (AML-99-001). The patient was started on induction therapy with azacitidine and enasidenib. After 7 cycles of azacitidine and enasidenib therapy (AML-99-002 obtained after 2 cycles, AML-99-003 obtained after 6 cycles), the patient achieved complete

remission with incomplete platelet recovery. After approximately one year of remission, the patient had a relapse after 18 cycles of azacitidine and enasidenib therapy therapy (AML-99-004). Upon completion of a total 21 cycles of azacitidine and enasidenib therapy, the patient was switched to salvage chemotherapy with cladribine, low-dose cytarabine, enasidenib, and venetoclax (Clad+LDAC+Ena+VEN) but was refractory (AML-99-005). Since the patient was found to have *FLT3*-ITD, the patient was started on enasidenib plus gilteritinib therapy, but progressed. Due to the expansion of emerging *IDH1* clone together with persistent *IDH2* clone, the patient was then switched to decitabine, venetoclax, plus quizartinib, and has not yet achieved remission. *NRAS, PTPN11, FLT3-ITD*, and *IDH1* mutations that were undetectable at diagnosis were acquired during azacitidine and enasidenib therapy.

AML-38 (Fig. 8): A 58-year-old-patient with refractory AML, who had been treated with azacitidine and decitabine at an outside institution, was referred to MD Anderson. The patient was found to have *FLT3*-ITD and was started on cytarabine and quizartinib. The patient achieved complete remission with incomplete platelet and neutrophil recovery after 1 cycle. The patient was found to have recurrent disease prior to the start of the second cycle (AML-38-001), which had been delayed because the patient experienced prolonged myelosuppression. The patient received a total of 4 cycles of cytarabine and quizartinib therapy (AML-38-002, AML-38-003), but the disease was refractory. The patient received 2 cycles of chemotherapy consisting of fludarabine, cytarabine, and sorafenib, which markedly reduced the bone marrow blast percentage, from 53% to 7%. The patient then underwent an allogeneic stem cell transplant from a matched unrelated donor. The patient died of pneumonia approximately 1 year after transplant, even though the patient's AML remained in remission. After cytarabine and quizartinib therapy, *FLT3*-ITD clone became undetectable, whereas the remaining clones persisted or expanded.

AML-04 (Fig. 9): A 76-year-old patient who had undergone brachytherapy and external-beam radiotherapy for prostate cancer presented with secondary AML arising from essential thrombocythemia, which had been refractory to decitabine and ruxolitinib (AML-04-001). The patient was found to have *FLT3*-ITD mutation and was treated with azacitidine plus quizartinib. The bone marrow blast percentage markedly decreased, from 40% to 11%, after 2 cycles, but no further response to the treatment was observed after 7 cycles (AML-04-002). The patient was then treated with 3 cycles of crenolanib, but the disease remained refractory (AML-04-003). The patient began azacitidine plus sorafenib therapy but died of unknown causes after approximately 5 months. The *SF3B1-SRSF2-FLT3*-ITD clone that was the dominant clone at the first time point substantially shrank after treatment with azacitidine plus quizartinib, whereas the *SF3B1-SRSF2-NRAS-IDH1* p.R132C clone and *SF3B1-SRSF2-IDH1* p.R132S clone emerged and expanded. The *SF3B1-SRSF2-FLT3*-ITD clones further shrank after crenolanib therapy, whereas the remaining clones persisted or expanded. Two founder mutations (*SF3B1* and *SRSF2*) persisted during both therapies.

AML-01 (Supplementary Fig. 14): A 66-year-old patient with a history of radiation treatment for prostate cancer was diagnosed with AML with minimal maturation (AML-M1) with *FLT3*-ITD (AML-01-001). The patient was treated with cladribine plus low-dose cytarabine alternating with decitabine and achieved complete remission with incomplete platelet recovery after 2 cycles. The patient received additional 2 cycles of consolidation therapy, but the disease relapsed and was positive for *FLT3*-ITD (AML-01-002). The patient was then treated with azacitidine and sorafenib. Although the patient achieved complete remission with incomplete platelet recovery after 1 cycle, the patient developed *Mucor* cellulitis. The patient died of multiorgan failure approximately 9 months after the diagnosis of therapy-related AML. The clonal architecture was

similar in the pretreatment and relapse samples.

AML-07 (Supplementary Fig. 14): A 75-year-old patient was diagnosed with pure erythroid leukemia (AML-M6B, AML-07-001). The patient received induction chemotherapy with clofarabine plus low-dose cytarabine and achieved complete remission after 1 cycle. The patient was found to have a relapse after receiving 3 cycles of consolidation therapy (AML-07-002). Five cycles of salvage chemotherapy consisting of fludarabine and cytarabine failed to induce remission. The patient was then started on clofarabine, azacitidine, and low-dose cytarabine and achieved remission but relapsed approximately 2 years later. The patient was given 5 cycles of azacitidine with no response. The patient was switched to clofarabine followed by cytarabine and initially responded well, but the disease later progressed. The patient died of unknown causes approximately 6 years after the diagnosis of AML. A subclone with *RUNX1* mutations, which was estimated to have occurred at the latest evolutionary stage, substantially shrank at relapse, whereas the 2 founder mutations (*IDH2* and *SRSF2*) were persistent, showing the differential chemosensitivity of mutations.

AML-18 (Supplementary Fig. 14): A 30-year-old patient was diagnosed with AML with maturation (AML-M2, AML-18-001). The patient was treated with induction chemotherapy with clofarabine, idarubicin, and cytarabine (CIA). The patient achieved remission and completed 6 cycles of consolidation therapy, but had a relapse after approximately 1 year of remission (AML-18-002). The relapsed disease was refractory to multiple regimens, including vosaroxin/placebo plus cytarabine, guadecitabine, and CIA. The patient underwent an allogeneic stem cell transplant from a matched unrelated donor, and achieved complete remission. The patient received azacitidine maintenance therapy and a donor leukocyte infusion, but had a relapse approximately 6 months after stem cell transplant. The disease was refractory to multiple

regimens including evofosfamide, buparlisib, decitabine, AZD-1208, erlotinib, PRI724, decitabine plus cytarabine, IGN523, fludarabine plus cytarabine, enasidenib, uprosertib plus trametinib, and APTO253. The patient developed CNS leukemia and myeloid sarcoma and died approximately 4 years after the diagnosis of AML. The clonal architecture was similar in the pretreatment and relapse samples.

AML-21 (Supplementary Fig. 14): A 56-year-old patient was diagnosed with acute myelomonocytic leukemia (AML-M4, AML-21-001). The patient was treated with induction chemotherapy with clofarabine, idarubicin, and cytarabine, followed by 4 cycles of consolidation therapy. After approximately 7 months of remission, the patient was found to have a relapse (AML-21-002). The patient was started on vosaroxin/placebo plus cytarabine, followed by fludarabine plus cytarabine, and died approximately after 1 year after the diagnosis of AML. While both baseline and relapse samples shared the ancestral *WT1* p.A382fs -*NPM1* mutations, the *NRAS* clone was replaced by the *FLT3*-ITD-*WT1* p.S381fs clone at relapse. The *FLT3*-ITD and *WT1* p.S381fs mutations were undetectable at baseline. They were likely acquired *de novo* at relapse, or sub-detectable at baseline and were selected during the therapy.

AML-39 (Supplementary Fig. 14): A 55-year-old patient with a history of myelodysplastic syndrome experienced a progression into secondary AML (AML-39-001). The disease was refractory to induction chemotherapy with clofarabine, idarubicin, and cytarabine, and the patient was switched to decitabine. The patient achieved complete remission with incomplete platelet and neutrophil recovery, but relapsed after the second cycle (AML-39-002). The patient was treated with cyclophosphamide, etoposide, carboplatin, and cytarabine, but died approximately 6 months after the diagnosis of AML. The 2 *PTPN11* mutations comprised independent branching subclones that shared *SF3B1* and *GATA2* mutations. The clone with the *PTPN11* p.A72V

mutation was cleared at relapse, whereas the *PTPN11* p.G503A mutation persisted, illustrating the selection of *PTPN11* p.G503A clone over *PTPN11* p.A72V clone under the selective pressure of treatment.

AML-63 (Supplementary Fig. 14): A 65-year-old patient was diagnosed with AML with minimal maturation (AML-M1) with *FLT3*-ITD (AML-63-001). The patient was treated with induction therapy consisting of decitabine and vosaroxin (AML-63-002). The patient achieved complete remission after 1 cycle (AML-63-003). After an additional cycle of decitabine and vosaroxin, the patient was found to have relapsed (AML-63-004). The patient continued to receive decitabine and vosaroxin, and achieved complete remission with incomplete neutrophil recovery (AML-63-005). After a total of 6 courses of decitabine and vosaroxin therapy, the patient underwent an allogeneic stem cell transplant from a matched related donor. The patient remains in remission 3 years after the transplant. *IDH2- NPM1* clone acquired *KIT* or *FLT3* mutations in parallel. Decitabine and vosaroxin therapy suppressed *KIT* or *FLT3* clones, whereas *IDH2* and *NPM1* mutations survived the therapy, illustrating the differential sensitivity of mutations to the therapy with hypomethylating agent.

AML-66 (Supplementary Fig. 14): A 70-year-old patient was diagnosed with acute myelomonocytic leukemia (AML-M4, AML-66-001). The patient was treated with guadecitabine (AML-66-002) and achieved complete remission with incomplete platelet and neutrophil recovery after 3 cycles, but relapsed after approximately 1 month (AML-63-003). The patient's disease was refractory to salvage therapies, including azacitidine plus nivolumab and venetoclax plus idasanutlin, and the patient died approximately 1 year after the diagnosis of AML. The clonal architecture was unchanged from baseline to post-treatment with guadecitabine.

AML-83 (Supplementary Fig. 14): A 61-year-old patient was diagnosed with AML with *IDH2* p.R172K mutation (AML-83-001). The patient was started on induction therapy consisting of azacitidine plus enasidenib. The patient completed 3 cycles of azacitidine plus enasidenib therapy. Azacitidine was held during the 4th cycle, due to prolonged cytopenia. The patient achieved complete remission with incomplete platelet and neutrophil recovery after 4 cycles, but approximately one month later, the patient was found to have relapsed. The patient received salvage therapy with decitabine and venetoclax. The patient achieved complete remission with incomplete platelet and neutrophil recovery after one cycle. The patient continued to receive the therapy at a local hospital, where the patient was found to have circulating blasts with CNS involvement. The patient was then transferred to MD Anderson, and was confirmed to have relapsed (AML-83-002). The patient was refractory to the salvage chemotherapy comprising fludarabine, cytarabine, and pegfilgrastim combined with intrathecal cytarabine. The patient's AML was then managed with supportive care at a local hospital. Two *NRAS* mutations that were undetectable at initial diagnosis were acquired at relapse.

AML-88 (Supplementary Fig. 14): A 60-year-old patient was diagnosed with AML with *FLT3*-ITD and *FLT3* p.D835V mutations (AML-88-001). The patient was treated with induction chemotherapy with cladribine, idarubicin and cytarabine (CLIA) plus midostaurin. The patient achieved complete remission with incomplete platelet and neutrophil recovery after one cycle. The patient remained in complete remission with incomplete platelet and neutrophil recovery after a total of 3 cycles of CLIA plus midostaurin (CLIA+mido) therapy (AML-88-002). The patient then underwent an allogeneic stem cell transplant (allo-SCT) from a matched unrelated donor. Bone marrow aspiration at day34 post allo-SCT showed complete remission with complete count recovery (AML-88-003), but unfortunately, the patient had a relapse

approximately 2 months after the transplant with positive *FLT3*-ITD (AML-88-004). The patient was refractory to salvage therapy with decitabine plus quizartinib (DAC+quizartinib). The patient was then switched to decitabine, venetoclax, and gilteritinib (DAC+VEN+gilteritinib), and achieved complete remission with incomplete platelet and neutrophil recovery after one cycle (AML-88-05). Although the patient remained in remission after the second cycle of therapy in which decitabine was omitted due to prolonged neutropenia, the patient developed acute kidney injury and expired approximately one year after the diagnosis of AML. *FLT3* p.834_835del and *FLT3* p.D835V mutations that are estimated to have been acquired at the final stage of leukemia evolution became undetectable after CLIA plus midostaurin therapy, and remained undetectable during the subsequent therapy. *NPM1, DNMT3A,* and *TET2* mutations as well as *FLT3*-ITD persisted at remission after CLIA plus midostaurin therapy. They became undetectable after allo-SCT, but later re-emerged at the time of relapse. *SF3B1* mutation that was estimated to have been acquired at the earliest stage of leukemia evolution grossly persisted at remission even after allo-SCT.

AML-97 (Supplementary Fig. 14): A 70-year-old patient was diagnosed with acute monoblastic leukemia (AML-M5, AML-97-001). The patient received induction therapy with decitabine and venetoclax (DAC+VEN), and achieved complete remission with incomplete neutrophil recovery after one cycle (AML-97-002). The patient remained in complete remission with incomplete neutrophil recovery after the second cycle (AML-97-003). After a total of 3 cycles of DAC+VEN therapy, the patient achieved complete remission with complete count recovery (AML-97-004), and underwent an allogeneic stem cell transplant from a matched related donor. Although bone marrow examination one month after the transplant showed no morphological features of residual leukemia (AML-97-005), the patient had a relapse

approximately 5 months after transplant with positive *FLT3*-ITD (AML-97-006). The patient was started on salvage therapy with azacitidine and quizartinib and achieved complete remission with incomplete platelet recovery after one cycle. After a total of 2 cycles, the patient remained in remission, and was transferred to a local hospital. *NPM1* and *NRAS* mutations became undetectable after 2 and 3 cycles of DAC+VEN therapy, respectively, whereas the trunk *DNMT3A* mutation grossly persisted at remission after chemotherapy or allo-SCT, suggesting the differential chemosensitivity of each mutation. *WT1* and *FLT3* mutations that were undetectable at initial diagnosis were acquired at relapse.

AML-107 (Supplementary Fig. 14): A 76-year-old patient who had undergone chemotherapy, radiation, and autologous stem cell transplant for Hodgkin lymphoma presented with secondary AML arising from therapy-related myelodysplastic syndromes, which had been refractory to cladribine, cytarabine plus filgrastim, decitabine, and gemtuzumab ozogamicin (AML-107-001). The patient was started on decitabine plus venetoclax therapy. The patient achieved complete remission with incomplete platelet and neutrophil recovery after two cycles but was found to have a relapse approximately after two weeks. The patient was refractory a total 4 cycles of decitabine plus venetoclax therapy (AML-107-002 obtained after 3 cycles). The patient received fludarabine, cytarabine and NK cells but his leukemia further progressed, and died approximately 6 months after the diagnosis of AML. The clonal architecture was unchanged from baseline to post-treatment with decitabine plus venetoclax.

Supplementary Table 1. List of 50 amplicons covered by the 19-gene panel

Amplicon	Chr	Primer Start (based on hg19)	Insert Start (based on hg19)	Insert End (based on hg19)	Primer End (based on hg19)	forward primer sequence	reverse primer sequence
ASXL1_1	20	31022348	31022369	31022586	31022608	CAGGACCCTCGCAGACATTAAA	GGCAGTAGTTGTGTTCGCTGTA
ASXL1_2_a1	20	31022880	31022899	31023110	31023130	TGTGAGTCTGGCACCACTTC	CATGAGCCACCAAGCCCTAA
DNMT3A_10	2	25457114	25457135	25457351	25457372	TTTGTGTCGCTACCTCAGTTTG	GGTCCTGCTGTGTGGTTAGAC
EZH2_1	7	148504627	148504654	148504874	148504901	TGCAAATTCAGAATTTCAAACTGCATGT	CATTTTAATGCACCCACTATCTTCAGC
EZH2_2	7	148506303	148506332	148506547	148506577	CTGACTTGTTCACATAACAAACAACTATCC	AGAACTGTAACCAGTTGCATTTACAAAATC
FLT3_1	13	28592473	28592494	28592723	28592747	GCAGACTGCTGTGAGGGTTTTT	CTCTGGTGTCATTCTTGACAGTGT
FLT3_2_a3	13	28608168	28608191	28608368	28608392	TTCCAATGGAAAAGAAATGCTGCA	AACTGCCTATTCCTAACTGACTCA
FLT3_3	13	28602155	28602179	28602404	28602429	GAGTGCTCAGTGTCTAATTCCACTT	ACAGAAAAAGCAGACAGCTCTGAAA
FLT3_4	13	28609521	28609547	28609769	28609795	ACACTGACCCTATACTCTCCTGTAAAA	CACAGAAGGAGTCTGGAATAGAAAGG
FLT3_5_4	13	28607997	28608018	28608153	28608176	TGTTGCTGTCCTTCCACTATAC	CCATTGGAAAATCTTTAAAATGC
GATA2_1	3	128202704	128202723	128202891	128202911	AGTCTTCGCTTGGGCTTGAT	GGACTCCCTCCCGAGAACTT
IDH1_1	2	209112875	209112898	209113123	209113149	AATGTGTTGAGATGGACGCCTATT	CTTGTGAGTGGATGGGTAAAACCTAT
IDH2_1_4	15	90631738	90631759	90631985	90632009	CAGAGACAAGAGGATGGCTAGG	GTGGGACCACTATTATCTCTGTCC
JAK2_1	9	5073541	5073563	5073785	5073815	GCAGGTCCATATAAAGGGACCAA	AGGCATTAGAAAGCCTGTAGTTTTACTTAC
KIT_1	4	55599204	55599231	55599448	55599478	AAATGGTTTTCTTTTCTCCTCCAACCTA	CTAAAATGTGTGATATCCCTAGACAGGATT
KIT_2	4	55589585	55589607	55589829	55589859	CCTCCTTGTACCTTCCACTCCTT	CTCAGTTCCTGGACAAAAATACCAATCTAT

KRAS_1	12	25398161	25398183	25398405	25398435	AAAGAATGGTCCTGCACCAGTAA	AAAGGTGAGTTTGTATTAAAAGGTACTGGT
KRAS_2	12	25380238	25380259	25380466	25380490	TCCTCATGTACTGGTCCCTCAT	CGTCATCTTTGGAGCAGGAACAAT
NPM1_1_2	5	170837385	170837412	170837636	170837659	TTCTTGGAGTCATATCTTTATCTAGAGT	TCTGCATTATAAAAGGACAGCC
NRAS_1	1	115256296	115256324	115256546	115256570	ACAACCTAAAACCAACTCTTCCCATAATT	TGGTGAAACCTGTTTGTTGGACAT
NRAS_2	1	115258525	115258553	115258776	115258799	CACGTTAAGCTTATTGCATAACTGAATGT	GGTTCTTGCTGGTGTGAAATGAC
PTPN11_1_1	12	112926827	112926848	112927043	112927063	GGTGTTGACTGCGATATTGACG	CCTGTCCTCCTGCTCAAAAG
PTPN11_2	12	112888095	112888116	112888327	112888351	GCCTCCCTTTCCAATGGACTAT	GCAGCAGACTTTGTGGTCACTAAA
RUNX1_2	21	36171458	36171482	36171710	36171732	CATGGGACTCAGAGTAGAGATAGGT	CGTGGTCCTACGATCAGTCCTA
RUNX1_3	21	36206684	36206705	36206884	36206907	AGTGGGCTCCATCTGGTACTTA	CCACAATAGGACATCGGCAGAAA
RUNX1_4	21	36231583	36231604	36231834	36231857	CTCAGTGCACAGAAACAAGCTT	CCATCACTGTCTTCACAAACCCA
RUNX1_5	21	36164768	36164785	36164997	36165018	CGACATGCCGATGCCGAT	CCCATCCTCCTAGGCGGTATC
RUNX1_7	21	36252789	36252811	36253007	36253030	AATTTTGAAATGTGGGTTTGTTG	GTCCTTTGACTGGTGTTTAGGTG
SF3B1_1	2	198266733	198266761	198266977	198267007	CTTCCATAAAGGCTTTAACACAGAATCAA	GCTATGGTTCATGTTTTGCTTTTACCTAAT
SF3B1_2	2	198267134	198267156	198267384	198267406	TGTGTGTGTACCTCTAGTCCCAA	GTGTGCAAAAGCAAGAAGTCCT
SRSF2_2_2	17	74732865	74732882	74733050	74733069	CCTCAGCCCCGTTTACCT	CTTCGTTCGCTTTCACGAC
TP53_1	17	7578062	7578086	7578296	7578319	GGGTTATAGGGAGGTCAAATAAGCA	GGCCTCTGATTCCTCACTGATTG
TP53_2	17	7577376	7577397	7577615	7577637	TGTGATGAGAGGTGGATGGGTA	CCTCATCTTGGGCCTGTGTTAT
TP53_3	17	7578363	7578384	7578605	7578627	CTGCTCACCATCGCTATCTGAG	TGCCGTCTTCCAGTTGCTTTAT

TP53_4	17	7576930	7576953	7577180	7577204	GGAAAGAGGCAAGGAAAGGTGATA	GACCTGATTTCCTTACTGCCTCTT
U2AF1_1	21	44514679	44514700	44514922	44514947	GGTGGGTTGGAAGGAGACATTT	AGTCTTATTAAAGCGTGGATGGCAA
U2AF1_2	21	44524258	44524279	44524505	44524532	AGTCGATCACCTGCCTCACTAT	GCTCTCATTTTCCCTTACAGAGTCAAC
WT1_1_a2	11	32414174	32414193	32414411	32414432	GAACACAGCTGCCAGCAATG	CCTACCCTAACAAGCTCCAGC
WT1_2	11	32413389	32413415	32413641	32413663	TCCTTCTCTCAACTGAGTCTAAACCTT	CTCACTGTGCCCACATTGTTAG
WT1_3	11	32417744	32417765	32417992	32418018	GCCTGGAAAAGGAGCTCTTGAA	TCAAGACCTACGTGAATGTTCACATG
chr10_106721610	10	106721487	106721508	106721711	106721736	GCTGACTGCCCTTATTGAGATG	ACTTTGCCACCTTGATATTATGTTC
chr10_5554293	10	5554171	5554192	5554401	5554419	CCCTAACCATCGTTCCTTCAGG	GGAAACGGGGTGTGCGAA
chr10_77210191	10	77210064	77210083	77210294	77210313	ATGGAGATCAGCTGCTTGCC	TTAACACCGCCTCTCCTGC
chr14_56969005	14	56968884	56968905	56969106	56969129	CAGAGTCCTCTCCAGGGTAAGA	ACCAAATGCAAATACCAGGATGA
chr16_55770629	16	55770512	55770529	55770735	55770757	TCCAGTGCTCCCAGGCAT	GTGGTGAGGAGATCAGGAGGAT
chr16_8569820	16	8569695	8569720	8569926	8569944	ATTTCATGACCACTCTATTTCTTTCT	CATGGACATGGCCTGCAC
chr18_9750662	18	9750543	9750561	9750767	9750791	CGGATTGGCCAGTGCATTC	TCAGATGAACCAAAGGAAGTATGT
chr6_17076840	6	17076720	17076739	17076941	17076969	TGAACCTAGGAGGCTGAGGT	AGATTCTGGTACATTGTGTCTTTATTCT
chr6_40116264	6	40116143	40116164	40116366	40116388	TGTGTCATGGATCAAGGGTCTT	TCCTTCACCAAATTCTTCCCCG
chr6_62094287	6	62094166	62094187	62094388	62094411	GTTAGCCATTCTCTCTAGTGCC	TCTGCAACTCTACTGATAGTGAT

Abbreviations: Chr, chromosome; bp, base pair.

Supplementary Table 2. Estimated limit of detection of the single-cell DNA sequencing platform based on the dilution assay using cell line

The single-cell DNA sequencing data for RAJI and K562 mixture was downloaded from http://tapestriportal.com. RAJI and K562 were mixed with a ratio of 50:50, 99:1, 99.5:0.5, and 99.9:0.1. Three loci with known truth genotype that are distinct between these cell lines were used to determine the sensitivity of the single-cell DNA sequencing platform. The first table shows the known zygosity for the 3 loci for each cell line. In the second table, RAJI and K562 indicate RAJI- and K562-type genotypes as shown in the first table. The number and percentage of cells showing RAJI- and K562-type genotypes are shown for each mixed sample.

Het, heterozygous; WT, wildtype; Homo, homozygous.

**Variant**	**RAJI**	**K562**
TP53: chr17:7577581A>G	Het	WT
TP53: chr17:757815T>C	Het	Homo
TP53: chr17:7578211C>T	Het	WT

**Sample**	**RAJI**	**K562**
RAJI 50% + K562 50%	2404 (42.7%)	3358 (58.3%)
RAJI 99% + K562 1%	6502 (99.1%)	59 (0.9%)
RAJI 99.5% + K562 0.5%	3570 (99.4%)	21 (0.6%)
RAJI 99.9% + K562 0.1%	4519 (99.9%)	3 (0.1%)

Supplementary Table 3. List of 297 genes targeted by bulk next-generation sequencing.

**Gene name**									
ABCC9	*CALR*	*CUL5*	*FANCD2*	*HIST1H2BF*	*LEF1*	*MYCN*	*PIK3CG*	*SETBP1*	*TET2*
ABL1	*CARD11*	*CUX1*	*FANCE*	*HIST1H3D*	*LRP1B*	*MYD88*	*PIK3R1*	*SETD2*	*TGDS*
ACTG1	*CBL*	*CYLD*	*FANCG*	*HIST1H4D*	*LTB*	*NBN*	*PLA2G2D*	*SF3B1*	*TINF2 (TIN2)*
AKT1	*CBLB*	*DAXX*	*FANCI*	*HNRNPK*	*LUC7L2*	*NCOR1*	*PLCG2*	*SFRS1*	*TLR2*
ANKRD11	*CCND1*	*DCLRE1C*	*FANCL*	*HRAS*	*LYN*	*NCOR2*	*POT1*	*SFRS7*	*TLR9*
ARID1A	*CCND3*	*DDX3X*	*FAS*	*ICOS*	*MALT1*	*NF1*	*POU2AF1*	*SGK1*	*TNFAIP3*
ARID1B	*CD200*	*DIS3*	*FAT1*	*ID3*	*MAP2K1*	*NFE2*	*PRDM1*	*SH2B3*	*TNFRSF14*
ARID2	*CD274*	*DKC1*	*FAT3*	*IDH1*	*MAPK1*	*NFKB1*	*PRKCB*	*SHH*	*TNKS*
ARID5B	*CD58*	*DLC1*	*FBXW7*	*IDH2*	*MAX*	*NFKB2*	*PTEN*	*SMAD2*	*TOX*
ARPP21	*CD79A*	*DNM2*	*FGFR3*	*IKBKA*	*MDM2*	*NFKBIA*	*PTPN1*	*SMC1A*	*TP53*
ASXL1	*CD79B*	*DNMT1*	*FLI1*	*IKZF1*	*MED12*	*NFKBIE*	*PTPN11*	*SMC3*	*TRAF3*
ATF7IP	*CDK4*	*DNMT3A*	*FLT3*	*IKZF2*	*MEF2B*	*NOTCH1*	*RAD21*	*SMC5*	*TRAF6*
ATM	*CDKN2A*	*DNMT3B*	*FNDC3A*	*IKZF3*	*MEF2C*	*NOTCH2*	*RAD51C*	*SNX7*	*TYK2*
ATRX	*CDKN2B*	*EBF1*	*FOXP1*	*IL7R*	*MGA*	*NPM1*	*RAG1*	*SOCS1*	*TYK3*
B2M	*CDKN2C*	*ECT2L*	*FYN*	*IRAK1*	*miR125a*	*NR3C2*	*RAG2*	*SOX5*	*U2AF1*
BCL10	*CEBPA*	*EED*	*G6PC3*	*IRAK4*	*miR-142*	*NRAS*	*RASA2*	*SP140*	*U2AF2*
BCL2	*CEBPE*	*EGR1*	*GAB2*	*IRF1*	*mIR155*	*NSD2*	*RB1*	*SPEN*	*UBR5*
BCL6	*CHD2*	*EGR2*	*GATA1*	*IRF4*	*mIR15a*	*NT5C2*	*REL*	*SPIB*	*USP29*
BCL7A	*CHK2*	*ELANE*	*GATA2*	*IRF7*	*mIR16-1*	*PAG1*	*RELA*	*SRSF2*	*VPREB1*
BCOR	*CIITA*	*EP300*	*GATA3*	*ITPKB*	*MIR17HG*	*PALB2*	*RELB*	*STAG1*	*WHSC1*
BCR	*CNOT3*	*EPHA7*	*GCET2*	*JAK1*	*mIR21*	*PAX5*	*RELN*	*STAG2*	*WHSC1L1*
BIRC3	*CREBBP*	*EPOR*	*GFI1B*	*JAK2*	*mir34b*	*PDCD1*	*RHOA*	*STAT1*	*WT1*
BLK	*CRLF2*	*ERG*	*GNA13*	*JAK3*	*mir34c*	*PDCD1LG2*	*RIPK1*	*STAT3*	*XPO1*
BMI1	*CSF2RA*	*ETV6*	*GNAS*	*JARID2*	*MLL*	*PDGFRB*	*ROBO1*	*SUZ12*	*ZAP70*
BRAF	*CSF3R*	*EZH2*	*GNB1*	*KDM4C*	*MLL2*	*PEG3*	*ROR1*	*SYK*	*ZMYM2*
BRIP1	*CTBP1*	*FAM46C*	*GPRC5A*	*KDM6A*	*MLL3*	*PHF6*	*RPL10*	*TBL1XR1*	*ZMYM3*
BTG1	*CTBP2*	*FAM5C*	*HAX1*	*KIT*	*MPL*	*PHIP*	*RPL5*	*TCF3*	*ZRSR2*
BTK	*CTCF*	*FANCA*	*HIST1H1E*	*KLHL6*	*MS4A1*	*PIGA*	*RUNX1*	*TERC*	
BTLA	*CTLA4*	*FANCB*	*HIST1H2AD*	*KRAS*	*MYB*	*PIK3CA*	*RUNX2*	*TERT*	
C22orf194	*CTNNA1*	*FANCC*	*HIST1H2BE*	*LAMB4*	*MYC*	*PIK3CB*	*SAMHD1*	*TET1*	

Supplementary Table 4. List of probes/primers used for ddPCR assay

primer/probe ID	sequence and chromosome coordinates of the assay's amplicon	Variant
dHsaMDS708288136	hg19|chr20:31023047-31023169:+<br>CCCCAGTTCCACACCTGAATCCTCACCGACTGATTGCCTGCAG<br>AACAGAGCATTTGATGAC[G/A]AATTAGGGCTTGGTGGCTCAT<br>GCCCTCCTATGAGGGAAAGTGATACTAGACAAGAAAACTT	*ASXL1*:exon13:c.G2593A:p.E865K
dHsaMDS299498199	hg19|chr13:28602279-28602401:-<br>AGGCACTCATGTCAGAACTCAAGATGATGACCCAGCTGGGAA<br>GCCACGAGAATATTGTGAA[C/A]CTGCTGGGGGCGTGCACAC<br>TGTCAGGTAACCCACTTCCACGAAAATCACCTCATCAAAAAG	*FLT3*:exon16:c.C2028A:p.N676K
dHsaMDS887479234	hg19|chr13:28602268-28602390:-<br>TCAGAACTCAAGATGATGACCCAGCTGGGAAGCCACGAGAAT<br>ATTGTGAACCTGCTGGGGG[C/T]GTGCACACTGTCAGGTAACC<br>CACTTCCACGAAAATCACCTCATCAAAAAGACTGTAGCTTG	*FLT3*:exon16:c.C2039T:p.A680V
dHsaMDS600877565	hg19|chr13:28592568-28592690:-<br>GTCACCCACGGGAAAGTGGTGAAGATATGTGACTTTGGATTG<br>GCTCGAGATATCATGAGTG[A/G]TTCCAACTATGTTGTCAGGG<br>GCAATGTGAGGCTGCTATTTCCTACTTATTTTTATACGGCT	*FLT3*:exon20:c.A2516G:p.D839G
dHsaMDV2010053	hg19|chr2:209113052-209113174:-<br>CATTATCTGCAAAAATATCCCCCGGCTTGTGAGTGGATGGGTA<br>AAACCTATCATCATAGGT[C/T]GTCATGCTTATGGGGATCAAG<br>TAAGTCATGTTGGCAATAATGTGATTTTGCATGTTTTTTT	*IDH1*:exon4:c.C394T:p.R132C
dHsaMDV2010055	hg19|chr2:209113051-209113173:-<br>ATTATCTGCAAAAATATCCCCCGGCTTGTGAGTGGATGGGTAA<br>AACCTATCATCATAGGTC[G/A]TCATGCTTATGGGGATCAAGT<br>AAGTCATGTTGGCAATAATGTGATTTTGCATGTTTTTTTT	*IDH1*:exon4:c.G395A:p.R132H
dHsaMDV2010057	hg19|chr15:90631873-90631995:-<br>ATCTCTGTCCTCACAGAGTTCAAGCTGAAGAAGATGTGGAAA<br>AGTCCCAATGGAACTATCC[G/A]GAACATCCTGGGGGGGACT<br>GTCTTCCGGGAGCCCATCATCTGCAAAAACATCCCACGCCTA	*IDH2*:exon4:c.G419A:p.R140Q
dHsaMDV2510596	hg19|chr12:25398223-25398345:-<br>TTATTTTTATTATAAGGCCTGCTGAAAATGACTGAATATAAACT<br>TGTGGTAGTTGGAGCTG[G/A]TGGCGTAGGCAAGAGTGCCTT<br>GACGATACAGCTAATTCAGAATCATTTTGTGGACGAATAT	*KRAS*:exon2:c.G35A:p.G12D

dHsaMDV2510586	hg19|chr12:25398223-25398345:- TTATTTTTATTATAAGGCCTGCTGAAAATGACTGAATATAAACT TGTGGTAGTTGGAGCTG[G/C]TGGCGTAGGCAAGAGTGCCTT GACGATACAGCTAATTCAGAATCATTTTGTGGACGAATAT	*KRAS*:exon2:c.G35C:p.G12A
dHsaMDS589521946	hg19|chr12:25380218-25380340:- TACAGGAAGCAAGTAGTAATTGATGGAGAAACCTGTCTCTTG GATATTCTCGACACAGCAG[G/T]TCAAGAGGAGTACAGTGCA ATGAGGGACCAGTACATGAGGACTGGGGAGGGCTTTCTTTGT	*KRAS*:exon3:c.G179T:p.G60V
dHsaMDS890511303	hg19|chr5:170837482-170837604:+ TATGAAGTGTTGTGGTTCCTTAACCACATTTCTTTTTTTTTTTTT CCAGGCTATTCAAGAT[C/CTCTG]TCTGGCAGTGGAGGAAGTC TCTTTAAGAAAATAGTTTAAACAATTTGTTAAAAAATTTTCC	*NPM1*:exon11:c.859_860insTCTG: p.L287fs
dHsaMDV2010093	hg19|chr1:115258687-115258809:- GTTTCCAACAGGTTCTTGCTGGTGTGAAATGACTGAGTACAAA CTGGTGGTGGTTGGAGCA[G/A]GTGGTGTTGGGAAAAGCGC ACTGACAATCCAGCTAATCCAGAACCACTTTGTAGATGAATA	*NRAS*:exon2:c.G34A:p.G12S
dHsaMDV2010095	hg19|chr1:115258686-115258808:- TTTCCAACAGGTTCTTGCTGGTGTGAAATGACTGAGTACAAAC TGGTGGTGGTTGGAGCAG[G/A]TGGTGTTGGGAAAAGCGCA CTGACAATCCAGCTAATCCAGAACCACTTTGTAGATGAATAT	*NRAS*:exon2:c.G35A:p.G12D
dHsaMDS721630771	hg19|chr1:115258686-115258808:- TTTCCAACAGGTTCTTGCTGGTGTGAAATGACTGAGTACAAAC TGGTGGTGGTTGGAGCAG[G/C]TGGTGTTGGGAAAAGCGCA CTGACAATCCAGCTAATCCAGAACCACTTTGTAGATGAATAT	*NRAS*:exon2:c.G35C:p.G12A
dHsaMDV2510528	hg19|chr1:115258686-115258808:- TTTCCAACAGGTTCTTGCTGGTGTGAAATGACTGAGTACAAAC TGGTGGTGGTTGGAGCAG[G/T]TGGTGTTGGGAAAAGCGCAC TGACAATCCAGCTAATCCAGAACCACTTTGTAGATGAATAT	*NRAS*:exon2:c.G35T:p.G12V
dHsaMDV2510534	hg19|chr1:115258684-115258806:- TCCAACAGGTTCTTGCTGGTGTGAAATGACTGAGTACAAACTG GTGGTGGTTGGAGCAGGT[G/C]GTGTTGGGAAAAGCGCACT GACAATCCAGCTAATCCAGAACCACTTTGTAGATGAATATGA	*NRAS*:exon2:c.G37C:p.G13R
dHsaMDV2510526	hg19|chr1:115258683-115258805:- CCAACAGGTTCTTGCTGGTGTGAAATGACTGAGTACAAACTG GTGGTGGTTGGAGCAGGTG[G/A]TGTTGGGAAAAGCGCACT GACAATCCAGCTAATCCAGAACCACTTTGTAGATGAATATGAT	*NRAS*:exon2:c.G38A:p.G13D

<table>
<tr><td>dHsaMDV2510524</td><td>hg19|chr1:115258683-115258805:-
CCAACAGGTTCTTGCTGGTGTGAAATGACTGAGTACAAACTG
GTGGTGGTTGGAGCAGGTG[G/T]TGTTGGGAAAAGCGCACTG
ACAATCCAGCTAATCCAGAACCACTTTGTAGATGAATATGAT</td><td>NRAS:exon2:c.G38T:p.G13V</td></tr>
<tr><td>dHsaMDS381711539</td><td>hg19|chr12:112888105-112888227:+
CCAATGGACTATTTTAGAAGAAATGGAGCTGTCACCCACATCA
AGATTCAGAACACTGGTG[A/C]TTACTATGACCTGTATGGAGG
GGAGAAATTTGCCACTTTGGCTGAGTTGGTCCAGTATTAC</td><td>PTPN11:exon3:c.A182C:p.D61A</td></tr>
<tr><td>dHsaMDS585736928</td><td>hg19|chr12:112888078-112888200:+
TCTTTATTTGTCCCCTTGCCTCCCTTTCCAATGGACTATTTTAGA
AGAAATGGAGCTGTCA[C/A]CCACATCAAGATTCAGAACACTG
GTGATTACTATGACCTGTATGGAGGGGAGAAATTTGCC</td><td>PTPN11:exon3:c.C155A:p.T52N</td></tr>
<tr><td>dHsaMDS797586326</td><td>hg19|chr21:36252879-36253001:-
CTAGGGGATGTTCCAGATGGCACTCTGGTCACTGTGATGGCT
GGCAATGATGAAAACTACT[C/T]GGCTGAGCTGAGAAATGCT
ACCGCAGCCATGAAGAACCAGGTTGCAAGATTTAATGACCTC</td><td>RUNX1:exon5:c.C422T:p.S141L</td></tr>
<tr><td>dHsaMDS704617272</td><td>hg19|chr21:36171553-36171675:+
TCCACCCCAGCTCAGCTGCAAAGAATGTGTTTTCAAGTGGCTT
ACTTGAGAGTCGACTGGA[A/AAGG]AGTTCTGCAGAGAGGGT
TGTCATGCCGCTGGCACGTCCAGGTGAAATGGGCGTTGCTGG
GT</td><td>RUNX1:exon8:c.950_951insCCT:
p.L317delinsLL</td></tr>
<tr><td>dHsaMDS837236043</td><td>hg19|chr21:36171552-36171674:+
TTCCACCCCAGCTCAGCTGCAAAGAATGTGTTTTCAAGTGGCT
TACTTGAGAGTCGACTGG[A/ACC]AAGTTCTGCAGAGAGGGT
TGTCATGCCGCTGGCACGTCCAGGTGAAATGGGCGTTGCTGG
G</td><td>RUNX1:exon8:c.951_952insGG:p.S318fs</td></tr>
<tr><td>dHsaMDS911558603</td><td>hg19|chr17:7578355-7578477:+
AGCCCCAGCTGCTCACCATCGCTATCTGAGCAGCGCTCATGGT
GGGGGCAGCGCCTCACAA[C/-]CTCCGTCATGTGCTGTGACTG
CTTGTAGATGGCCATGGCGCGGACGCGGGTGCCGGGCGGG</td><td>TP53:exon5:c.514delG:p.V172fs</td></tr>
<tr><td>dHsaMDS234249306</td><td>hg19|chr17:7578229-7578351:+
TCCAAATACTCCACACGCAAATTTCCTTCCACTCGGATAAGATG
CTGAGGAGGGGCCAGAC[C/T]TAAGAGCAATCAGTGAGGAAT
CAGAGGCCTGGGGACCCTGGGCAACCAGCCCTGTCGTCTC</td><td>TP53:splicing (chr17: 7,578,290 C>T)</td></tr>
<tr><td>dHsaMDS590501846</td><td>hg19|chr11:32417846-32417968:+
ATATCTCTTATTGCAGCCTGGGTAAGCACACATGAAGGGGCG
TTTCTCACTGGTCTCAGAT[G/GCCGA]CCGACCGTACAAGAGT
CGGGGCTACTCCAGGCACACGTCGCACATCCTGCAGGCAGAG
AGT</td><td>WT1:exon7:c.1144_1145insTCGG:
p.A382fs</td></tr>
</table>

Supplementary Fig. 1a

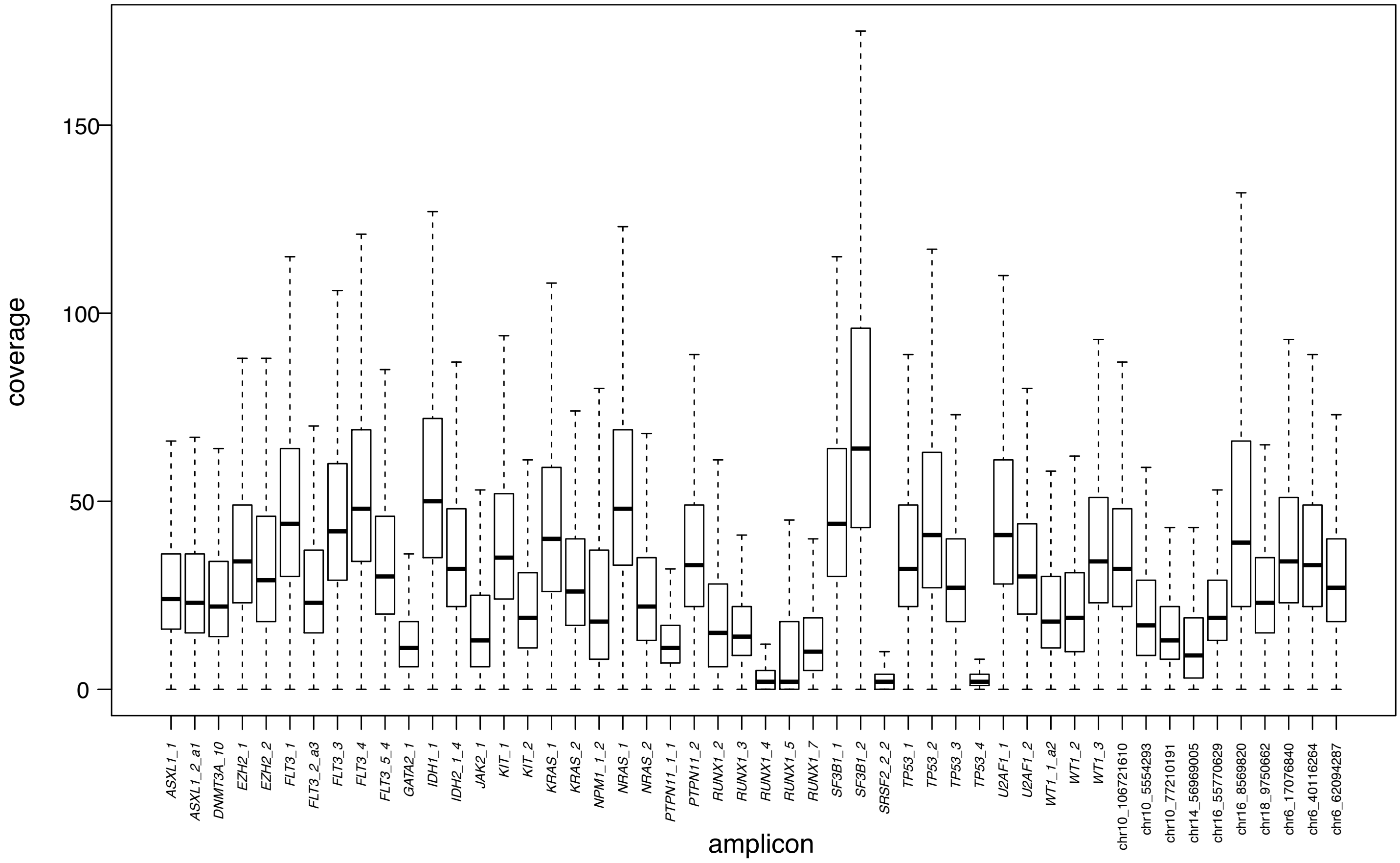

Supplementary Fig. 1b

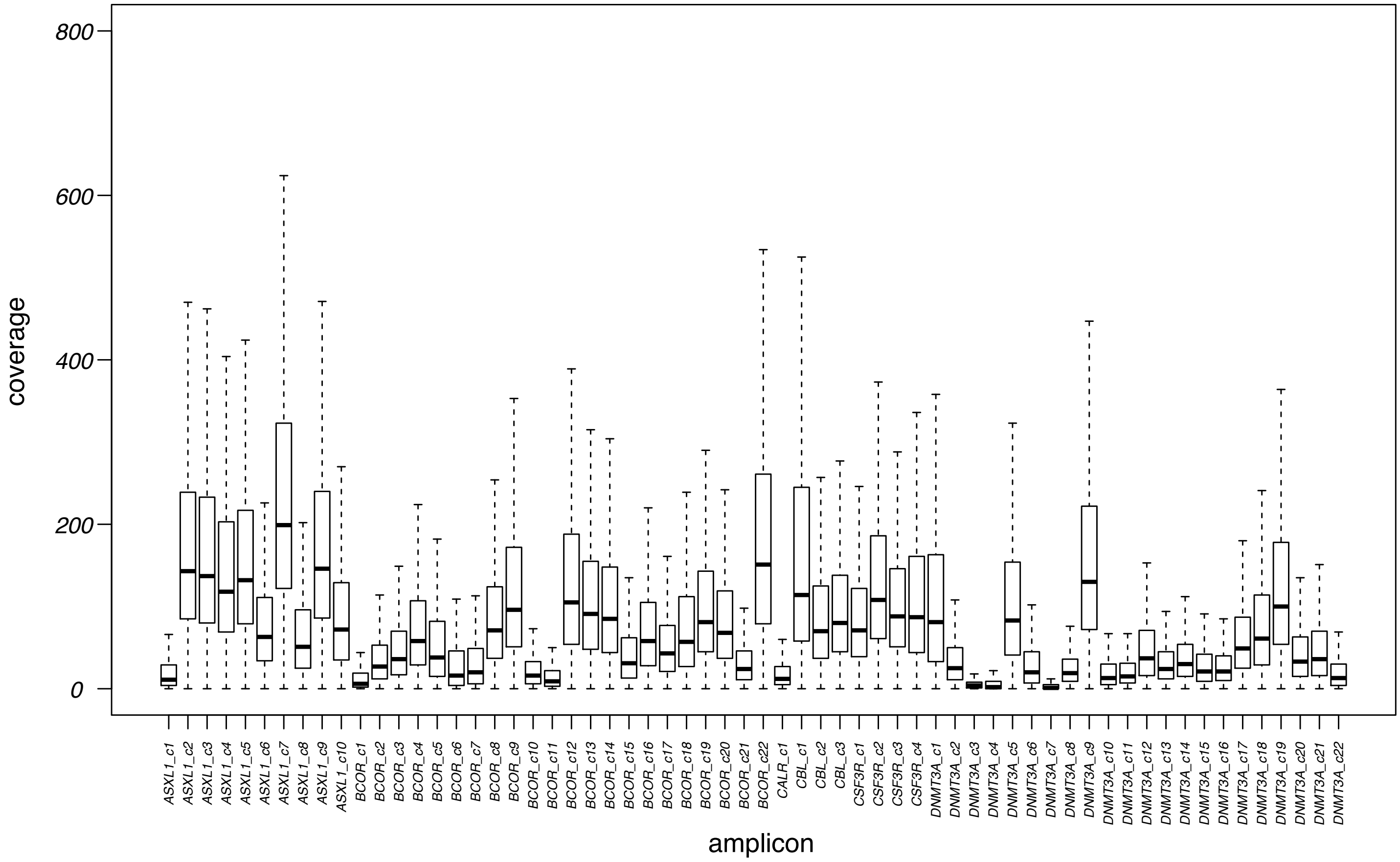

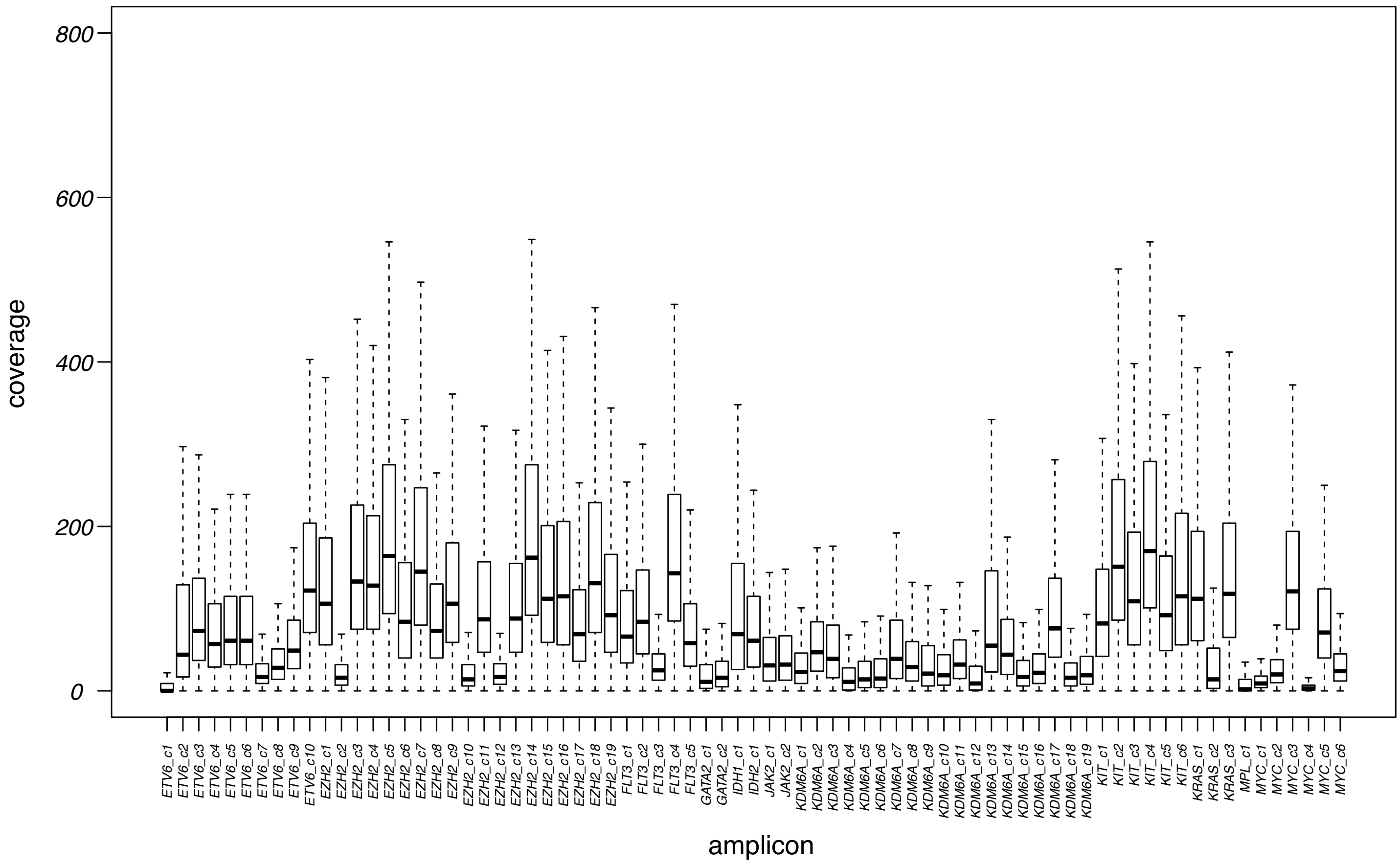

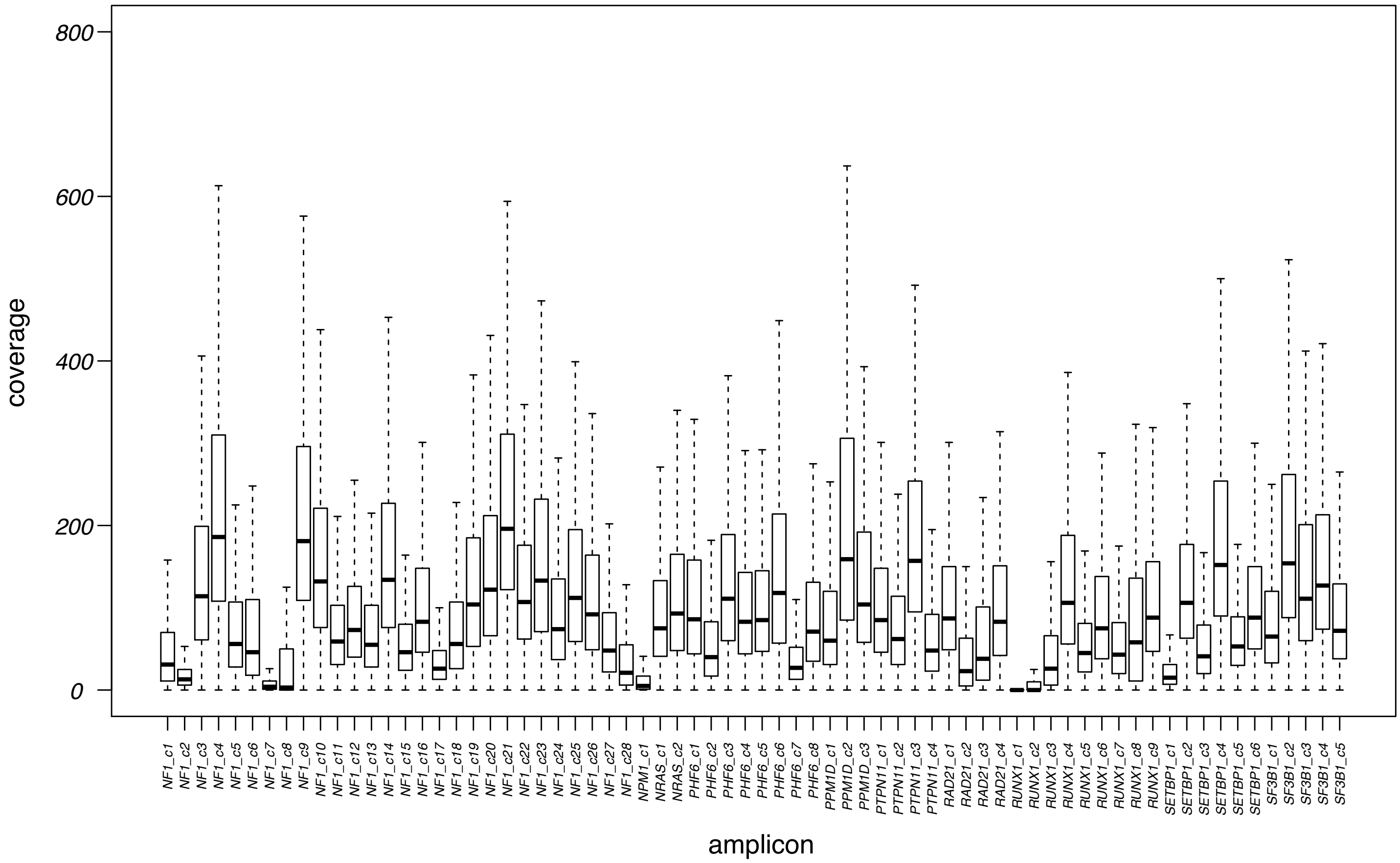

coverage
800
600
400
200
0
amplicon
NF1_c1
NF1_c2
NF1_c3
NF1_c4
NF1_c5
NF1_c6
NF1_c7
NF1_c8
NF1_c9
NF1_c10
NF1_c11
NF1_c12
NF1_c13
NF1_c14
NF1_c15
NF1_c16
NF1_c17
NF1_c18
NF1_c19
NF1_c20
NF1_c21
NF1_c22
NF1_c23
NF1_c24
NF1_c25
NF1_c26
NF1_c27
NF1_c28
NPM1_c1
NRAS_c1
NRAS_c2
PHF6_c1
PHF6_c2
PHF6_c3
PHF6_c4
PHF6_c5
PHF6_c6
PHF6_c7
PHF6_c8
PPM1D_c1
PPM1D_c2
PPM1D_c3
PTPN11_c1
PTPN11_c2
PTPN11_c3
PTPN11_c4
RAD21_c1
RAD21_c2
RAD21_c3
RAD21_c4
RUNX1_c1
RUNX1_c2
RUNX1_c3
RUNX1_c4
RUNX1_c5
RUNX1_c6
RUNX1_c7
RUNX1_c8
RUNX1_c9
SETBP1_c1
SETBP1_c2
SETBP1_c3
SETBP1_c4
SETBP1_c5
SETBP1_c6
SF3B1_c1
SF3B1_c2
SF3B1_c3
SF3B1_c4
SF3B1_c5

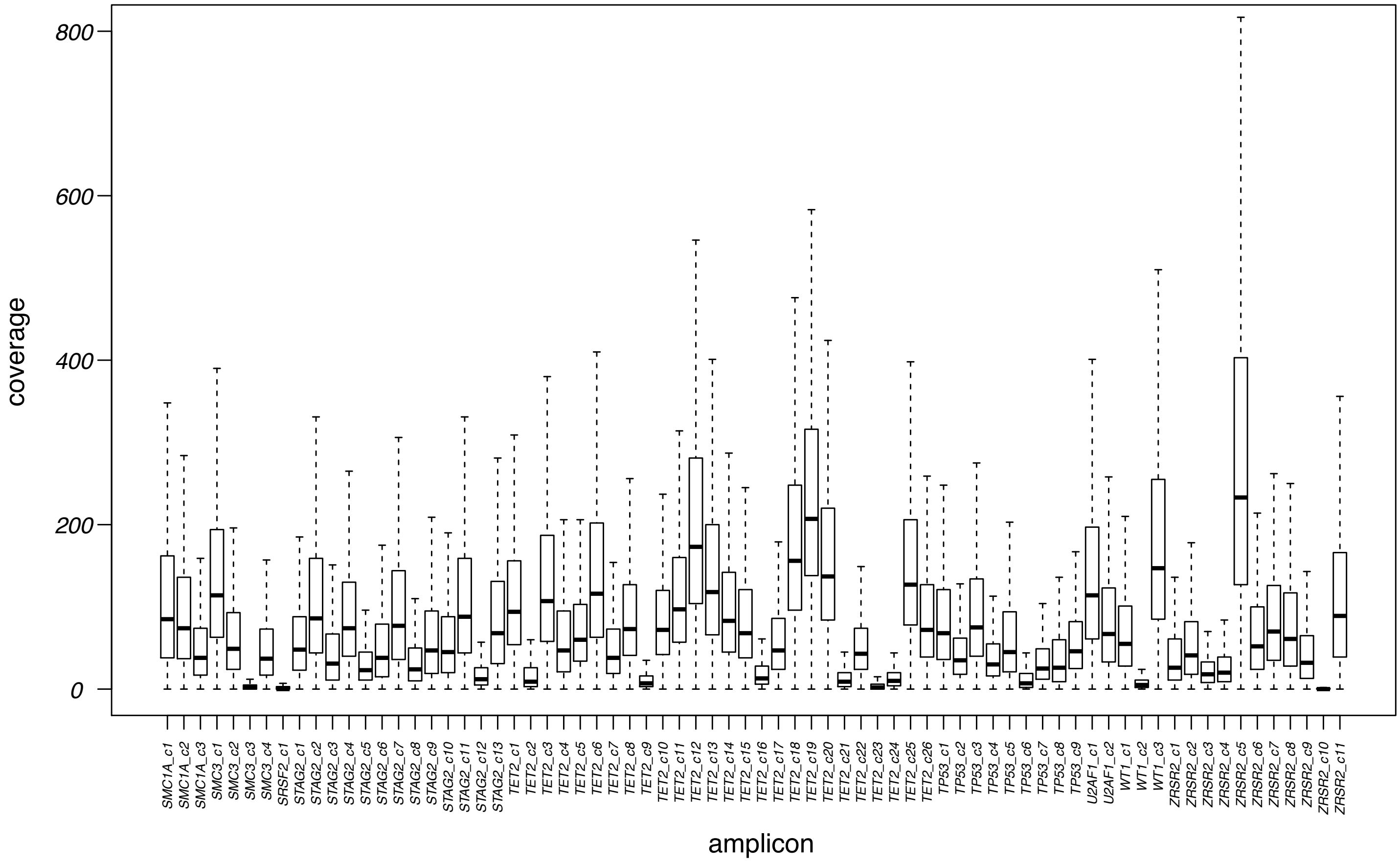

coverage
800
600
400
200
0
amplicon
SMC1A_c1
SMC1A_c2
SMC1A_c3
SMC3_c1
SMC3_c2
SMC3_c3
SMC3_c4
SRSF2_c1
STAG2_c1
STAG2_c2
STAG2_c3
STAG2_c4
STAG2_c5
STAG2_c6
STAG2_c7
STAG2_c8
STAG2_c9
STAG2_c10
STAG2_c11
STAG2_c12
STAG2_c13
TET2_c1
TET2_c2
TET2_c3
TET2_c4
TET2_c5
TET2_c6
TET2_c7
TET2_c8
TET2_c9
TET2_c10
TET2_c11
TET2_c12
TET2_c13
TET2_c14
TET2_c15
TET2_c16
TET2_c17
TET2_c18
TET2_c19
TET2_c20
TET2_c21
TET2_c22
TET2_c23
TET2_c24
TET2_c25
TET2_c26
TP53_c1
TP53_c2
TP53_c3
TP53_c4
TP53_c5
TP53_c6
TP53_c7
TP53_c8
TP53_c9
U2AF1_c1
U2AF1_c2
WT1_c1
WT1_c2
WT1_c3
ZRSR2_c1
ZRSR2_c2
ZRSR2_c3
ZRSR2_c4
ZRSR2_c5
ZRSR2_c6
ZRSR2_c7
ZRSR2_c8
ZRSR2_c9
ZRSR2_c10
ZRSR2_c11

Supplementary Fig. 1. Distribution of coverage per cell per amplicon in all sequenced cells. **(a)** A total 69 patients' samples sequenced by pre-designed 19-gene panel. The first 40 amplicons cover the hotspots of 19 AML-associated genes. The remaining 10 amplicons cover commonly heterozygous SNP loci. N=69 patients that were sequenced by pre-designed 19-gene panel. **(b)** A total 54 patients' samples sequenced by custom-designed panel. The 279 amplicons cover the hotspots of 37 cancer genes. N=54 patients that were sequenced by custom-designed panel. The amplicons are shown on the X axis. The Y axis represents the coverage for each sequenced cell. The thick line within each box represents the median, and the top and bottom edges of the box represent the 25th and 75th percentiles, respectively. The upper and lower whiskers represent the 75th percentile plus 1.5 times the interquartile range and the 25th percentile minus 1.5 times the interquartile range, respectively. Data points that fell outside of the upper and lower whiskers were considered outliers and are not shown.

Supplementary Fig. 2

(a)

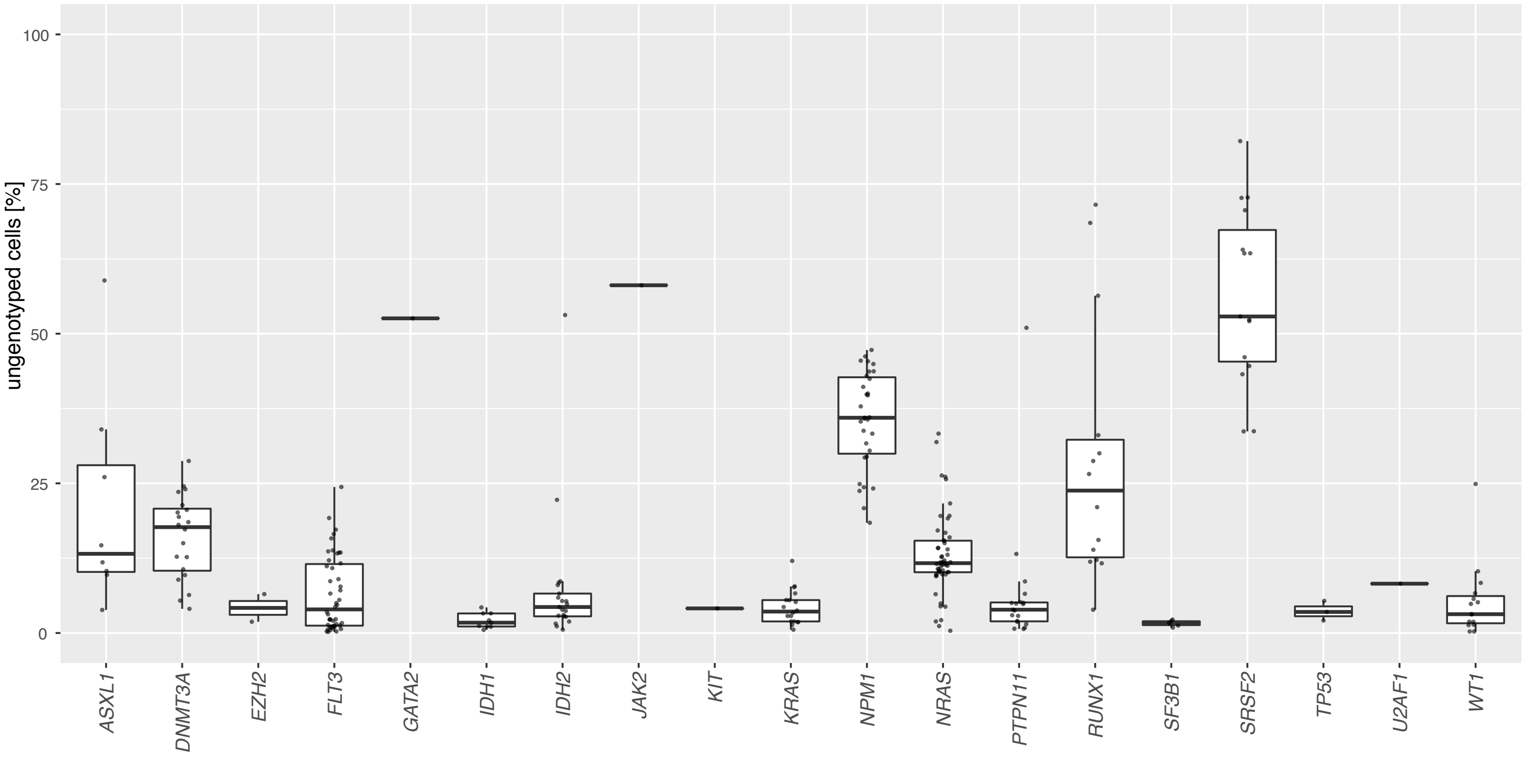

(b)

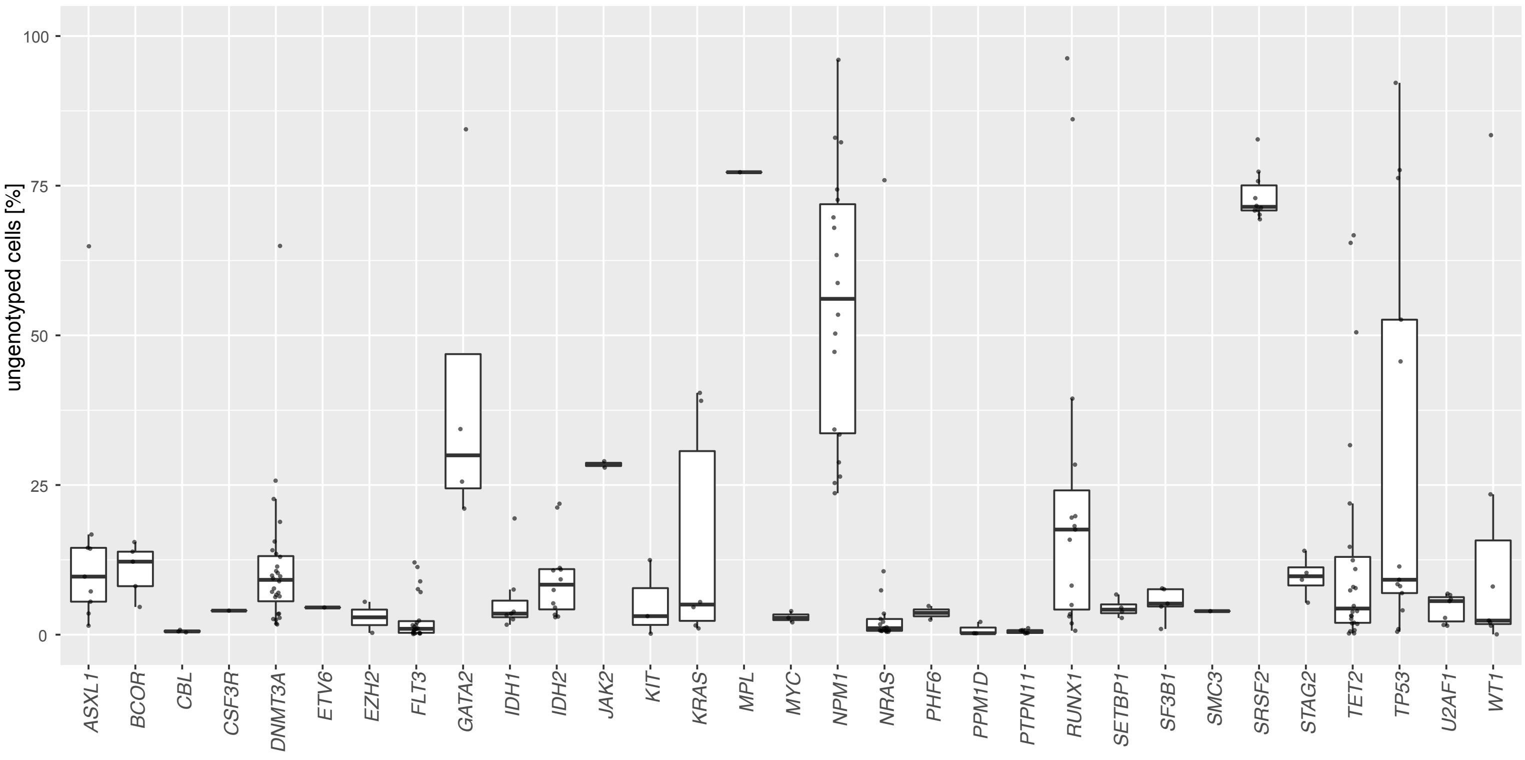

Supplementary Fig. 2. Distribution of the percentage of ungenotyped cells for each variant based on the genes. **(a)** A total 69 patients' samples sequenced by 19-gene panel. The 19 AML-associated genes covered by the panel are shown on the X axis. N=69 patients that were sequenced by pre-designed 19-gene panel. **(b)** A total 54 patients' samples sequenced by custom-designed panel. Among the 37 genes covered by the panel, 31 genes in which at least one mutation was detected among the 54 patients are shown on the X axis. N=54 patients that were sequenced by custom-designed panel.The Y axis represents the percentage of ungenotyped cells for each variant grouped by the mutated genes. The percentage of ungenotyped cells were calculated for each variant from each sample as follows: (number of genotyped cells [wildtype, heterozygous, or homozygous]) / (number of total sequenced cells) × 100. The thick line within each box represents the median, and the top and bottom edges of the box represent the 25th and 75th percentiles, respectively. The upper and lower whiskers represent the 75th percentile plus 1.5 times the interquartile range and the 25th percentile minus 1.5 times the interquartile range, respectively. All data points are shown with dots.

Supplementary Fig. 3

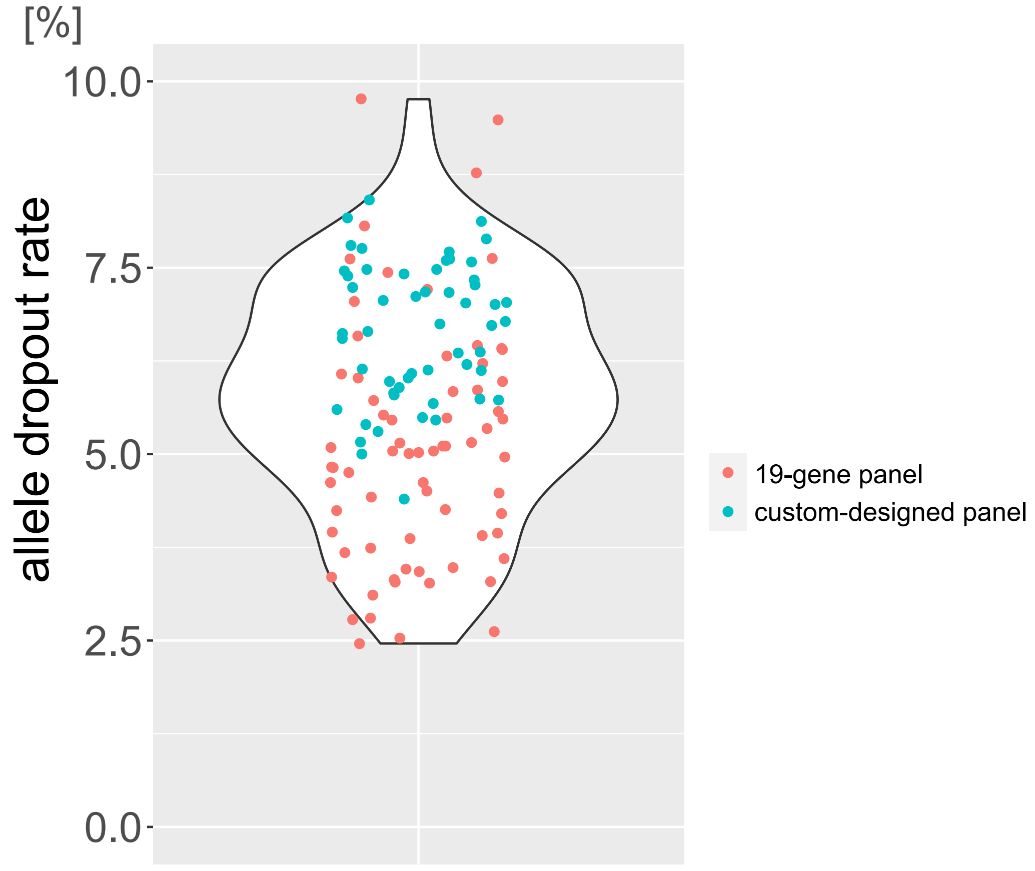

Supplementary Fig. 3. Violin plot with jittered points showing the distribution of the allele dropout (ADO) rate. The Y axis shows the ADO rate from unique patients. Red and green points represent the samples sequenced by 19-gene panel (N=69) and custom-designed panel (N=54), respectively.

Supplementary Fig. 4

KRAS exon2:c.G35A:p.G12D
(mutated in 67 of 7053 [0.9%] cells sequenced)
bulk NGS VAF :undetectable

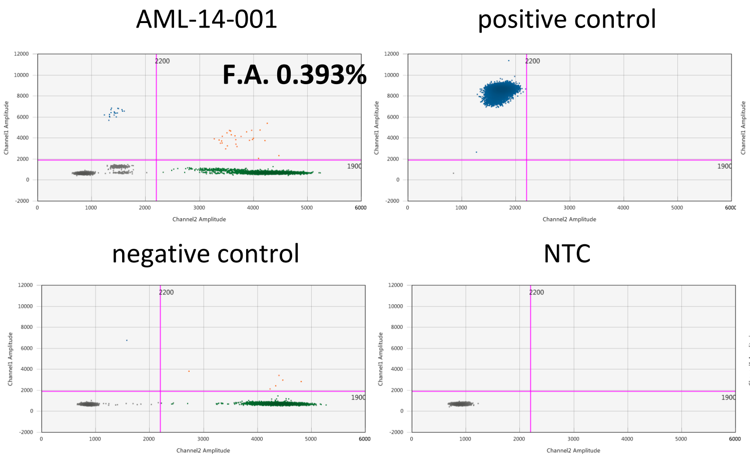

IDH2 exon4:c.G419A:p.R140Q
(mutated in 56 of 9864 [0.6%] cells sequenced)
bulk NGS VAF: undetectable

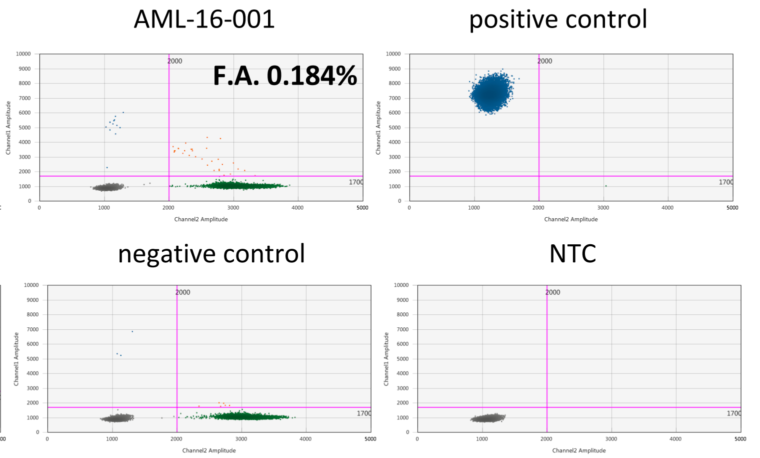

NRAS exon2:c.G35A:p.G12D
(mutated in 42 of 8219 [0.5%] cells sequenced)
bulk NGS VAF: undetectable

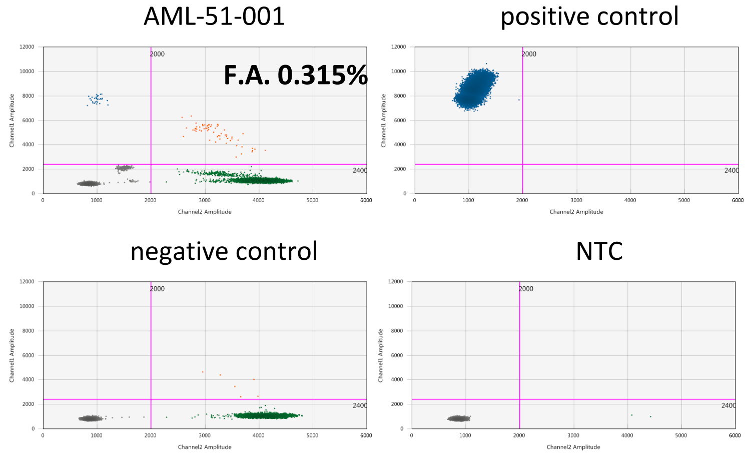

NRAS exon2:c.G38T:p.G13V
(mutated in 10 of 2252 [0.4%] cells sequenced)
bulk NGS VAF: undetectable

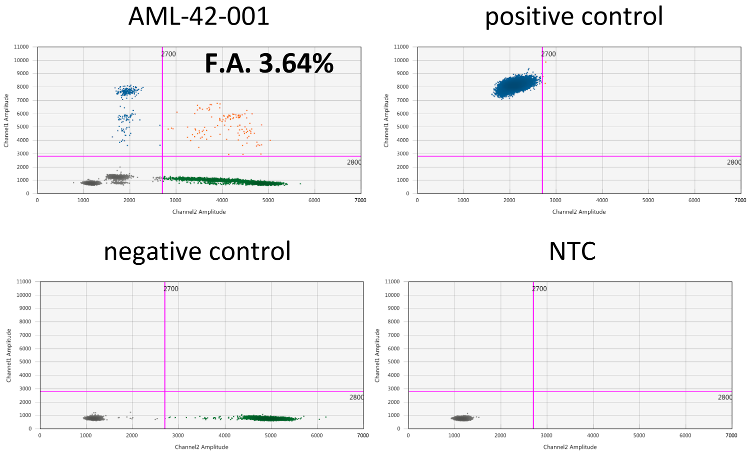

NRAS exon2:c.G38A:p.G13D
(mutated in 32 of 11398 [0.3%] cells sequenced)
bulk NGS VAF: undetectable

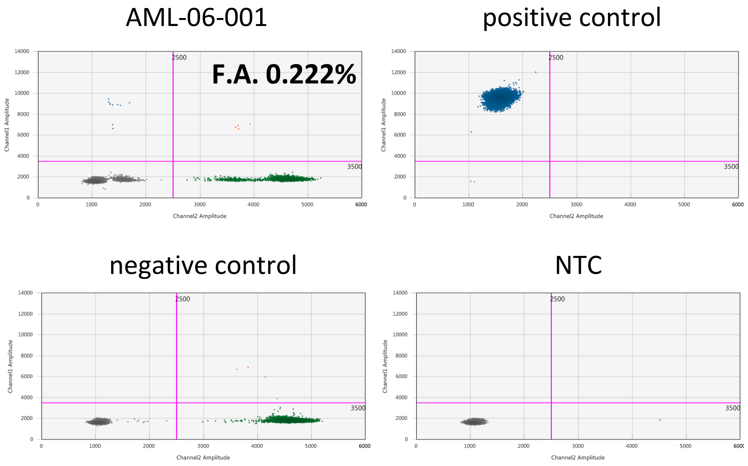

NRAS exon2:c.G35A:p.G12D
(mutated in 6 of 4359 [0.1%] cells sequenced)
bulk NGS VAF: undetectable

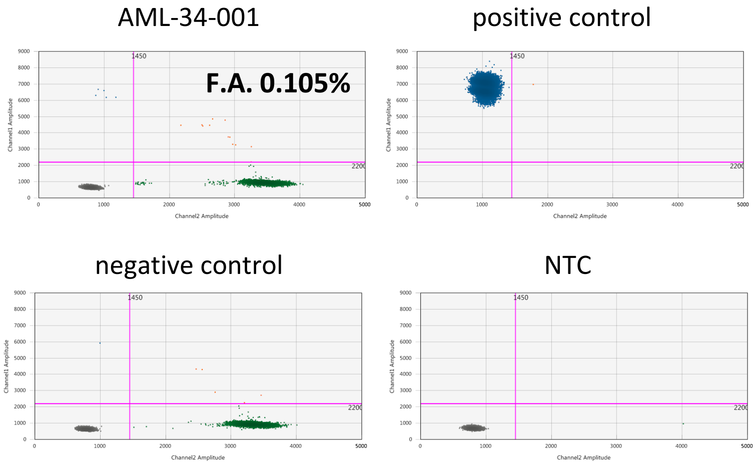

Supplementary Fig. 4. Validation of single-cell DNA sequencing-specific variants by ddPCR. Representative cases of single-cell DNA sequencing-specific variants that were undetectable by bulk next generation sequencing but were validated by droplet digital PCR (ddPCR). 2D plots for ddPCR results are shown for the indicated sample (upper left), a positive control using synthesized DNA (upper right), a negative control using wild-type human DNA (lower left), and a no-template control without DNA (lower right). Within each 2D plot, the blue cluster in the upper left quadrant represents droplets with mutated DNA only, and the orange cluster in the upper right quadrant represents droplets with both mutated and wild-type DNA. The green cluster in the lower right quadrant represents droplets with wild-type DNA only, and the grey cluster in the lower left quadrant represents droplets without DNA from the targeted locus. Fractional abundance (F.A.) was calculated as follows: (number of droplets with mutated DNA) / (number of droplets with mutated DNA + number of droplets with wild-type DNA) × 100.

The smallest mutations that were validated by ddPCR assay was mutated in 0.1% of the total sequenced cells. Therefore, along with the cell line data shown in Supplemental Table 4, the limit of detection of the platform was estimated as 0.1%.

NGS, next-generation sequencing; VAF, variant allele fraction; NTC, no-template control.

Supplementary Fig. 5

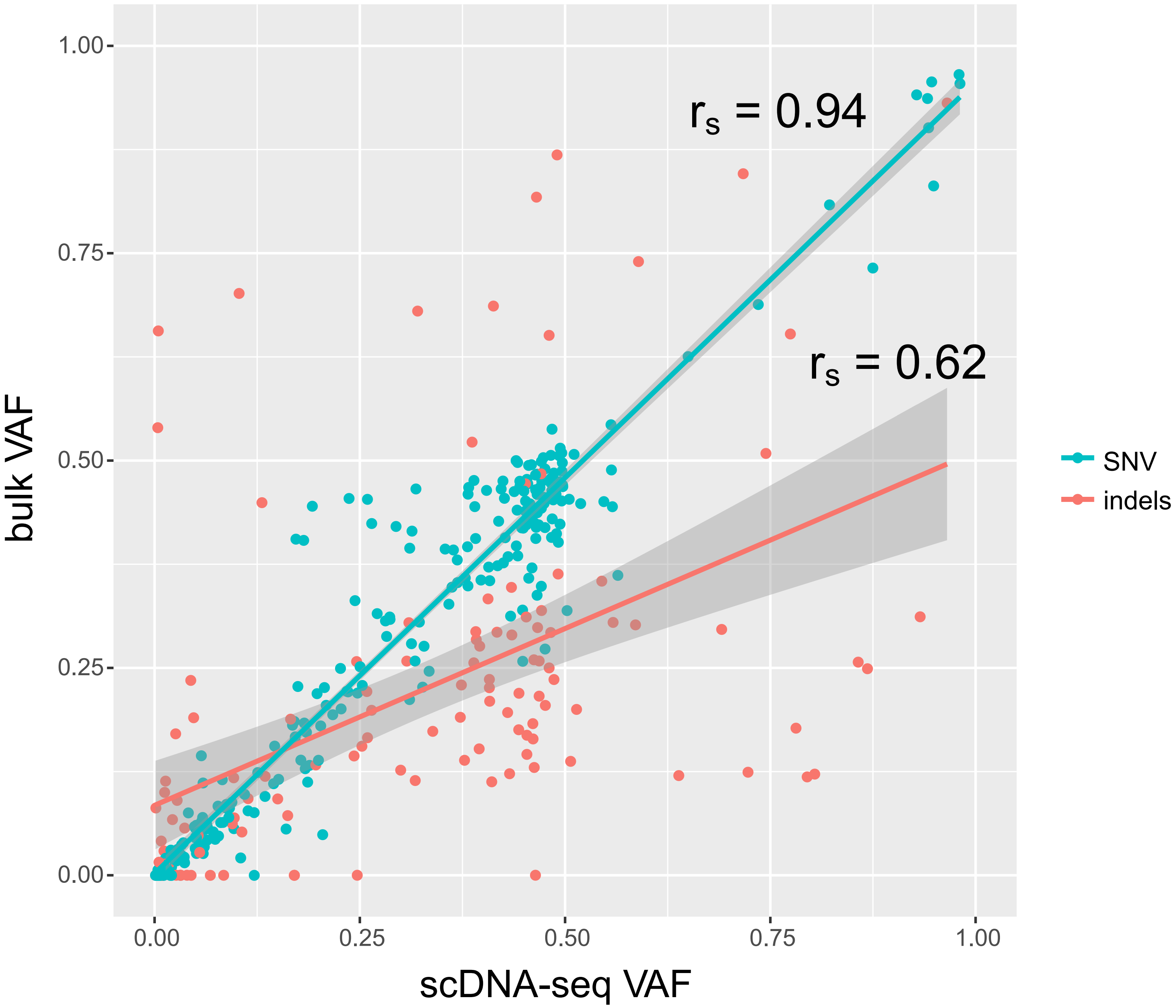

Supplementary Fig. 5. Correlation of bulk-sequencing VAF and VAF inferred from single-cell genotype data. The X-axis shows the VAF from the single-cell genotype data (scDNA-seq VAF). The Y-axis shows the VAF from the bulk next-generation sequencing (bulk VAF). Green dots represent single-nucleotide variants (SNV), and red dots represent insertion/deletion variants (indels). The linear regression lines were added to best fit the distribution of the dots. The shaded areas around the lines represent the 95% confidence intervals. N=418 mutations (295 as SNV and 123 as indels) with available bulk VAF data. scDNA-seq VAF and bulk VAF matched well for SNV ($r_s = 0.94$, $p < 2.2e-16$), whereas the concordance was weaker for indels ($r_s = 0.62$, $p = 2.13e-14$). Two-sided Spearman's rank correlation was used without adjustment for multiple comparisons.

Supplementary Fig. 6a

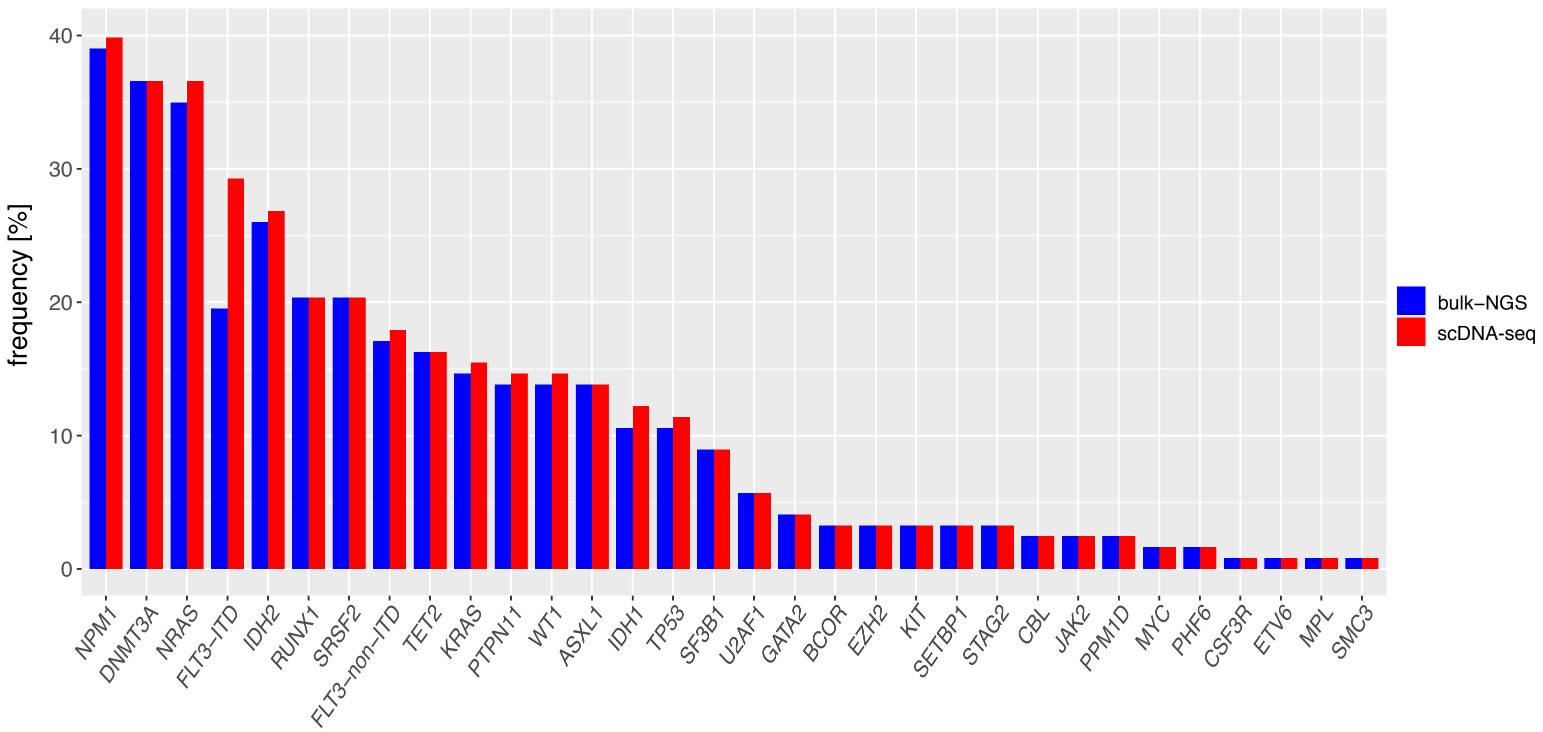

Supplementary Fig. 6 (a) Frequency of driver mutations based on bulk and single-cell DNA sequencing. The X axis represents the gene. The Y axis shows the frequency of patients harboring at least one mutation within each gene. Blue bars represent the frequency based on the bulk next generation sequencing (bulk-NGS) data that was used for orthogonal validation, and red bars represent the frequency based on the single-cell DNA sequencing (scDNA-seq) data. N=123 patients.

Supplementary Fig. 6b

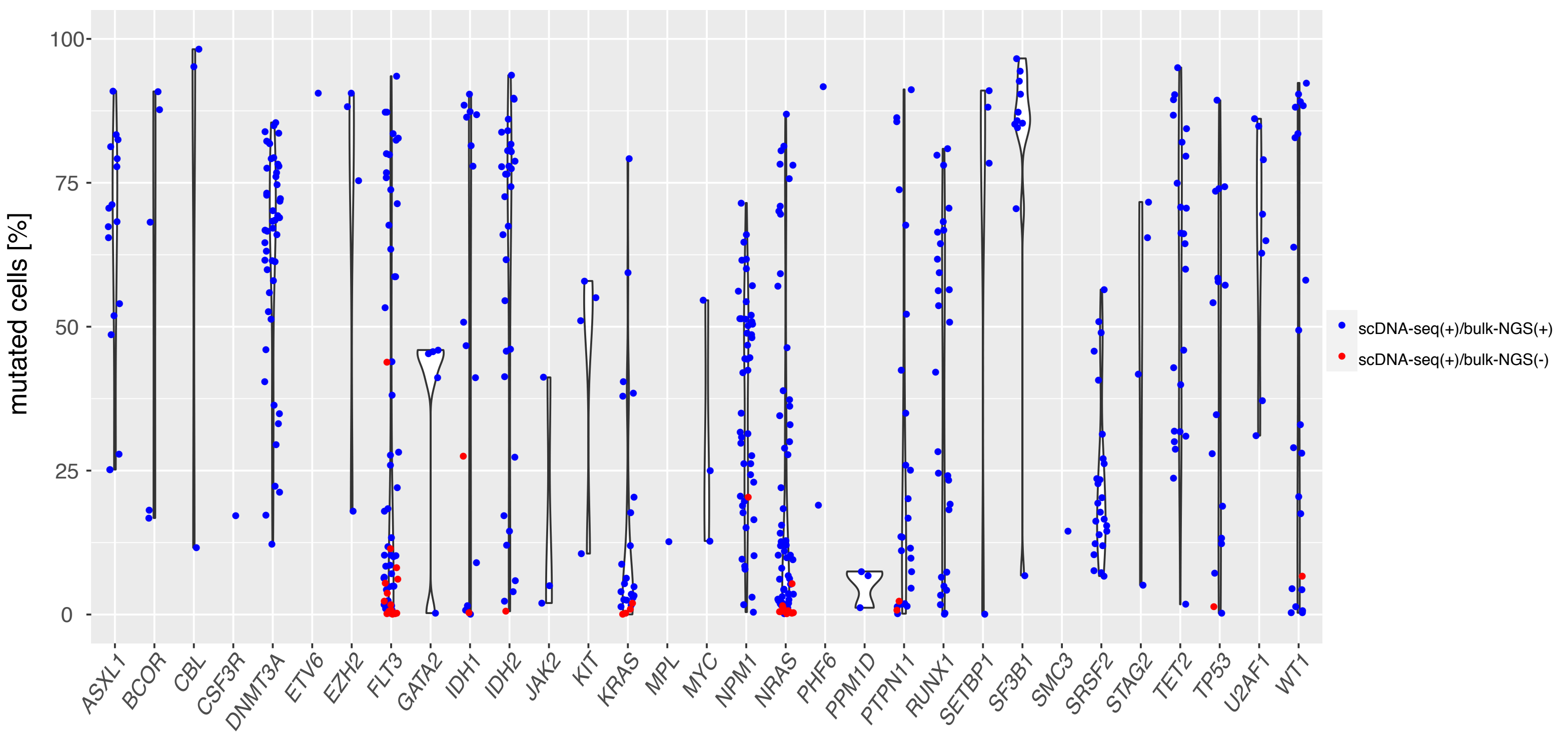

Supplementary Fig. 6 (b) Percentage of mutated cells for each driver mutation. The X axis represents the gene. The Y axis shows the percentage of mutated cells for each variant detected by scDNA-seq. Blue plots represent the variants that were detected by both scDNA-seq and bulk-NGS. Red plots represent the variants that were detected by scDNA-seq but were undetectable by bulk-NGS. Percentage of mutated cells was calculated as follows: (number of cells that were single-cell genotyped as heterozygously- or homozygously-mutated) / (number of total sequenced cells) × 100.

Supplementary Fig. 7

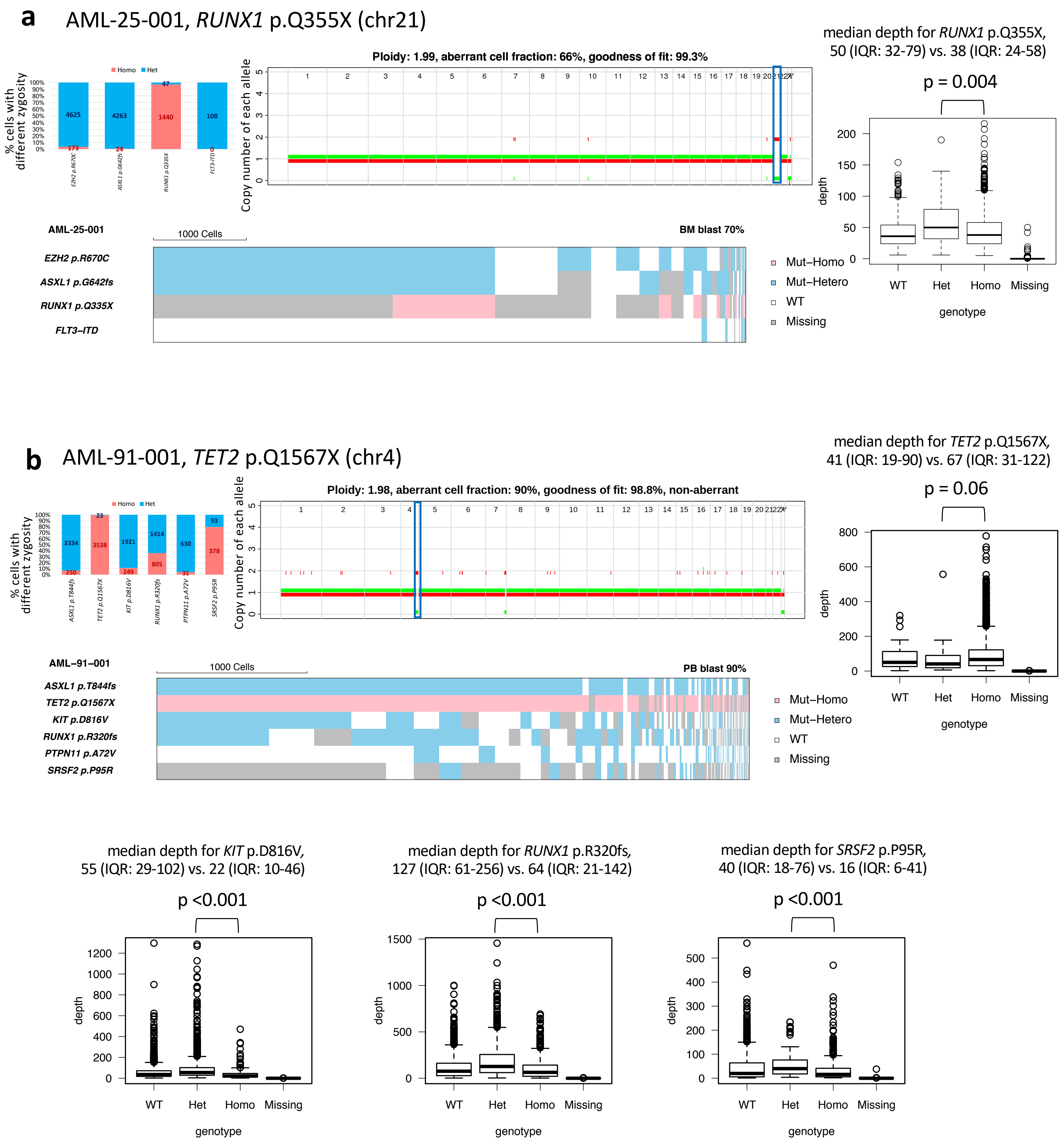

c AML-57-001, *SRSF2* p.P95R (chr17)

median depth for *SRSF2* p.P95R, 10 (IQR: 8-16) vs. 7 (IQR: 6-10)

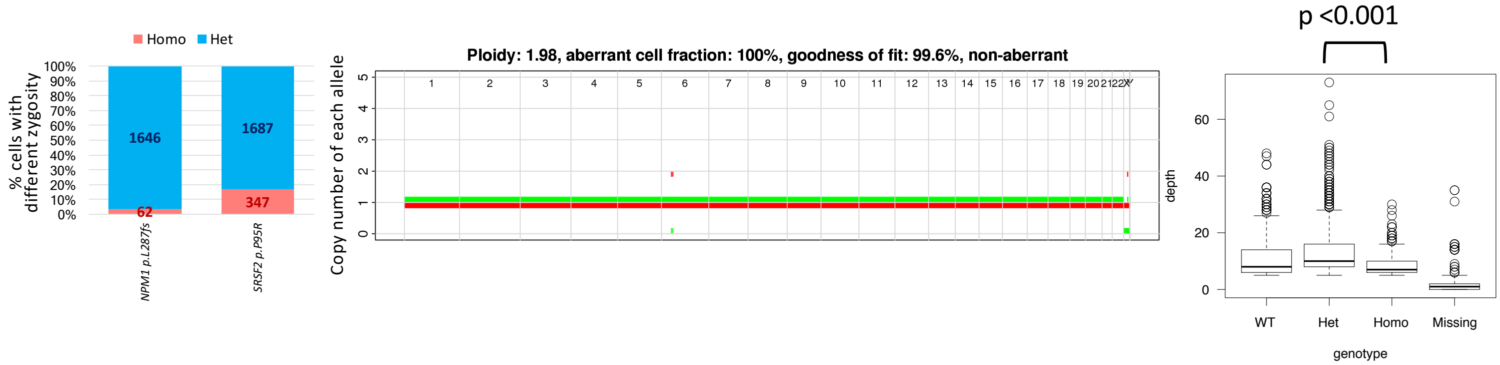

median depth for *NPM1* p.L287fs, 23 (IQR: 16-33) vs. 17 (IQR: 13-23)

d AML-13-001, *NPM1* p.L287fs (chr5)

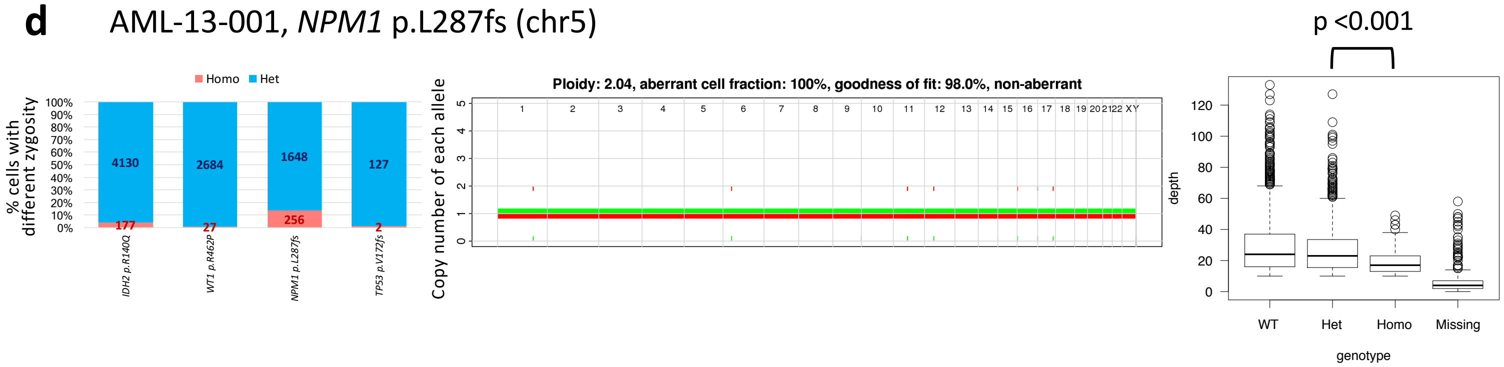

Supplementary Fig. 7. Representative cases with highly homozygous variants analyzed by SNP array. The bar graphs on the left show the distribution of zygosity for each indicated variant. Cells that were genotyped as having heterozygous and homozygous mutations are shown in blue and red, respectively. The numbers on the bars represent the number of cells with each genotype. The figures in the middle show the distribution of the allele counts for the two alleles (green or red). Box plots show the distributions of depth based on the genotype calling for variants in which >10% of mutated cells were genotyped as homozygously mutated. The thick line within each box represents the median, and the top and bottom edges of the box represent the 25th and 75th percentiles, respectively. The upper and lower whiskers represent the 75th percentile plus 1.5 times the interquartile range and the 25th percentile minus 1.5 times the interquartile range, respectively. Heat maps incorporating the zygosity information are also shown for cases with validated homozygosity. CN-LOH involving the homozygously-called variant loci was detected by SNP array in cases with highly homozygous **(a)** *RUNX1* p.Q355X and **(b)** *TET2* p.Q1567X variants. Cases with homozygously called **(b)(c)** *SRSF2* p.P95R and **(d)** *NPM1* p.L287fs variants did not have CN-LOH involving the mutant loci. N= total sequenced cells for each sample: 6366 cells for (a) AML-25-001, 3941 cells for (b) AML-91-001, 8666 cells for (c) AML-57-001, 9345 cells for (d) AML-13-001. Two-sided Mann-Whitney *U* test was used without adjustment for multiple comparisons. The raw p value comparing the sequencing depth of cells with heterozygous vs homozygous calling was (a) 0.00384, (b) 0.0625 (*TET2*), 1.98e-44 (*KIT*), 1.02e-45 (*RUNX1*), 0.00000000228 (*SRSF2*), (c) 1.52e-32, and (d) 9.4e-17. Mut-Homo, homozygously mutated; Mut-Hetero, heterozygously mutated; WT, wild type; Missing, missing genotype; Het, heterozygous; Homo, homozygous; IQR, interquartile range.

Supplementary Fig. 8a

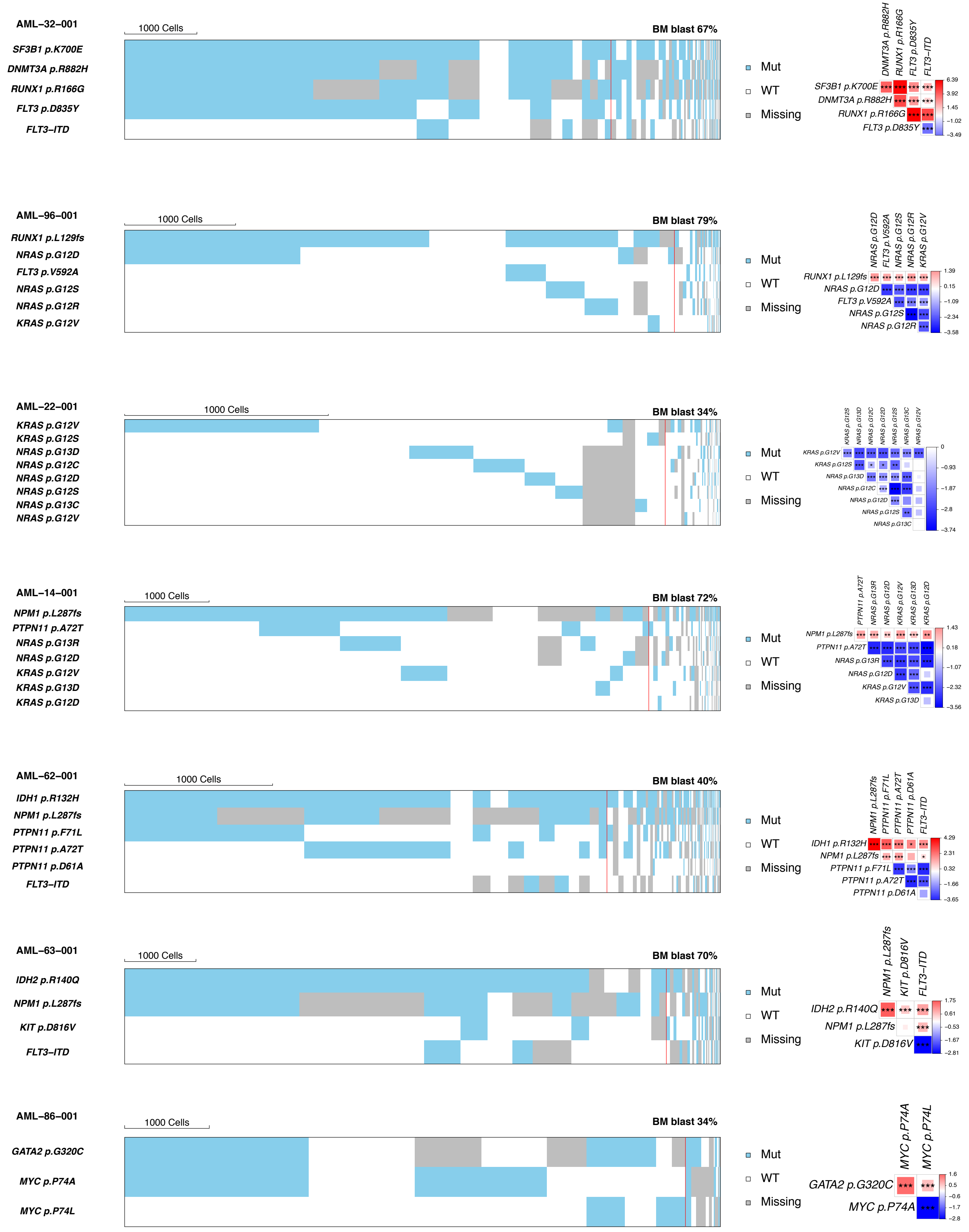

Supplementary Fig. 8b

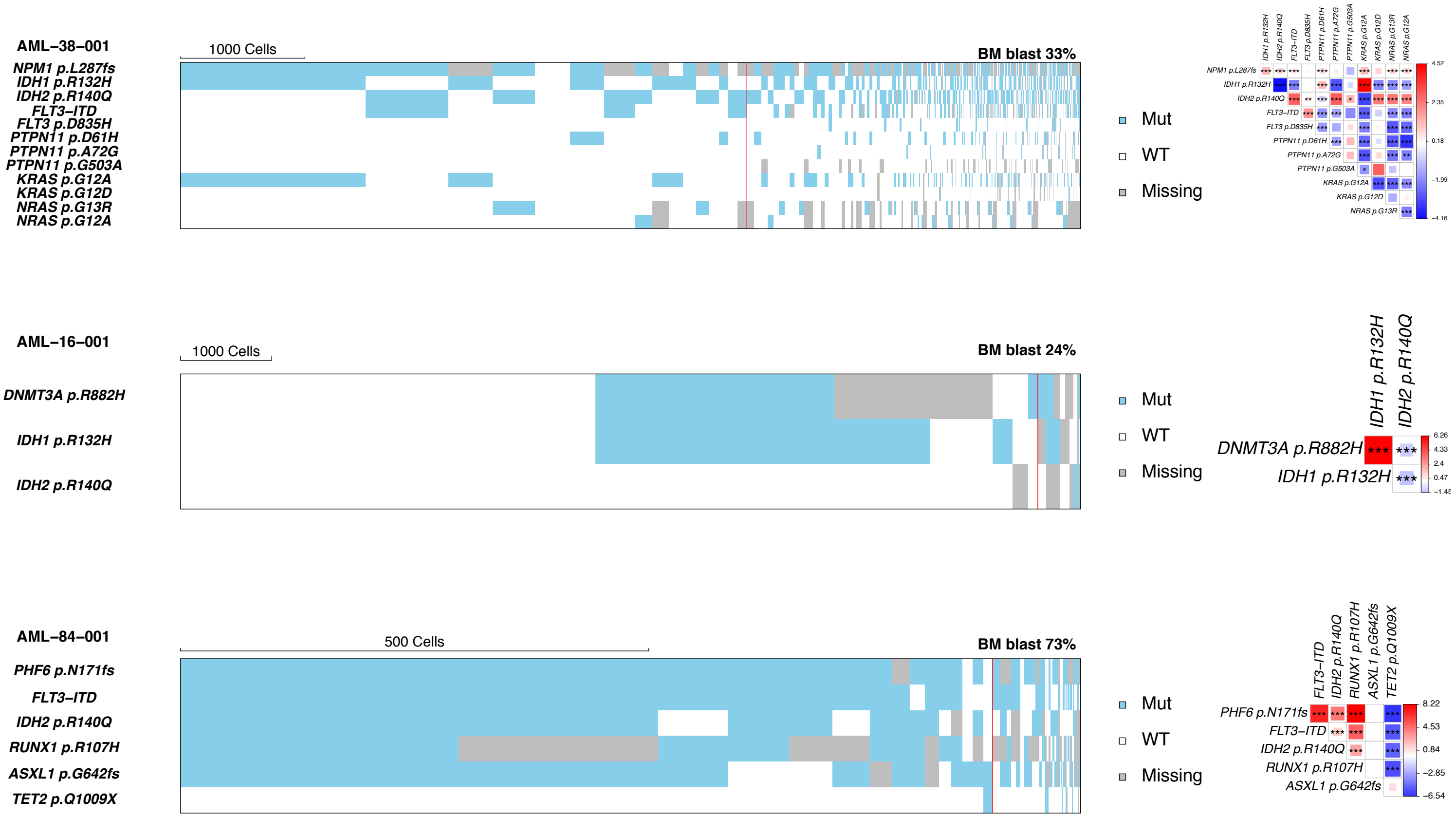

Supplementary Fig. 8c

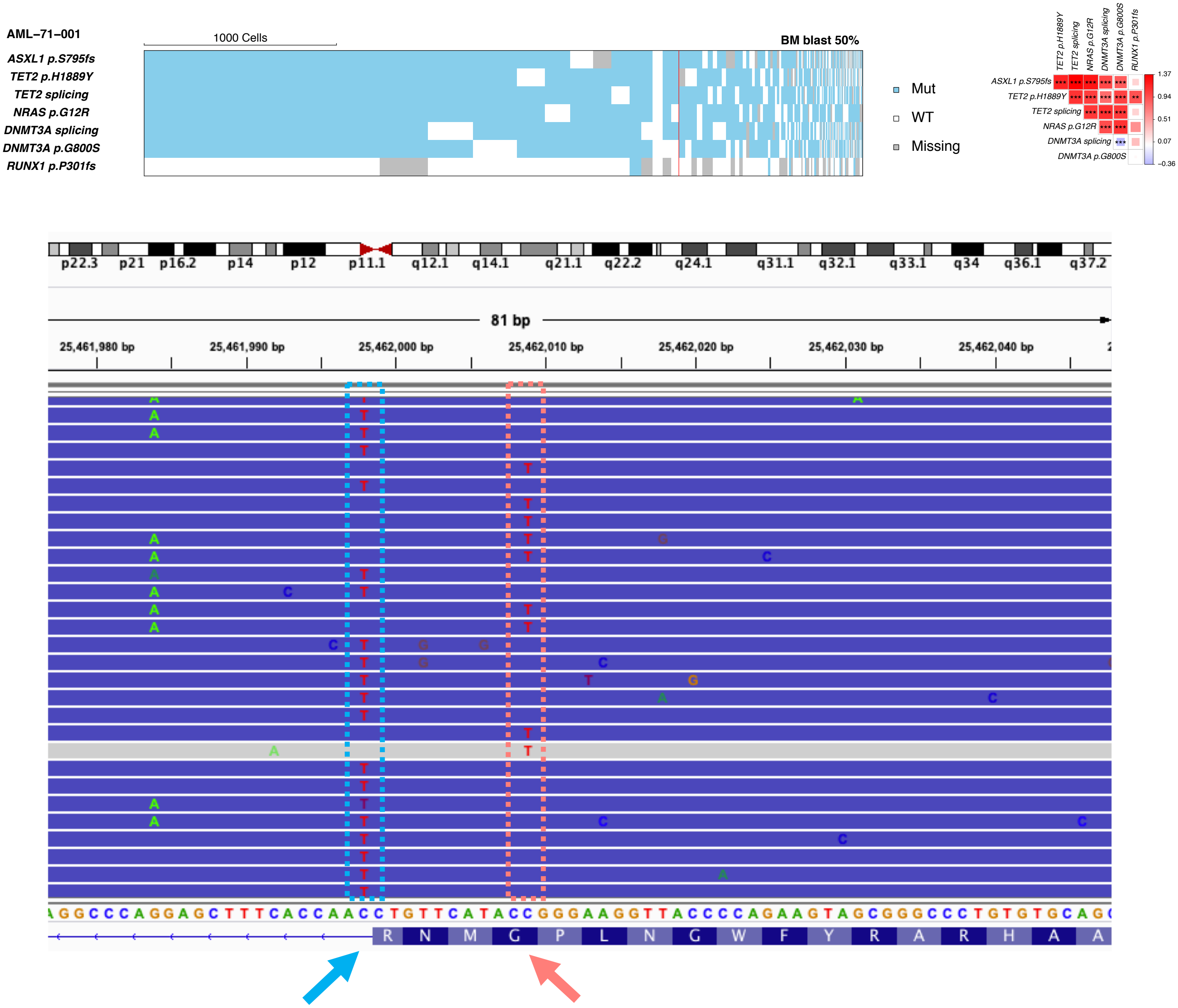

DNMT3A splicing
chr2: 25,461,998, C→T

DNMT3A p.G800S
chr2: 25,462,009, C→T

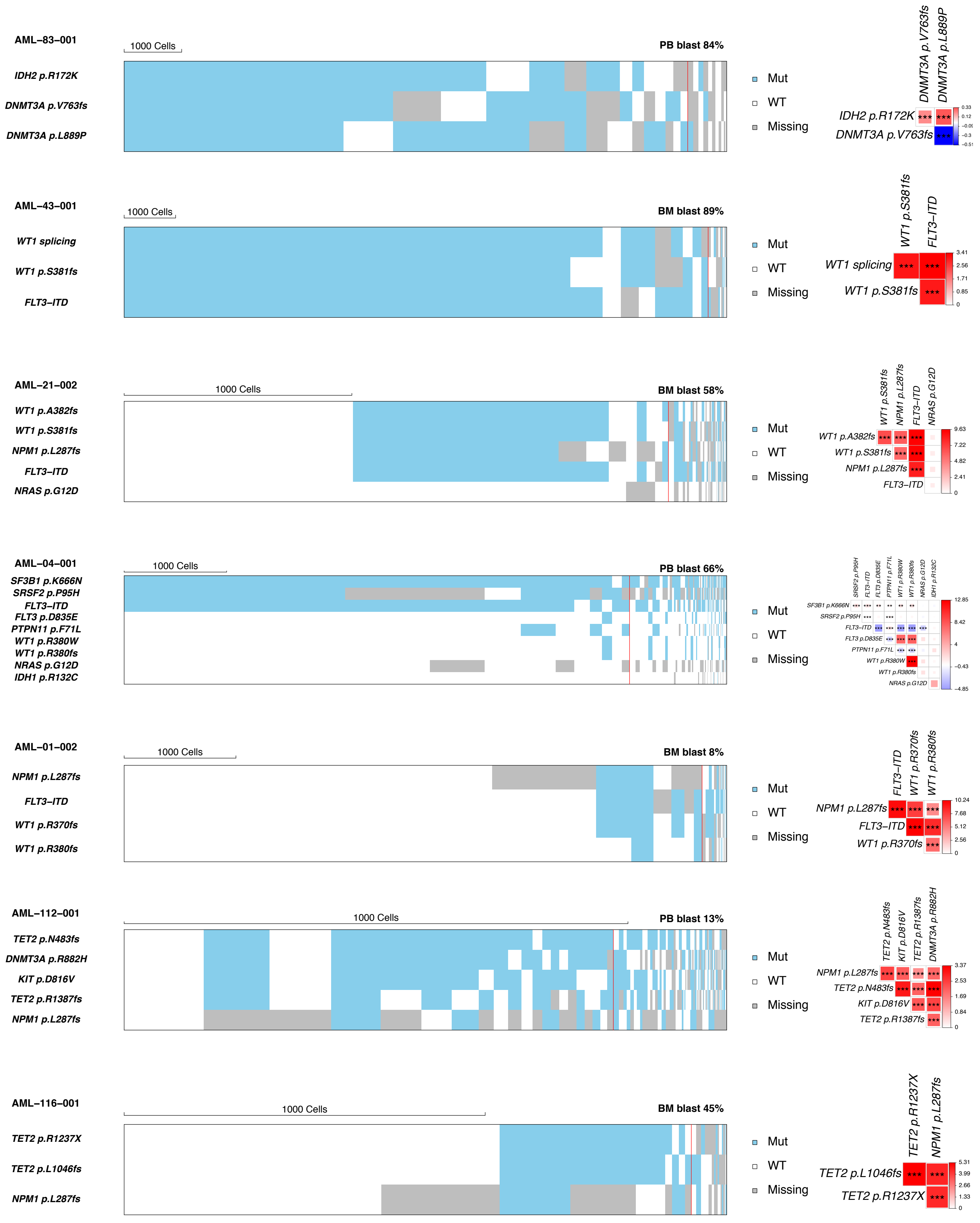
AML–83–001
1000 Cells
PB blast 84%
IDH2 p.R172K
DNMT3A p.V763fs
DNMT3A p.L889P
Mut
WT
Missing
DNMT3A p.V763fs
DNMT3A p.L889P
IDH2 p.R172K
DNMT3A p.V763fs
AML–43–001
1000 Cells
BM blast 89%
WT1 splicing
WT1 p.S381fs
FLT3–ITD
Mut
WT
Missing
WT1 p.S381fs
FLT3–ITD
WT1 splicing
WT1 p.S381fs
3.41
2.56
1.71
0.85
0
AML–21–002
1000 Cells
BM blast 58%
WT1 p.A382fs
WT1 p.S381fs
NPM1 p.L287fs
FLT3–ITD
NRAS p.G12D
Mut
WT
Missing
WT1 p.S381fs
NPM1 p.L287fs
FLT3–ITD
NRAS p.G12D
WT1 p.A382fs
WT1 p.S381fs
NPM1 p.L287fs
FLT3–ITD
9.63
7.22
4.82
2.41
0
AML–04–001
1000 Cells
PB blast 66%
SF3B1 p.K666N
SRSF2 p.P95H
FLT3–ITD
FLT3 p.D835E
PTPN11 p.F71L
WT1 p.R380W
WT1 p.R380fs
NRAS p.G12D
IDH1 p.R132C
Mut
WT
Missing
12.85
8.42
4
−0.43
−4.85
AML–01–002
1000 Cells
BM blast 8%
NPM1 p.L287fs
FLT3–ITD
WT1 p.R370fs
WT1 p.R380fs
Mut
WT
Missing
FLT3–ITD
WT1 p.R370fs
WT1 p.R380fs
NPM1 p.L287fs
FLT3–ITD
WT1 p.R370fs
10.24
7.68
5.12
2.56
0
AML–112–001
1000 Cells
PB blast 13%
TET2 p.N483fs
DNMT3A p.R882H
KIT p.D816V
TET2 p.R1387fs
NPM1 p.L287fs
Mut
WT
Missing
TET2 p.N483fs
KIT p.D816V
TET2 p.R1387fs
DNMT3A p.R882H
NPM1 p.L287fs
TET2 p.N483fs
KIT p.D816V
TET2 p.R1387fs
3.37
2.53
1.69
0.84
0
AML–116–001
1000 Cells
BM blast 45%
TET2 p.R1237X
TET2 p.L1046fs
NPM1 p.L287fs
Mut
WT
Missing
TET2 p.R1237X
NPM1 p.L287fs
TET2 p.L1046fs
TET2 p.R1237X
5.31
3.99
2.66
1.33
0

Supplementary Fig. 8. Cellular-level co-occurrence and exclusivity patterns among AML driver mutations. (a) Representative cases with multiple mutations involving RTK/RAS/MAP kinase signaling pathway genes (*FLT3*, *NRAS*, *KRAS*, *PTPN11*, *KIT,* and *MYC*) showing the cellular-level mutual exclusivity. **(b)** Representative cases with multiple functionally-redundant mutations (*IDH1*, *IDH2*, and *TET2*) showing the cellular-level mutual exclusivity. **(c)** Representative cases with multiple mutations in the same gene (*DNMT3A*, *WT1*, and *TET2*) showing the cellular-level co-occurrence. In AML-71-001, allelic-level exclusivity of the two *DNMT3A* mutations was visualized using Integrative Genomics Viewer (IGV). The region shown in IGV track is covered by one amplicon (*DNMT3A_c5*), and each sequencing read (dark blue) represents each allele. The sequencing reads with *DNMT3A* splicing mutation did not harbor *DNMT3A* p.G800S mutation, and vice versa, indicating that these mutations did not co-occur on the same alleles, and the two mutations presented as biallelic mutations. The statistical significance of cell-level co-occurrence and mutual exclusivity was analyzed based on the false discovery rate (FDR) is indicated by the asterisks (*FDR < 0.1, **FDR < 0.05, ***FDR < 0.001); two-sided Fisher's exact with adjustment for multiple testing using Benjamini-Hochberg method.

Supplementary Fig. 9

a

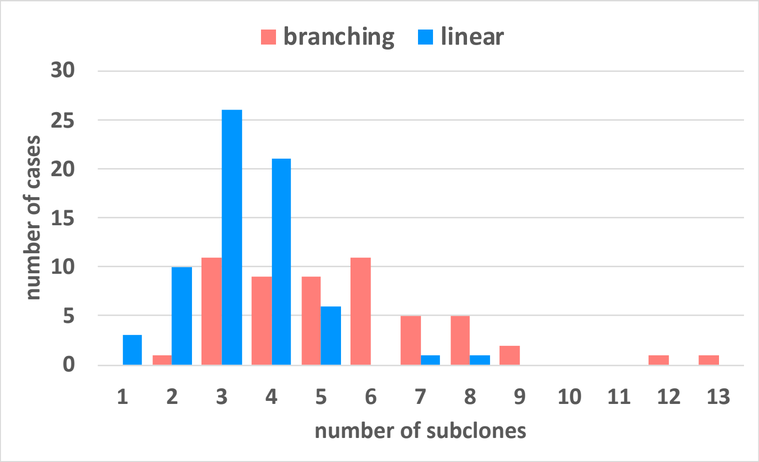

b

	branching	linear	
	N=55 (% or IQR)	N=68 (% or IQR)	*p*
**median WBC** (× $10^3/\mu L$)	7.3 (2.7-28.2)	8.3 (3.6-29.8)	0.53
**median HGB** (g/dL)	9.3 (8.4-9.9)	9.0 (8.5-10.1)	0.99
**median PLT** (× $10^3/\mu L$)	59 (32-89)	49 (26-83)	0.38
**median BM blasts** (%)	49 (31-67)	44 (29-68)	0.99
**median PB blasts** (%)	27 (5-57)	26 (4.8-56.3)	0.97
**median LDH** (U/L)	784 (540-1285)	656 (396-1132)	0.20
**median age** (y)	59 (50-73)	66 (53-72)	0.77
**median number of subclones**	5 (4-7)	3 (3-4)	<0.001
**median Shannon index**	1.83 (1.45-2.22)	1.35 (1.00-1.57)	<0.001
**sex**			
female	24 (44)	24 (35)	0.36
male	31 (57)	44 (65)	
**ontogeny**			
de novo	40 (73)	53 (78)	0.53
secondary/therapy-related	15 (27)	15 (22)	
**prior treatment**			
untreated	42 (76)	46 (68)	0.32
treated	13 (24)	22 (32)	

	Shannon index low	Shannon index high	
	N=62 (% or IQR)	N=61 (% or IQR)	*p*
**median WBC** (× $10^3/\mu L$)	7.0 (3.5-28.6)	12 (3.4-30.4)	0.55
**median HGB** (g/dL)	9.3 (8.4-10.3)	9.0 (8.5-9.8)	0.43
**median PLT** (× $10^3/\mu L$)	54 (29-98)	55 (32-80)	0.94
**median BM blasts** (%)	44 (29-66)	51 (30-70)	0.72
**median PB blasts** (%)	25 (4-51)	29 (5-65)	0.33
**median LDH** (U/L)	670 (429-1452)	698 (522-1061)	0.94
**median age** (y)	60 (49-72)	63 (55-73)	0.21
**median number of subclones**	3 (3-3)	5 (4-6)	<0.001
**sex**			
female	24 (39)	24 (39)	1.00
male	38 (61)	37 (61)	
**ontogeny**			0.83
de novo	46 (74)	47 (77)	
secondary/therapy-related	16 (26)	14 (23)	
**prior treatment**			
untreated	44 (71)	44 (72)	1.00
treated	18 (29)	17 (28)	

c

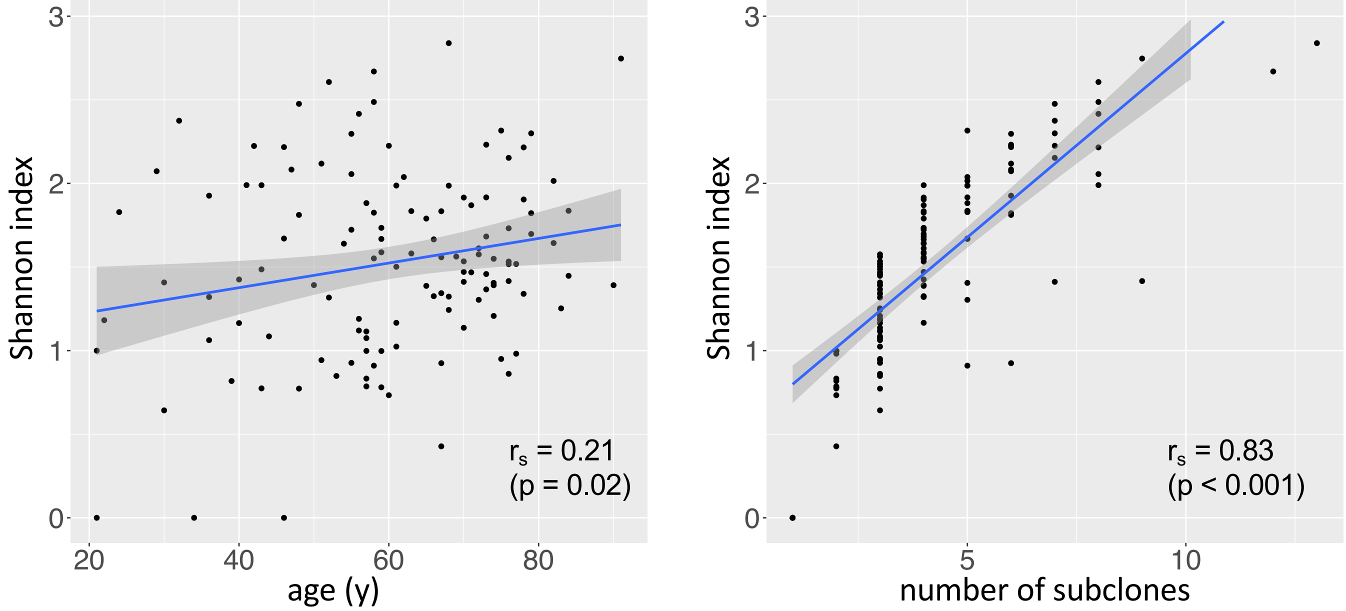

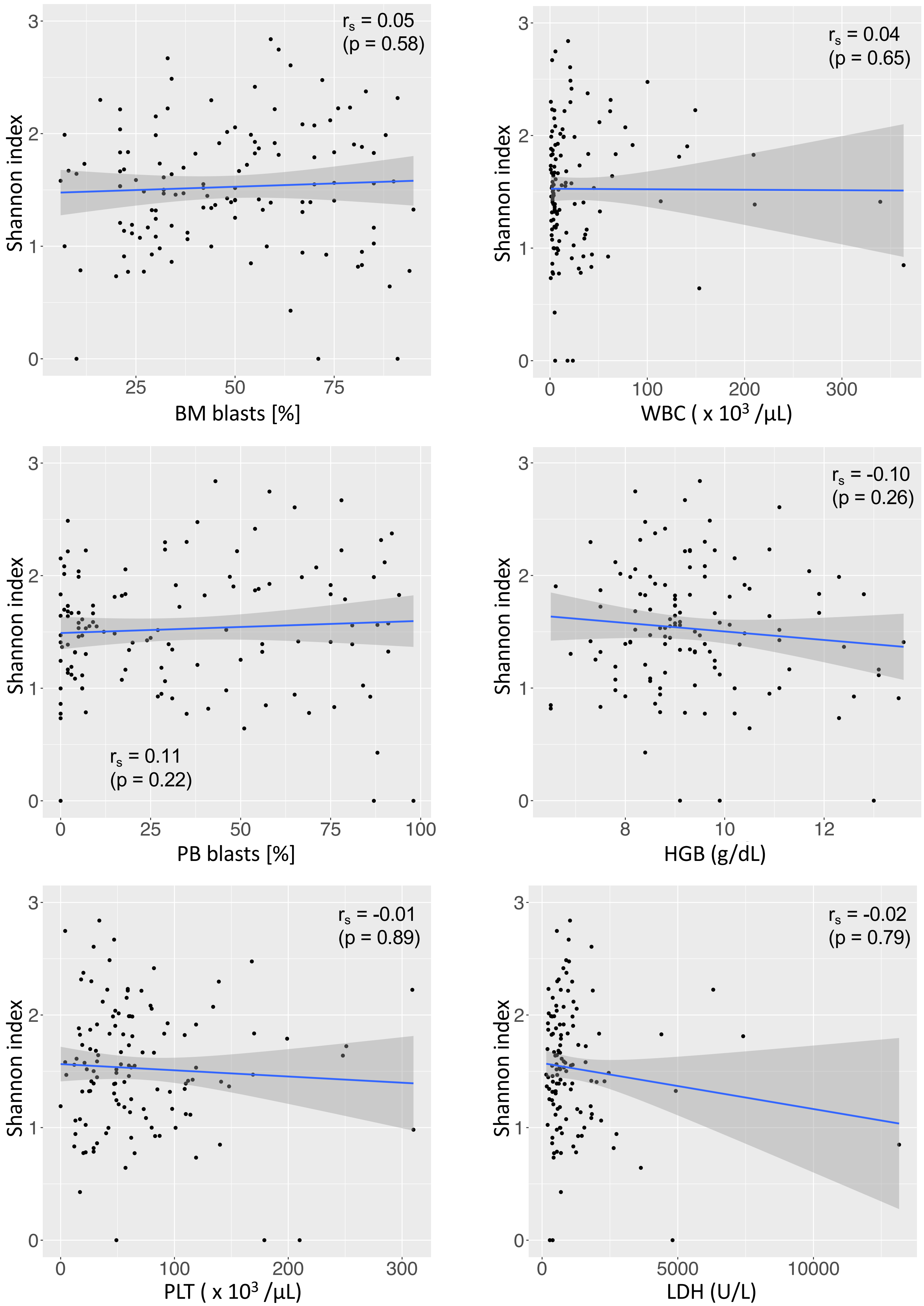

rs = 0.05
(p = 0.58)
Shannon index
BM blasts [%]
rs = 0.04
(p = 0.65)
WBC (x 10³ /µL)
rs = 0.11
(p = 0.22)
PB blasts [%]
rs = -0.10
(p = 0.26)
HGB (g/dL)
rs = -0.01
(p = 0.89)
PLT (x 10³ /µL)
rs = -0.02
(p = 0.79)
LDH (U/L)

Supplementary Fig. 9. Clinical characteristics based on mutation evolution pattern and clonal diversity. (a) A histogram showing the distribution of the number of subclones based on the single-cell DNA sequencing data according to the evolution patterns. **(b)** Clinical and demographic characteristics based on the evolution patterns. Two-sided Mann-Whitney *U* test or Student's *t*-test was used for continuous variables without adjustment for multiple comparisons. Two-sided Fisher's exact test was used for categorizal variables. IQR, interquartile range; WBC, white blood cells; HGB, hemoglobin; PLT, platelets; BM, bone marrow; PB, peripheral blood; LDH, lactate dehydrogenase. **(c)** Correlation of clinical parameters and clonal diversity. In each figure, the X-axis shows the clinical parameter and the Y-axis shows Shannon index. Each dot represents each patient. The linear regression line was added to best fit the distribution of the dots. The shaded area around the trendline represents the 95% confidence intervals. Correlation coefficient (r_s) is shown. N = 123 unique patients.

Supplementary Fig. 10

a

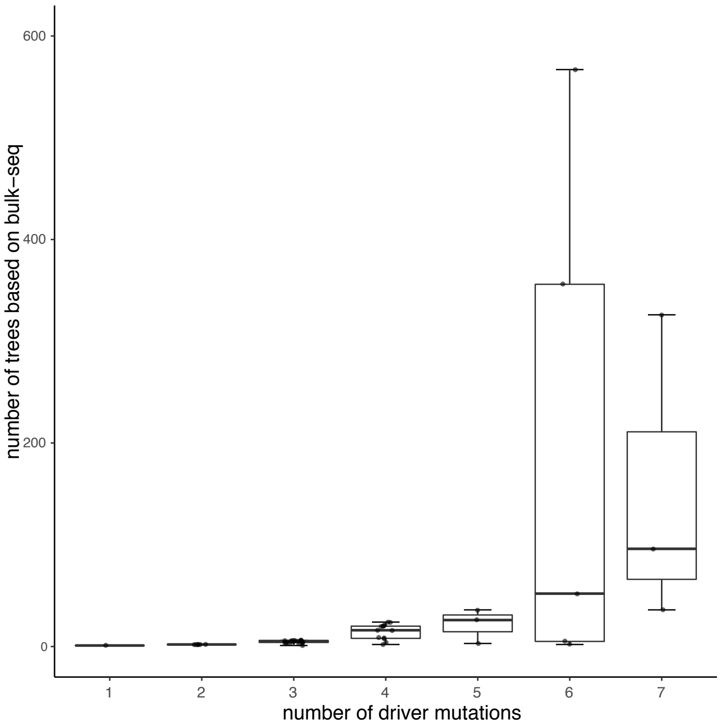

b **AML-86-001**

mutation	bulk VAF
GATA2 p.G320C	0.37
MYC p.P74A	0.28
MYC p.P74L	0.06

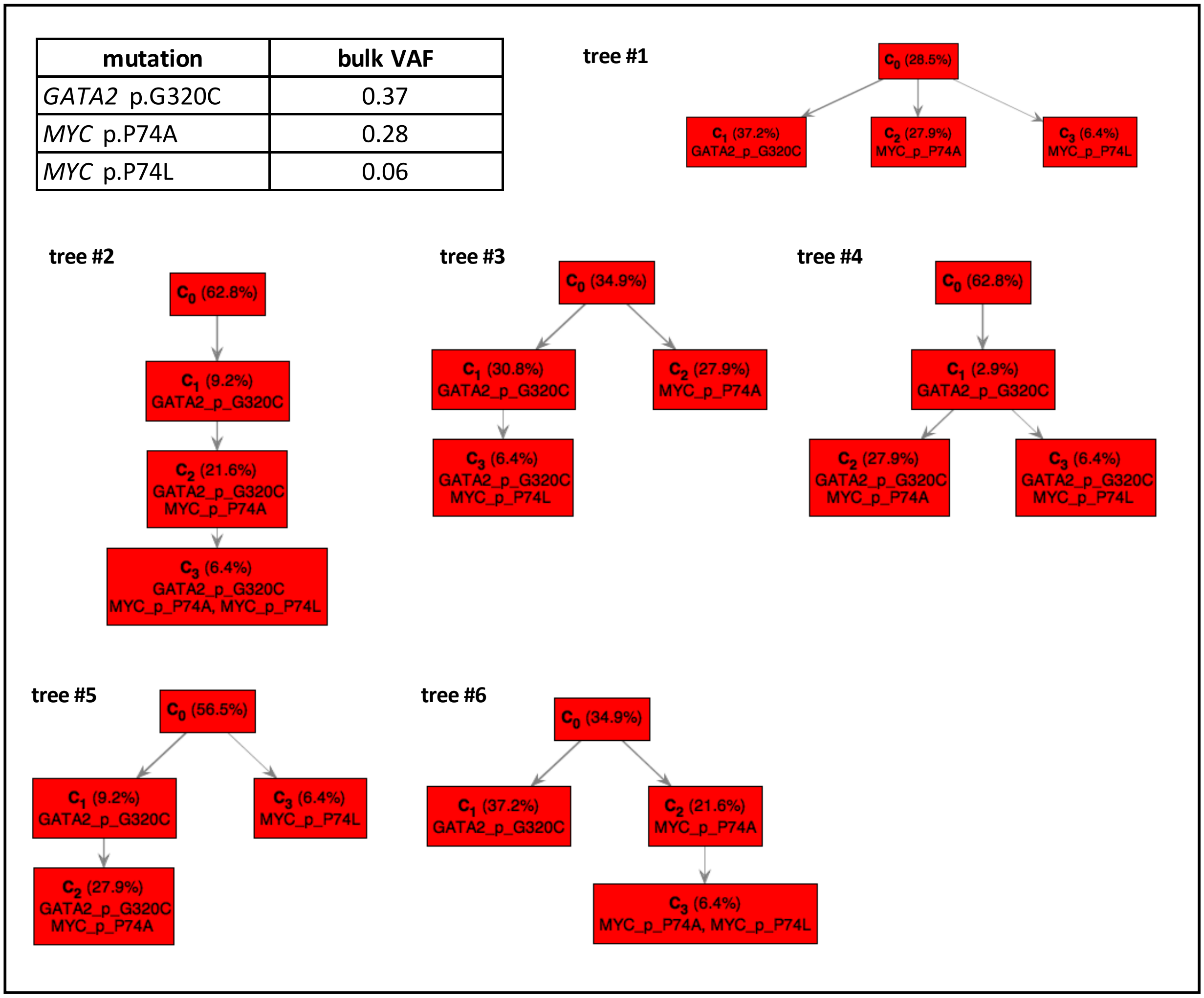

c

AML-18-001

mutation	bulk VAF
IDH2 p.R172K	0.32
RUNX1 splicing	0.28
ASXL1 p.P808fs	0.20

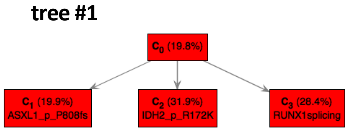

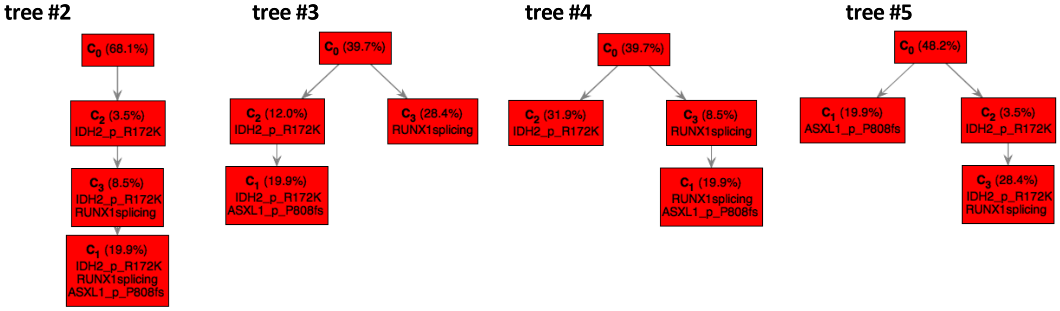

d

AML-12-001

mutation	bulk VAF
NPM1 p.L287fs	0.20
FLT3 p.836_837del	0.09
PTPN11 p.E76V	0.04

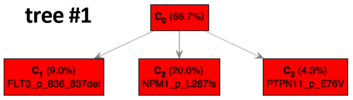

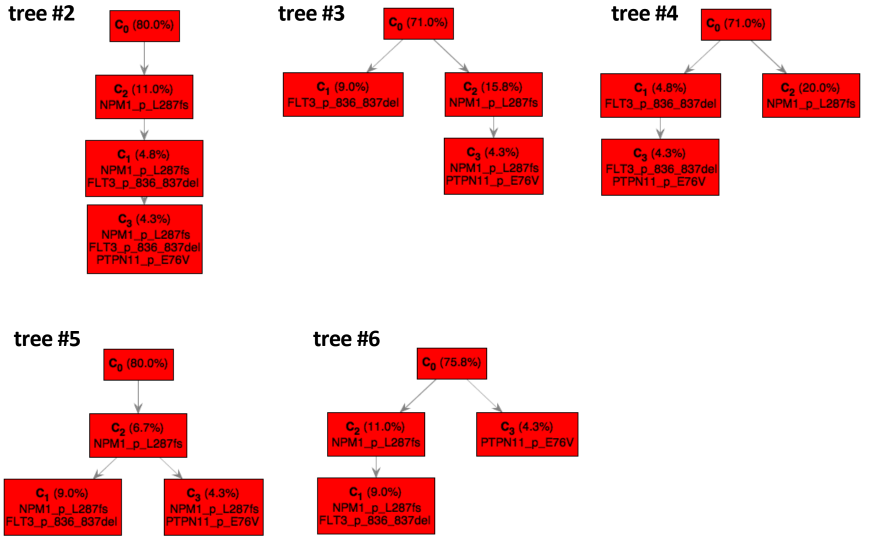

e

AML-29-001

mutation	bulk VAF
SRSF2 p.P95H	0.36
RUNX1 p.R201Q	0.27
ASXL1 p.G642fs	0.13
IDH2 p.R140Q	0.02

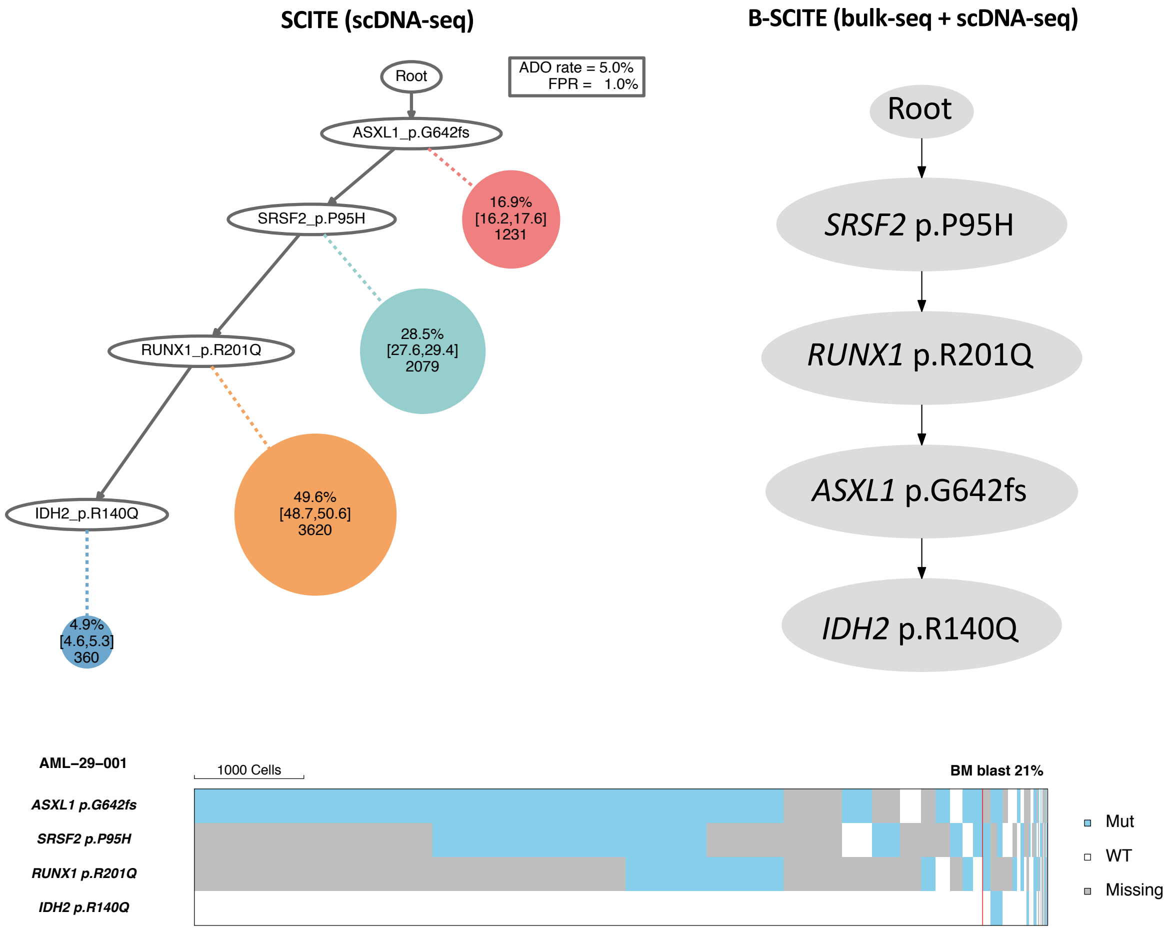

Supplementary Fig. 10. Clonal evolution based on single-cell, bulk, and bulk and single-cell sequencing data. (a) Distribution of the number of possible phylogeny models based on bulk sequencing (bulk-seq) data. The X axis shows the number of driver mutations for each case. The Y axis shows the number of trees generated by TrAp algorithm using bulk-seq data. N=50 patients with available bulk-seq read count data whose mutations were all detected by both scDNA-seq and bulk-seq. The thick line within each box represents the median, and the top and bottom edges of the box represent the 25th and 75th percentiles, respectively. The upper and lower whiskers represent the 75th percentile plus 1.5 times the interquartile range and the 25th percentile minus 1.5 times the interquartile range, respectively. **b-d**, Inference of mutation phylogeny based on bulk-seq data in representative cases. The tables show the variant allele fraction (VAF) based on bulk-seq data (bulk VAF) for each mutation. Each box in the trees represents a subclone based on the combination of genetic aberrations. The number within the box represents the size of subclone. **(b)** A total 6 tree models were generated based on bulk-seq data using TrAP algorithm, whereas scDNA-seq data identified tree #4 as the best fit model. **(c)** A total 5 tree models were generated based on bulk-seq data using TrAP algorithm, whereas scDNA-seq data identified tree #2 as the best fit model. **(d)** A total 6 tree models were generated based on bulk-seq data using TrAP algorithm, whereas scDNA-seq data identified tree #5 as the best fit model. **(e)** Inference of mutation phylogeny based on the combined bulk-seq and scDNA-seq data in AML-29-001. The table shows the bulk VAF data for each mutation. A tree based on scDNA-seq data generated by SCITE (left) showed *ASXL1* mutation as an initial event, whereas a tree based on combined bulk-seq and scDNA-seq data generated by B-SCITE (right) showed *SRSF2* mutation as an initial event. The bottom heatmap shows a large proportion of cells being ungenotyped for *SRSF2* and *RUNX1* mutations.

Supplementary Fig. 11

a

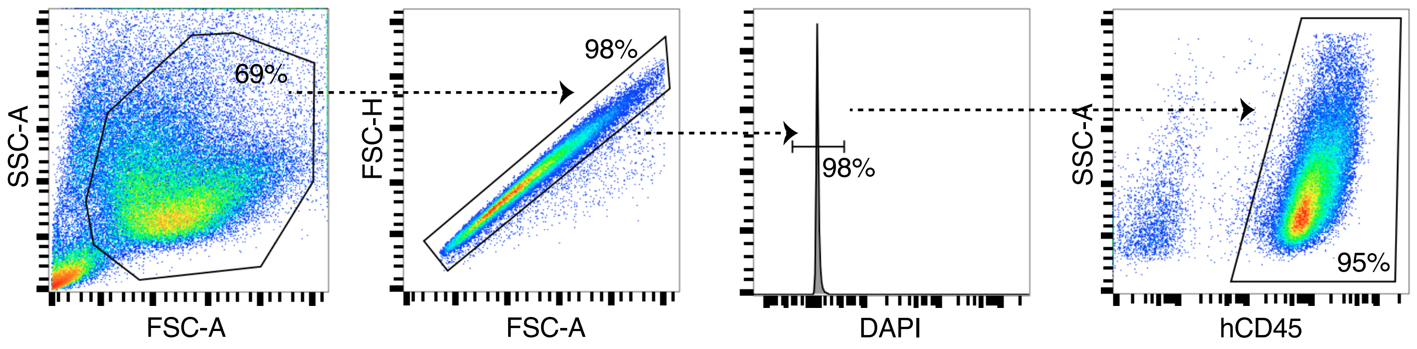

b

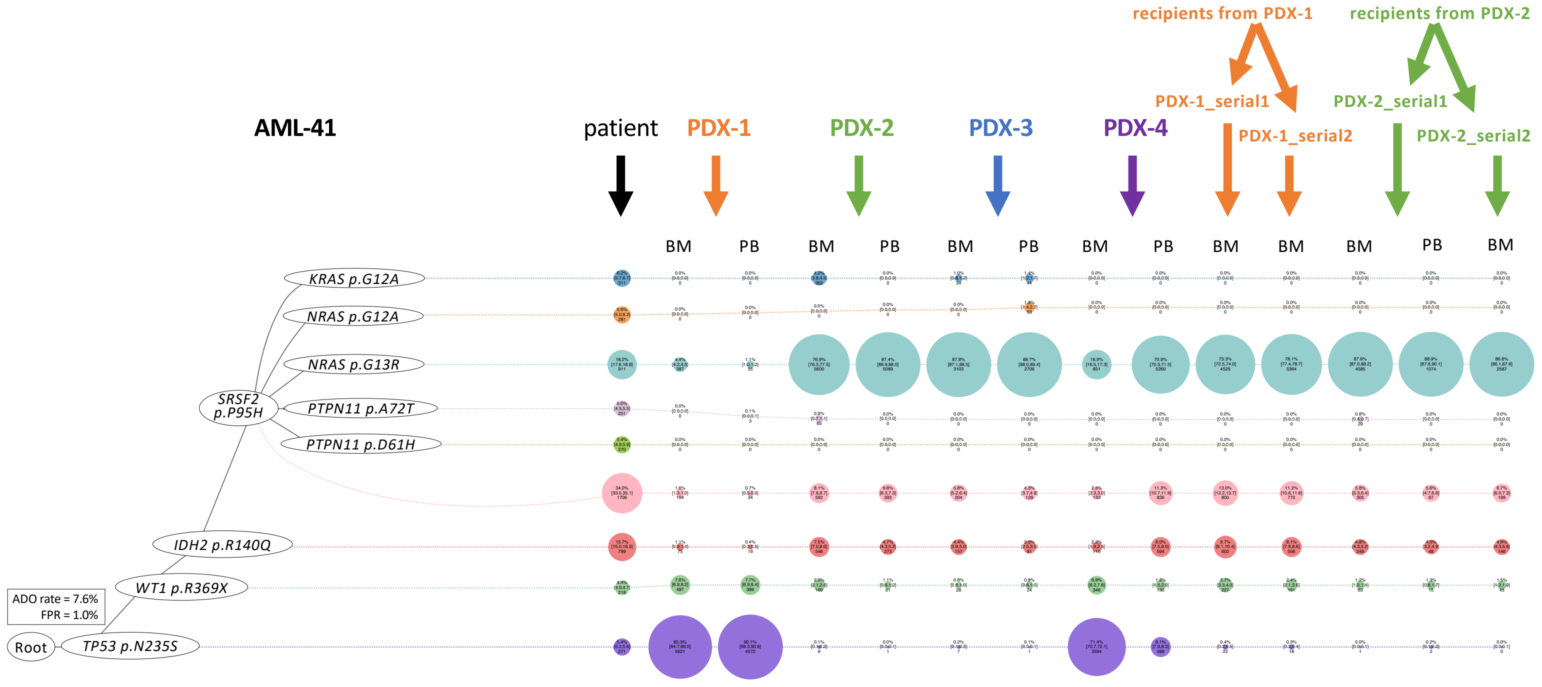

Supplementary Fig. 11. Clonal diversity in patient-derived xenograft (PDX) model. (a) Gating strategy for PDX samples. Cells were gated based on FSC-A and SSC-A to remove the debris and laser noise. Cells were then gated on singlets based on FSC-A and FSC-H. Viable patients' derived cells ($DAPI^{-}hCD45^{+}$) were then sorted based on SSC-A and hCD45. FSC, forward scatter; SSC, side scatter. **(b)** Clonal structures in AML-41 based on single-cell DNA sequencing from human and PDX samples. Parallel subclones carrying *TP53* p.N235S, *WT1* p.R369X, *IDH2* p.R140Q, and *SRSF2* p.P95H, either with *KRAS* p.G12A, *NRAS* p.G12A, *NRAS* p.G13R, *PTPN11* p.A72T, or *PTPN11* p.D61H. Expansion of *NRAS* p.G13R clone and regression of other clones were observed in both primary and secondary recipients. Overall, the clonal architecture was similar between bone marrow (BM) and peripheral blood (PB) samples from each mouse.

Supplementary Fig. 12

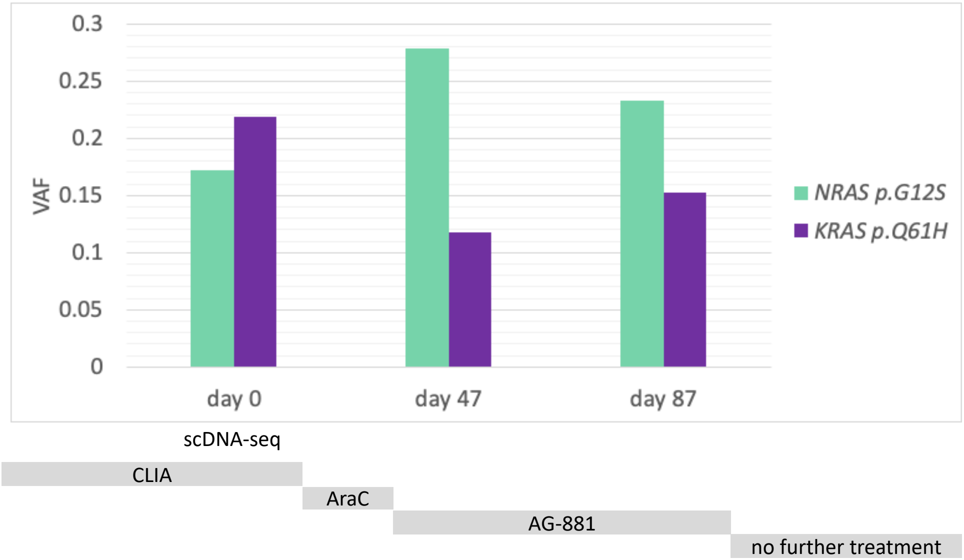

Supplementary Fig. 12. Trend of variant allele fraction in AML-67 during the therapy. Bar graph shows the variant allele fraction (VAF) from bulk sequencing in AML-67 for *NRAS* p.G12S and *KRAS* p.Q61H mutations for three timepoints. The X axis represents the timing of bulk sequencing, and the Y axis represents VAF. Day 0 corresponds to the date of sample collection for single-cell DNA sequencing (scDNA-seq). VAF of *NRAS* p.G12S mutation increased, whereas VAF of *KRAS* p.Q61H mutation decreased during the treatment. CLIA, cladribine, idarubicin and cytarabine; AraC, cytarabine.

Supplementary Fig. 13

AML-103

a

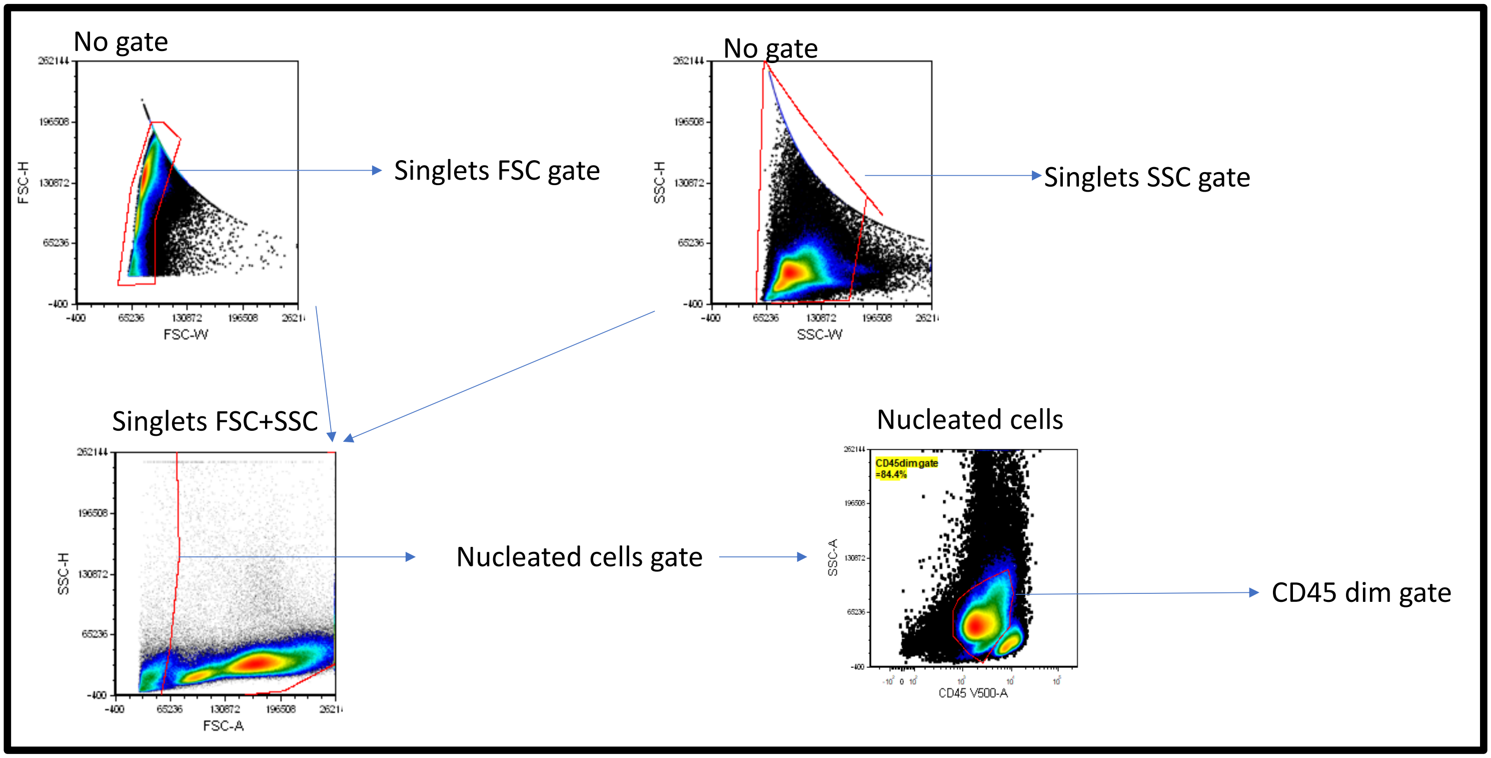

b

CD45 dim gate

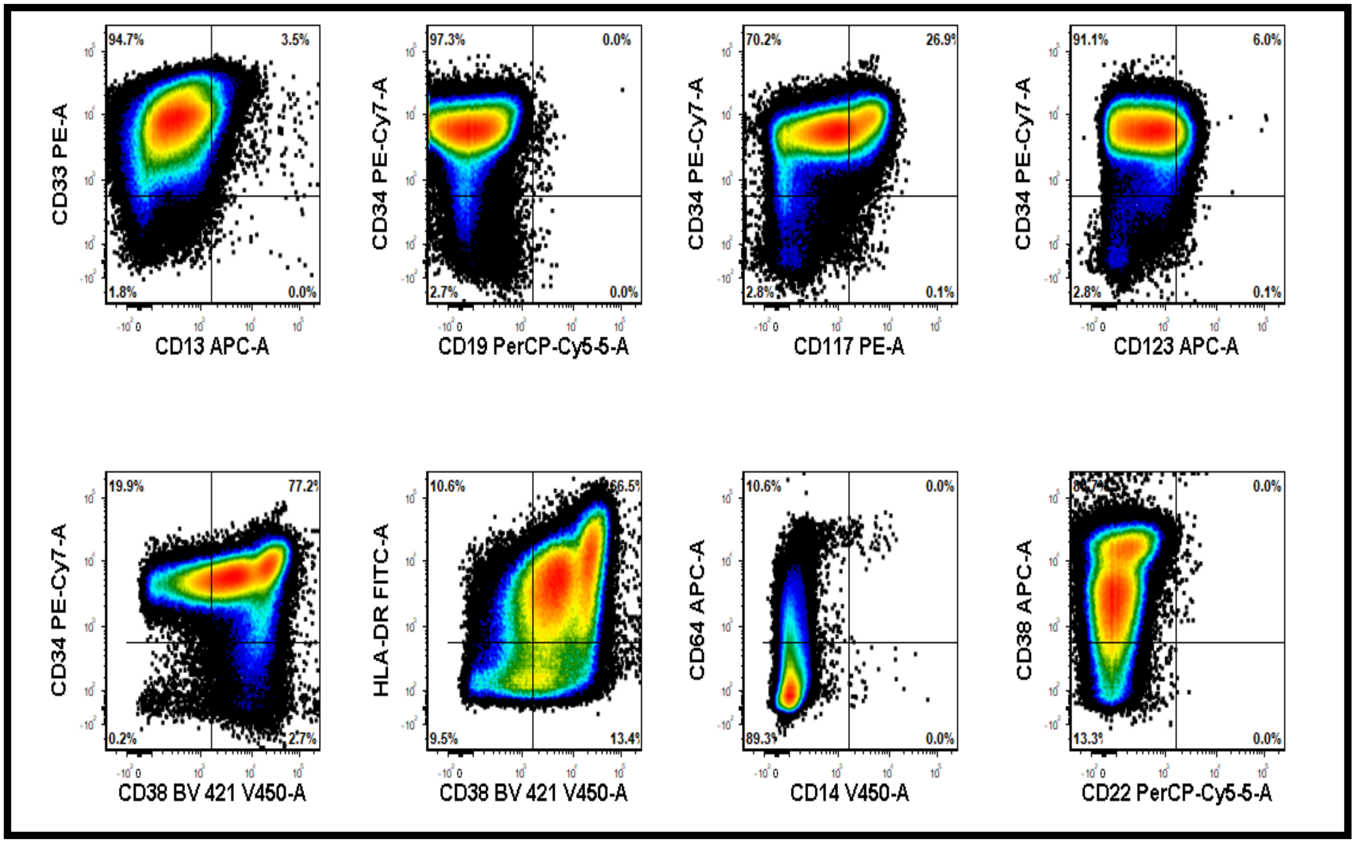

AML-101

c

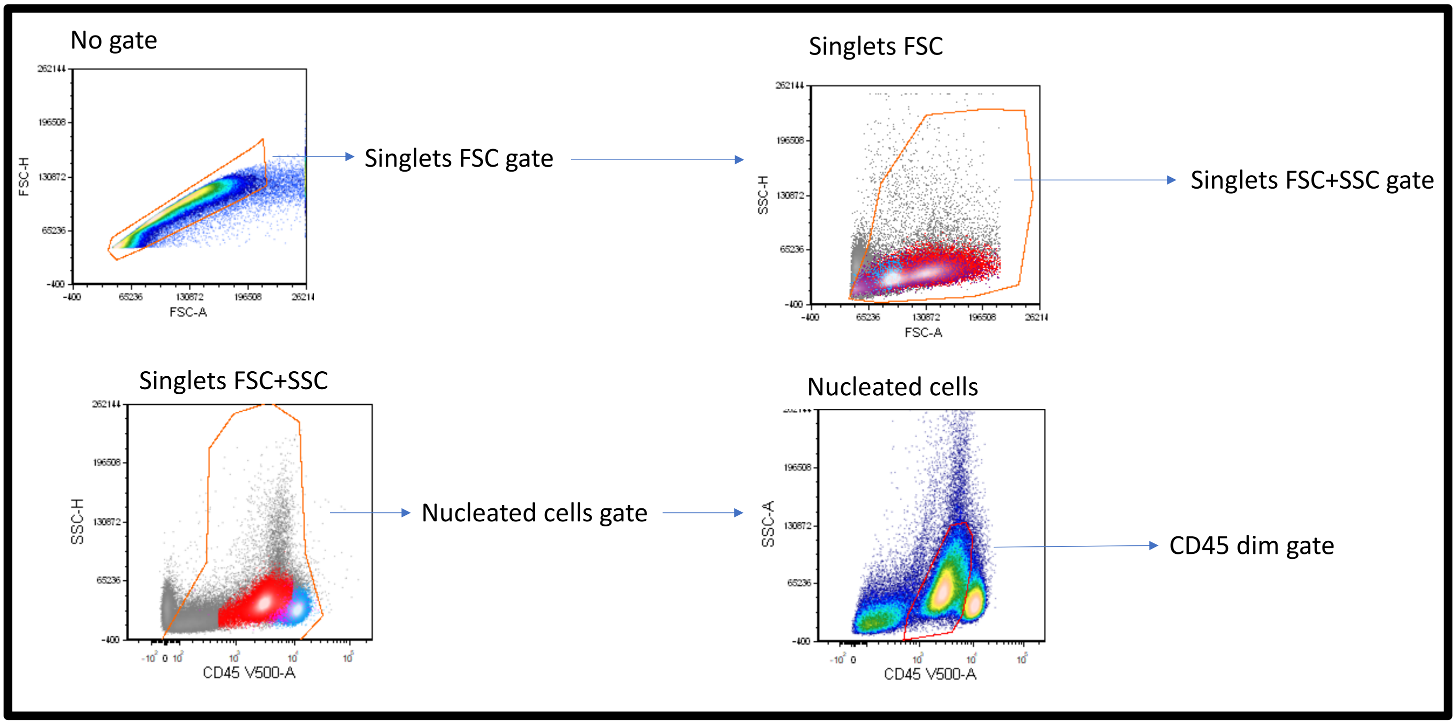

d

CD45 dim gate

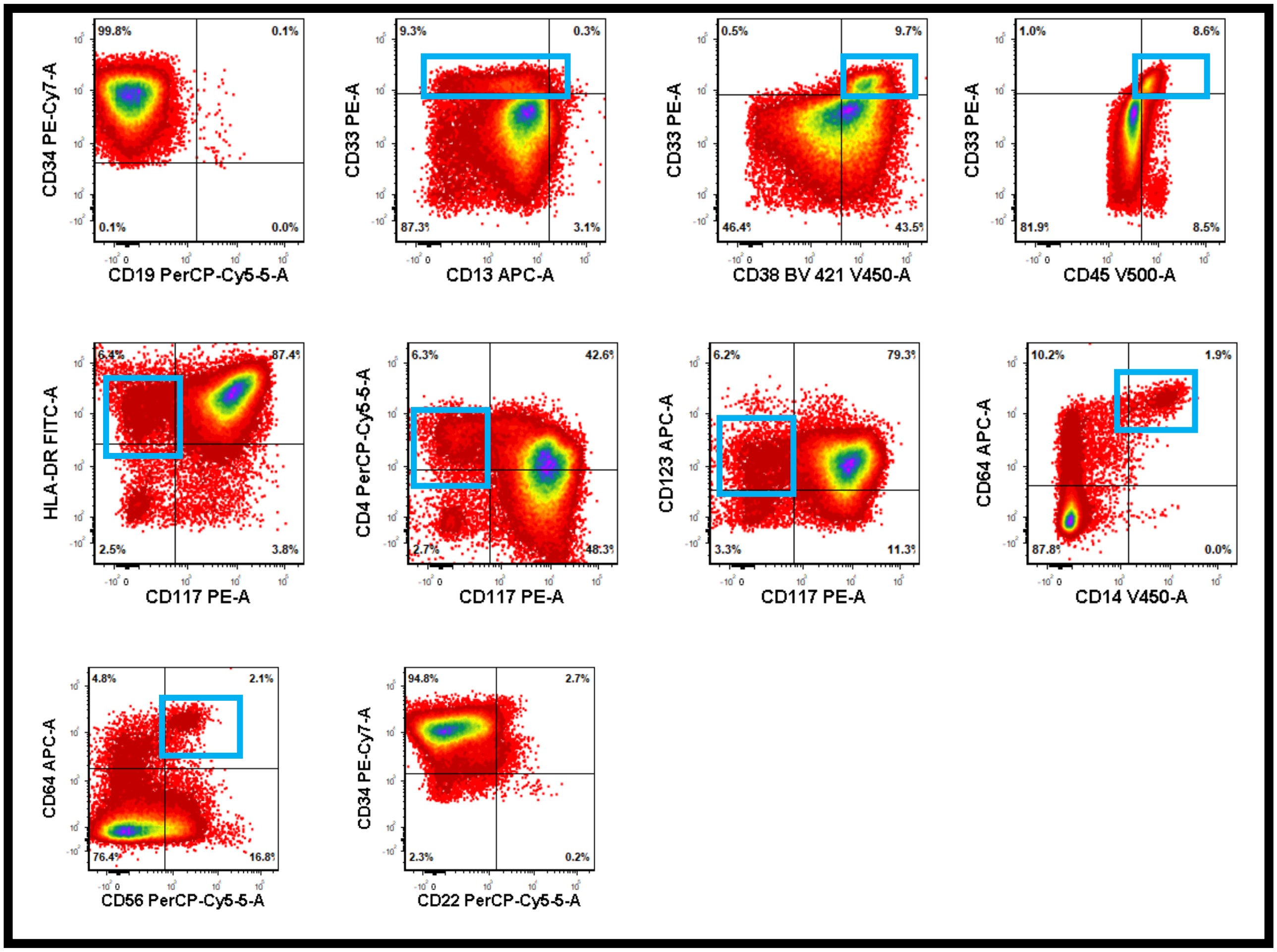

Supplementary Fig. 13. Clinical immunophenotype assessed by multicolor flow cytometry. **(a)** Gating strategy for clinical flow cytometry in AML-103. The right bottom figure corresponds to the CD45^{dim}-gated population in Fig. 5d. **(b)** Immunophenotype for the CD45^{dim}-gated leukemic blasts in AML-103. **(c)** Gating strategy for clinical flow cytometry in AML-101. **(d)** Immunophenotype for the CD45^{dim}-gated leukemic blasts in AML-101. A dominant population was CD34^{+} myeloblasts. In addition, a small population of monocytes with aberrant expression (CD64^{+}, CD56^{+}, CD33^{+}, CD117^{-}, decreased CD13, indicated with blue rectangles) was observed. FSC, forward scatter; SSC, side scatter.

Supplementary Fig. 14

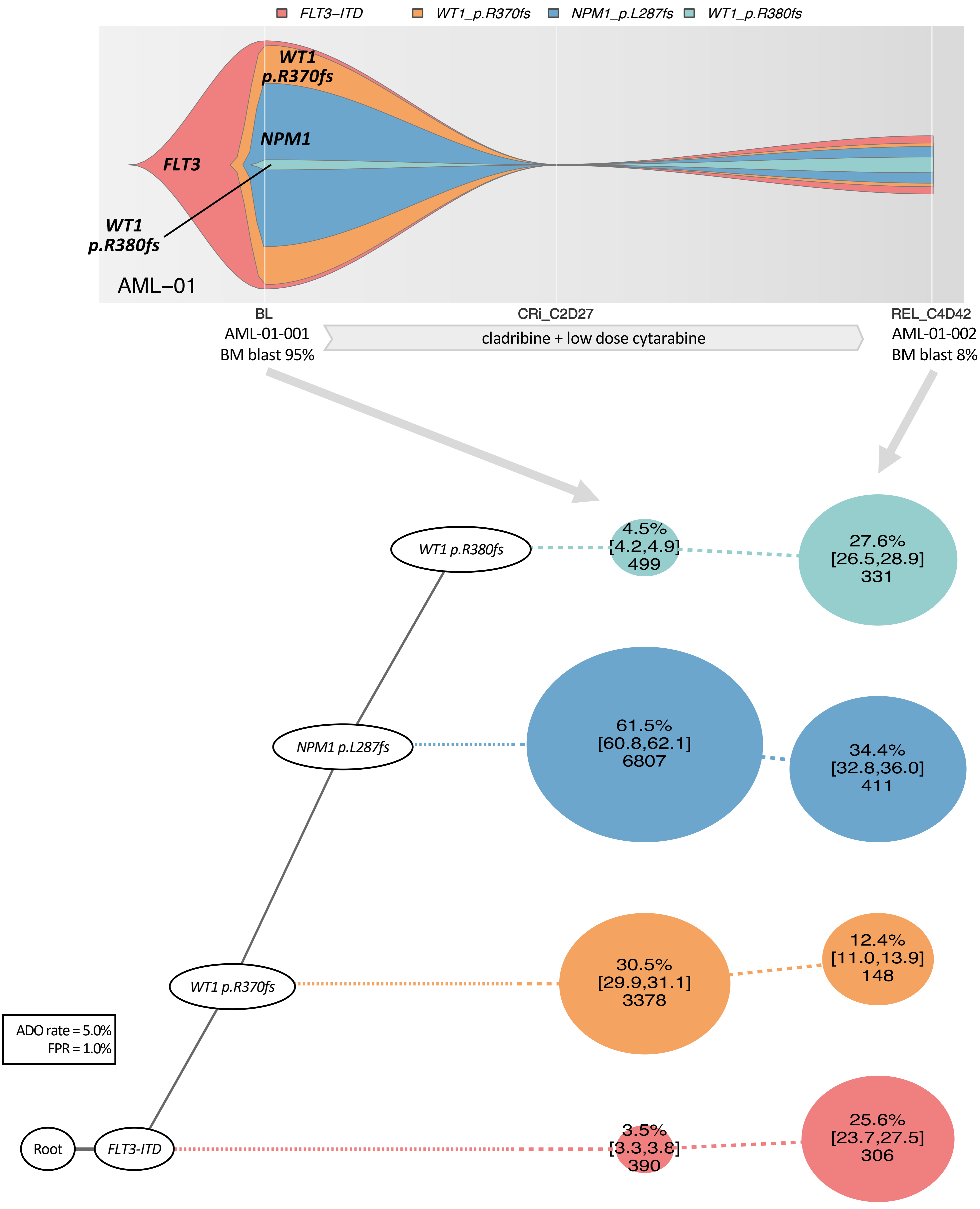

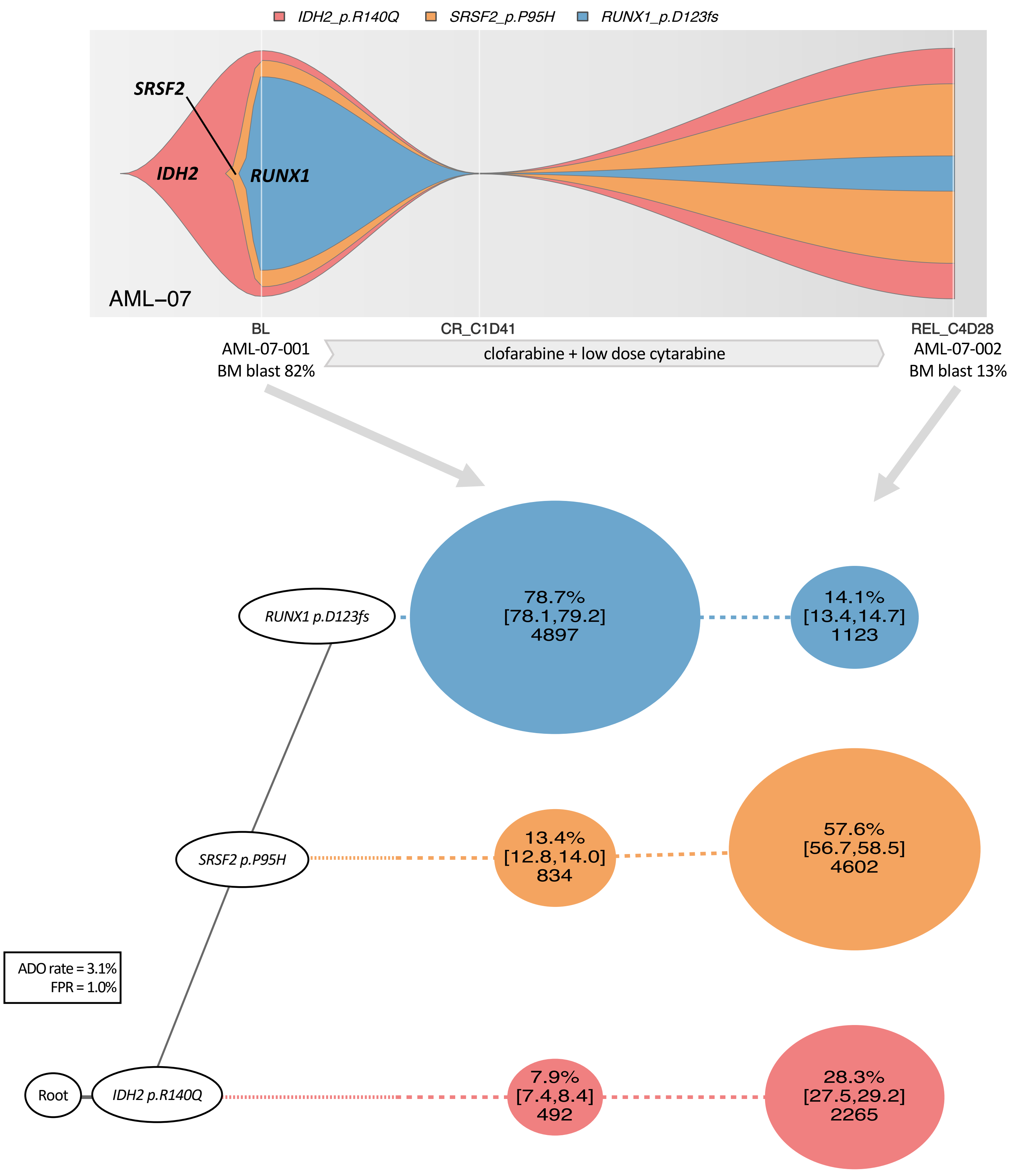
IDH2_p.R140Q
SRSF2_p.P95H
RUNX1_p.D123fs
SRSF2
IDH2
RUNX1
AML–07
BL
AML-07-001
BM blast 82%
CR_C1D41
clofarabine + low dose cytarabine
REL_C4D28
AML-07-002
BM blast 13%
RUNX1 p.D123fs
78.7%
[78.1,79.2]
4897
14.1%
[13.4,14.7]
1123
SRSF2 p.P95H
13.4%
[12.8,14.0]
834
57.6%
[56.7,58.5]
4602
ADO rate = 3.1%
FPR = 1.0%
Root
IDH2 p.R140Q
7.9%
[7.4,8.4]
492
28.3%
[27.5,29.2]
2265

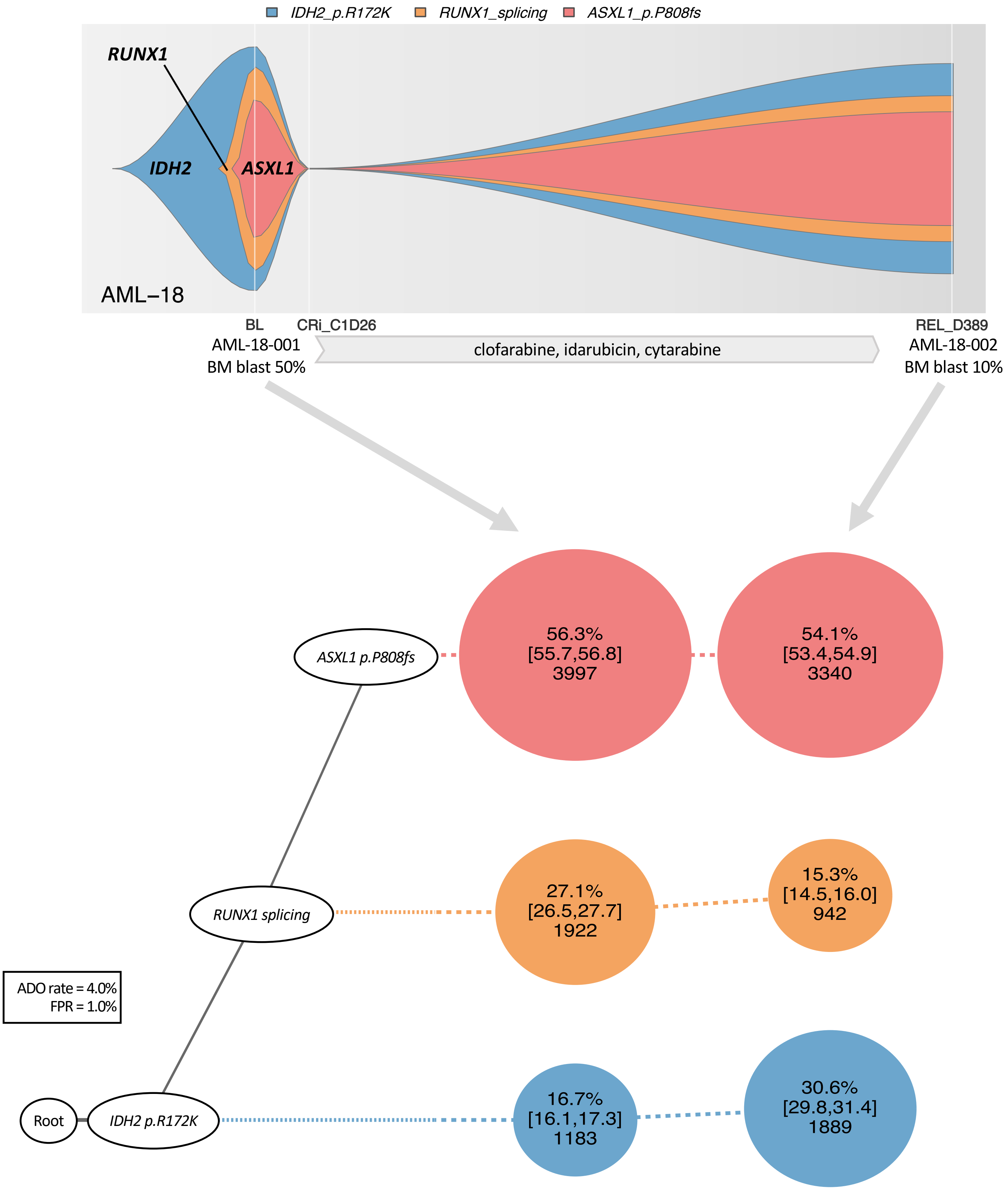

IDH2_p.R172K
RUNX1_splicing
ASXL1_p.P808fs
RUNX1
IDH2
ASXL1
AML−18
BL
AML-18-001
BM blast 50%
CRi_C1D26
clofarabine, idarubicin, cytarabine
REL_D389
AML-18-002
BM blast 10%
ASXL1 p.P808fs
56.3%
[55.7,56.8]
3997
54.1%
[53.4,54.9]
3340
RUNX1 splicing
27.1%
[26.5,27.7]
1922
15.3%
[14.5,16.0]
942
ADO rate = 4.0%
FPR = 1.0%
Root
IDH2 p.R172K
16.7%
[16.1,17.3]
1183
30.6%
[29.8,31.4]
1889

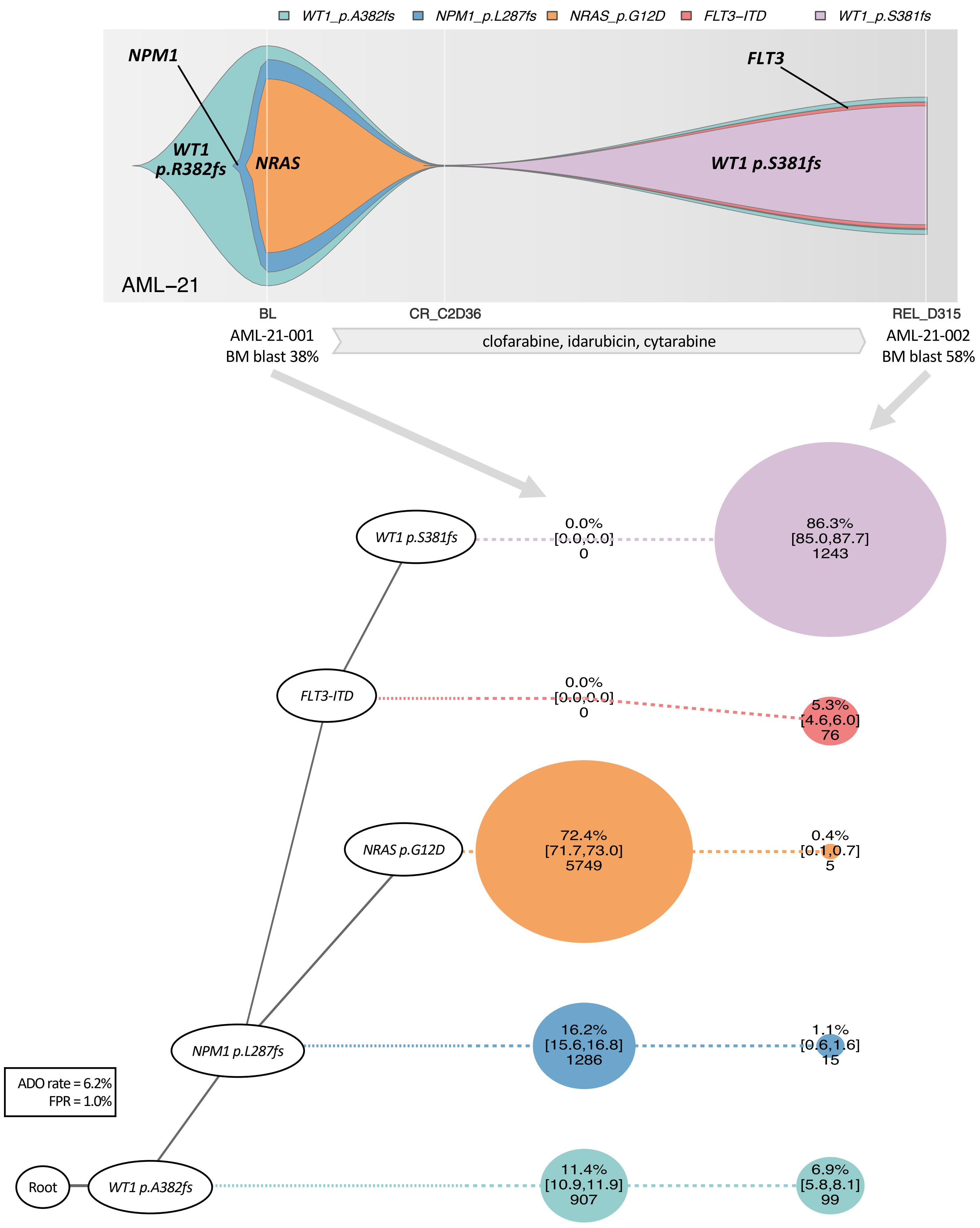

WT1_p.A382fs
NPM1_p.L287fs
NRAS_p.G12D
FLT3–ITD
WT1_p.S381fs
NPM1
WT1 p.R382fs
NRAS
FLT3
WT1 p.S381fs
AML–21
BL
AML-21-001
BM blast 38%
CR_C2D36
clofarabine, idarubicin, cytarabine
REL_D315
AML-21-002
BM blast 58%
WT1 p.S381fs
0.0%
[0.0,0.0]
0
86.3%
[85.0,87.7]
1243
FLT3-ITD
0.0%
[0.0,0.0]
0
5.3%
[4.6,6.0]
76
NRAS p.G12D
72.4%
[71.7,73.0]
5749
0.4%
[0.1,0.7]
5
NPM1 p.L287fs
16.2%
[15.6,16.8]
1286
1.1%
[0.6,1.6]
15
ADO rate = 6.2%
FPR = 1.0%
Root
WT1 p.A382fs
11.4%
[10.9,11.9]
907
6.9%
[5.8,8.1]
99

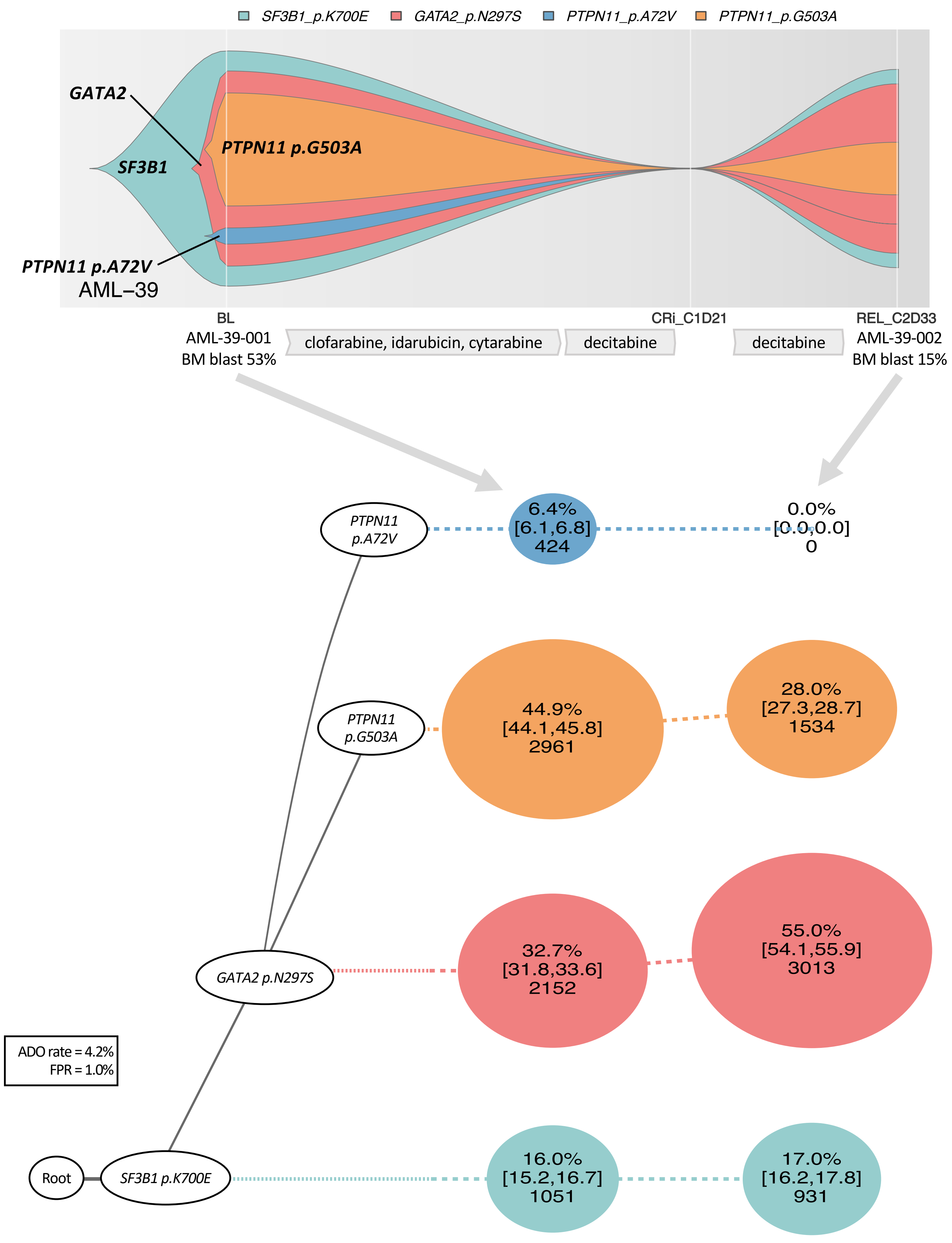
SF3B1_p.K700E
GATA2_p.N297S
PTPN11_p.A72V
PTPN11_p.G503A
GATA2
SF3B1
PTPN11 p.G503A
PTPN11 p.A72V
AML–39
BL
AML-39-001
BM blast 53%
clofarabine, idarubicin, cytarabine
decitabine
CRi_C1D21
decitabine
REL_C2D33
AML-39-002
BM blast 15%
PTPN11 p.A72V
6.4% [6.1,6.8] 424
0.0% [0.0,0.0] 0
PTPN11 p.G503A
44.9% [44.1,45.8] 2961
28.0% [27.3,28.7] 1534
GATA2 p.N297S
32.7% [31.8,33.6] 2152
55.0% [54.1,55.9] 3013
ADO rate = 4.2%
FPR = 1.0%
Root
SF3B1 p.K700E
16.0% [15.2,16.7] 1051
17.0% [16.2,17.8] 931

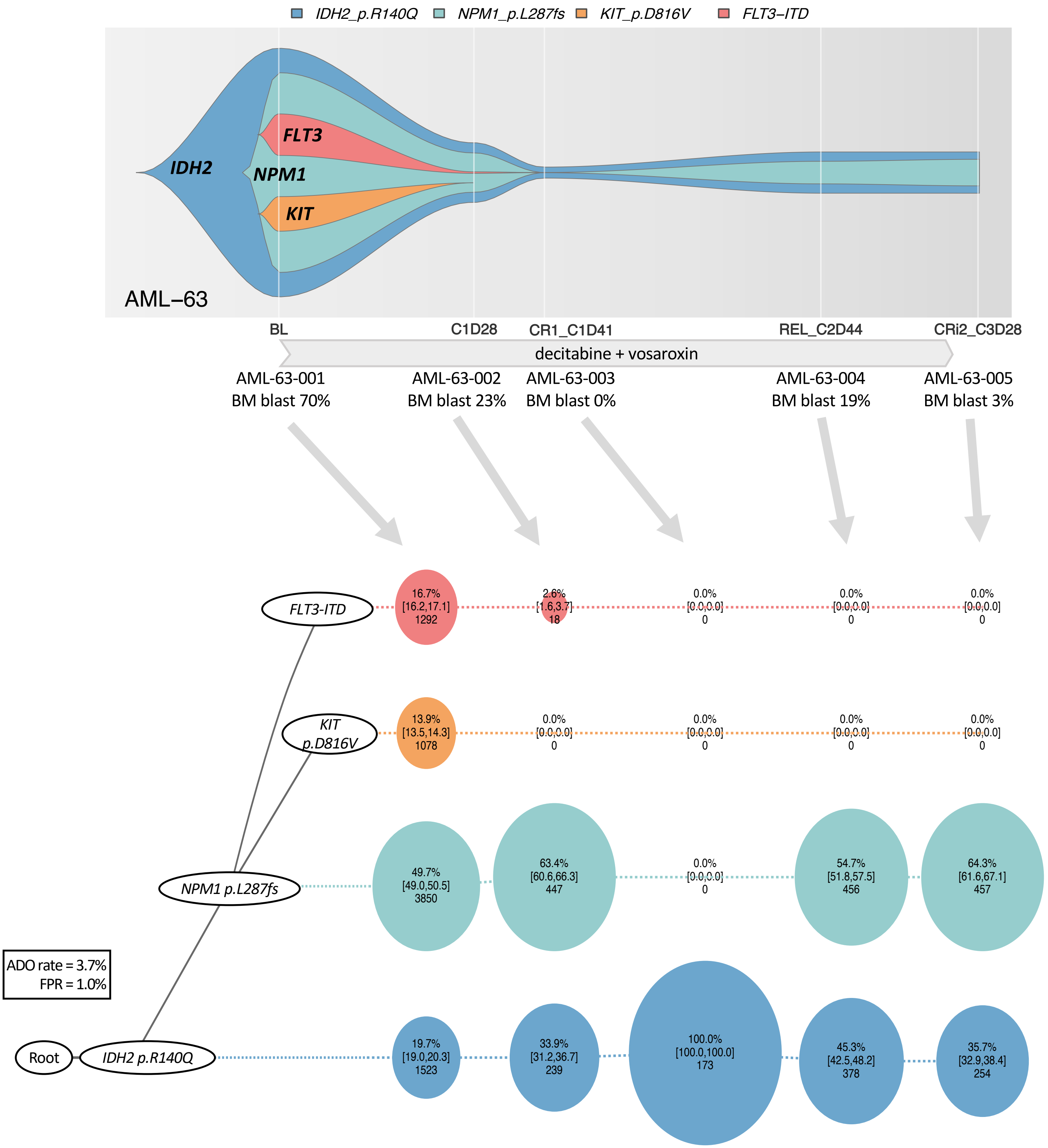

IDH2_p.R140Q
NPM1_p.L287fs
KIT_p.D816V
FLT3–ITD
IDH2
NPM1
FLT3
KIT
AML–63
BL
C1D28
CR1_C1D41
REL_C2D44
CRi2_C3D28
decitabine + vosaroxin
AML-63-001
BM blast 70%
AML-63-002
BM blast 23%
AML-63-003
BM blast 0%
AML-63-004
BM blast 19%
AML-63-005
BM blast 3%
FLT3-ITD
16.7% [16.2,17.1] 1292
2.6% [1.6,3.7] 18
0.0% [0.0,0.0] 0
0.0% [0.0,0.0] 0
0.0% [0.0,0.0] 0
KIT p.D816V
13.9% [13.5,14.3] 1078
0.0% [0.0,0.0] 0
0.0% [0.0,0.0] 0
0.0% [0.0,0.0] 0
0.0% [0.0,0.0] 0
NPM1 p.L287fs
49.7% [49.0,50.5] 3850
63.4% [60.6,66.3] 447
0.0% [0.0,0.0] 0
54.7% [51.8,57.5] 456
64.3% [61.6,67.1] 457
ADO rate = 3.7%
FPR = 1.0%
Root
IDH2 p.R140Q
19.7% [19.0,20.3] 1523
33.9% [31.2,36.7] 239
100.0% [100.0,100.0] 173
45.3% [42.5,48.2] 378
35.7% [32.9,38.4] 254

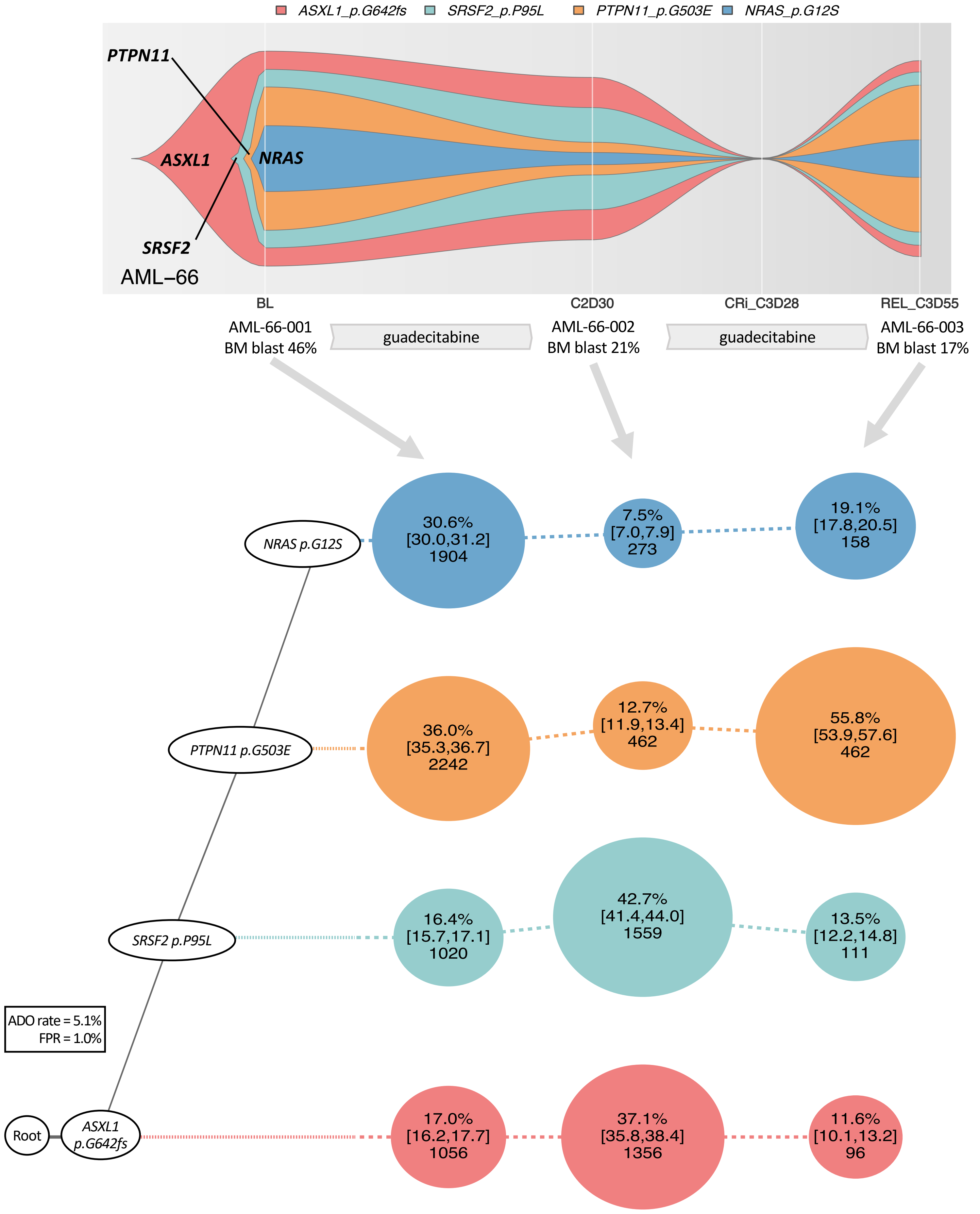
ASXL1_p.G642fs
SRSF2_p.P95L
PTPN11_p.G503E
NRAS_p.G12S
PTPN11
ASXL1
NRAS
SRSF2
AML−66
BL
C2D30
CRi_C3D28
REL_C3D55
AML-66-001
BM blast 46%
guadecitabine
AML-66-002
BM blast 21%
guadecitabine
AML-66-003
BM blast 17%
NRAS p.G12S
30.6%
[30.0,31.2]
1904
7.5%
[7.0,7.9]
273
19.1%
[17.8,20.5]
158
PTPN11 p.G503E
36.0%
[35.3,36.7]
2242
12.7%
[11.9,13.4]
462
55.8%
[53.9,57.6]
462
SRSF2 p.P95L
16.4%
[15.7,17.1]
1020
42.7%
[41.4,44.0]
1559
13.5%
[12.2,14.8]
111
ADO rate = 5.1%
FPR = 1.0%
Root
ASXL1 p.G642fs
17.0%
[16.2,17.7]
1056
37.1%
[35.8,38.4]
1356
11.6%
[10.1,13.2]
96

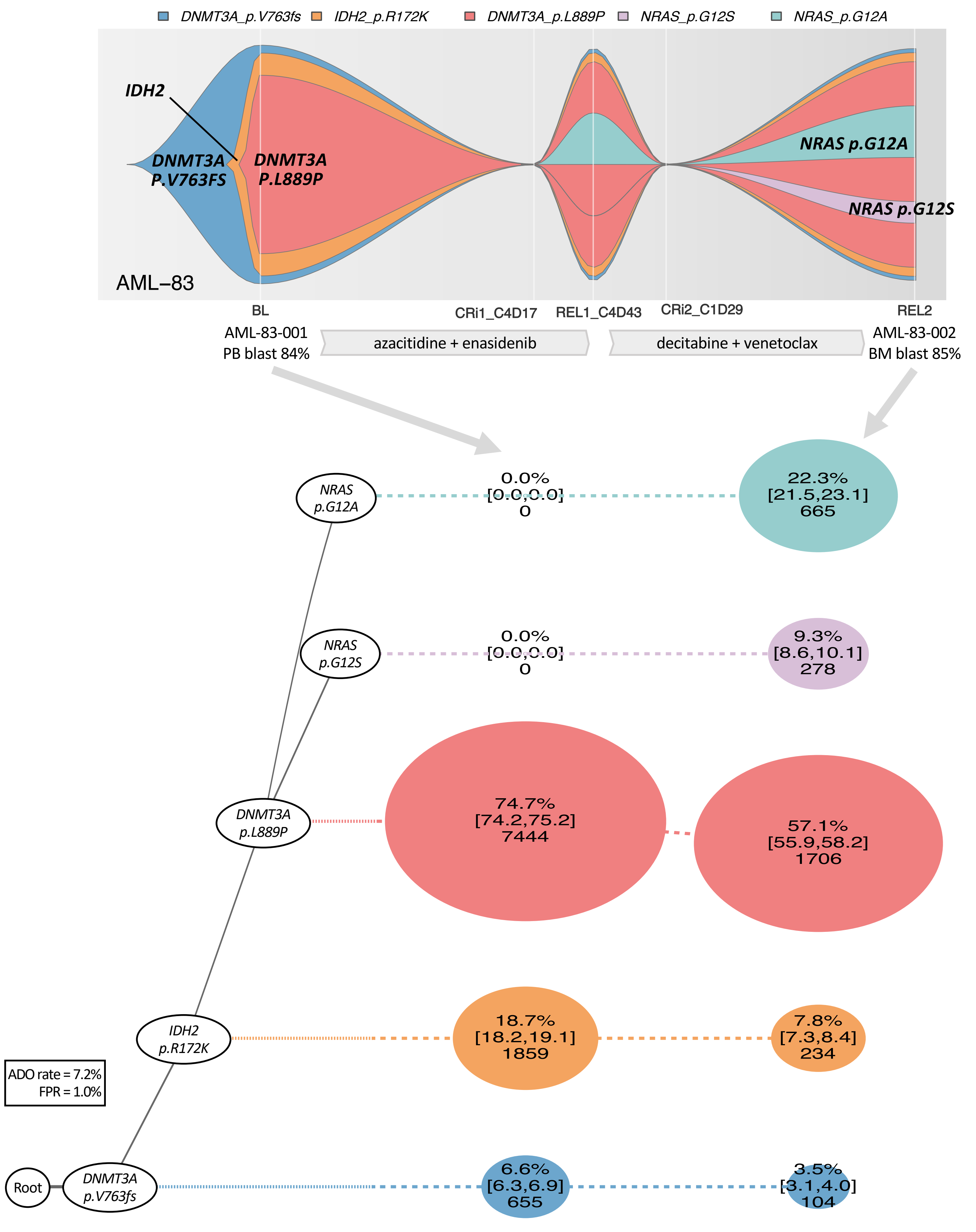

DNMT3A_p.V763fs
IDH2_p.R172K
DNMT3A_p.L889P
NRAS_p.G12S
NRAS_p.G12A
IDH2
DNMT3A P.V763FS
DNMT3A P.L889P
NRAS p.G12A
NRAS p.G12S
AML–83
BL
CRi1_C4D17
REL1_C4D43
CRi2_C1D29
REL2
AML-83-001
PB blast 84%
azacitidine + enasidenib
decitabine + venetoclax
AML-83-002
BM blast 85%
NRAS p.G12A
0.0% [0.0,0.0] 0
22.3% [21.5,23.1] 665
NRAS p.G12S
0.0% [0.0,0.0] 0
9.3% [8.6,10.1] 278
DNMT3A p.L889P
74.7% [74.2,75.2] 7444
57.1% [55.9,58.2] 1706
IDH2 p.R172K
18.7% [18.2,19.1] 1859
7.8% [7.3,8.4] 234
ADO rate = 7.2%
FPR = 1.0%
Root
DNMT3A p.V763fs
6.6% [6.3,6.9] 655
3.5% [3.1,4.0] 104

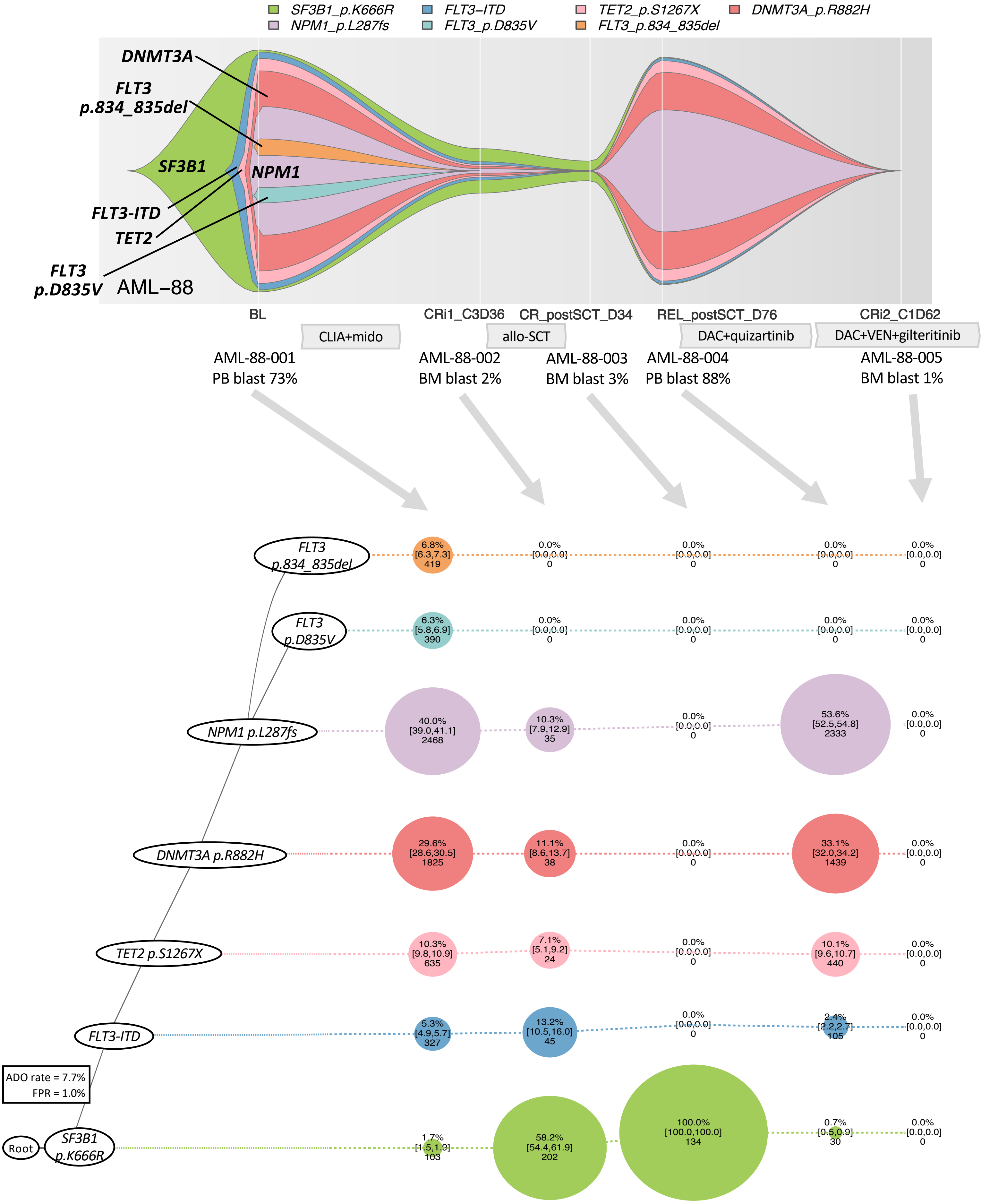
SF3B1_p.K666R
FLT3–ITD
TET2_p.S1267X
DNMT3A_p.R882H
NPM1_p.L287fs
FLT3_p.D835V
FLT3_p.834_835del
DNMT3A
FLT3 p.834_835del
SF3B1
NPM1
FLT3-ITD
TET2
FLT3 p.D835V
AML–88
BL
CRi1_C3D36
CR_postSCT_D34
REL_postSCT_D76
CRi2_C1D62
CLIA+mido
allo-SCT
DAC+quizartinib
DAC+VEN+gilteritinib
AML-88-001
PB blast 73%
AML-88-002
BM blast 2%
AML-88-003
BM blast 3%
AML-88-004
PB blast 88%
AML-88-005
BM blast 1%
FLT3 p.834_835del
6.8% [6.3,7.3] 419
0.0% [0.0,0.0] 0
0.0% [0.0,0.0] 0
0.0% [0.0,0.0] 0
0.0% [0.0,0.0] 0
FLT3 p.D835V
6.3% [5.8,6.9] 390
0.0% [0.0,0.0] 0
0.0% [0.0,0.0] 0
0.0% [0.0,0.0] 0
0.0% [0.0,0.0] 0
NPM1 p.L287fs
40.0% [39.0,41.1] 2468
10.3% [7.9,12.9] 35
0.0% [0.0,0.0] 0
53.6% [52.5,54.8] 2333
0.0% [0.0,0.0] 0
DNMT3A p.R882H
29.6% [28.6,30.5] 1825
11.1% [8.6,13.7] 38
0.0% [0.0,0.0] 0
33.1% [32.0,34.2] 1439
0.0% [0.0,0.0] 0
TET2 p.S1267X
10.3% [9.8,10.9] 635
7.1% [5.1,9.2] 24
0.0% [0.0,0.0] 0
10.1% [9.6,10.7] 440
0.0% [0.0,0.0] 0
FLT3-ITD
5.3% [4.9,5.7] 327
13.2% [10.5,16.0] 45
0.0% [0.0,0.0] 0
2.4% [2.2,2.7] 105
0.0% [0.0,0.0] 0
ADO rate = 7.7%
FPR = 1.0%
Root
SF3B1 p.K666R
1.7% [1.5,1.9] 103
58.2% [54.4,61.9] 202
100.0% [100.0,100.0] 134
0.7% [0.5,0.9] 30
0.0% [0.0,0.0] 0

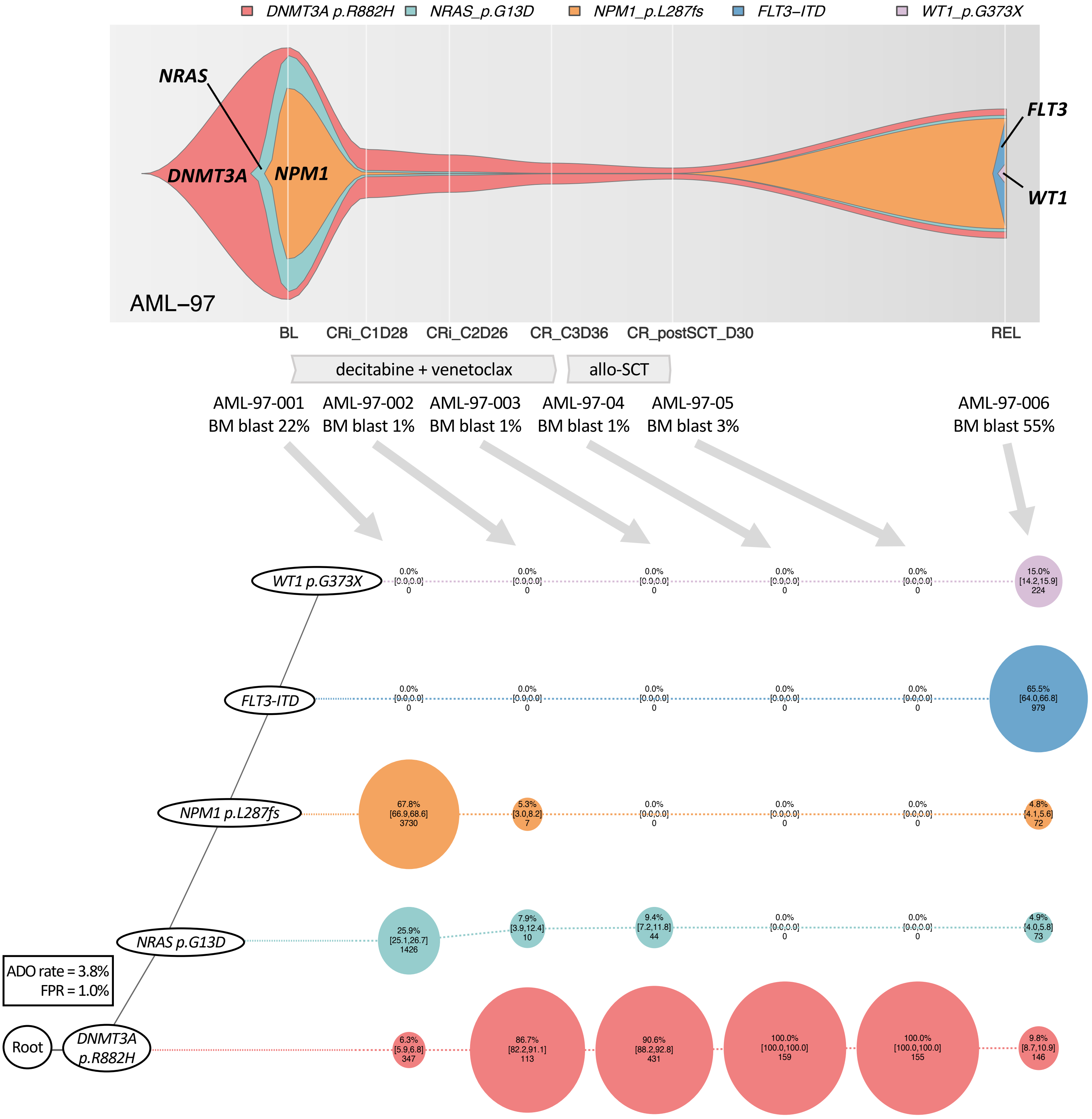

DNMT3A p.R882H
NRAS_p.G13D
NPM1_p.L287fs
FLT3–ITD
WT1_p.G373X
NRAS
DNMT3A
NPM1
FLT3
WT1
AML–97
BL
CRi_C1D28
CRi_C2D26
CR_C3D36
CR_postSCT_D30
REL
decitabine + venetoclax
allo-SCT
AML-97-001
BM blast 22%
AML-97-002
BM blast 1%
AML-97-003
BM blast 1%
AML-97-04
BM blast 1%
AML-97-05
BM blast 3%
AML-97-006
BM blast 55%
WT1 p.G373X
FLT3-ITD
NPM1 p.L287fs
NRAS p.G13D
Root
DNMT3A p.R882H
ADO rate = 3.8%
FPR = 1.0%
15.0% [14.2,15.9] 224
65.5% [64.0,66.8] 979
67.8% [66.9,68.6] 3730
5.3% [3.0,8.2] 7
4.8% [4.1,5.6] 72
25.9% [25.1,26.7] 1426
7.9% [3.9,12.4] 10
9.4% [7.2,11.8] 44
4.9% [4.0,5.8] 73
6.3% [5.9,6.8] 347
86.7% [82.2,91.1] 113
90.6% [88.2,92.8] 431
100.0% [100.0,100.0] 159
100.0% [100.0,100.0] 155
9.8% [8.7,10.9] 146

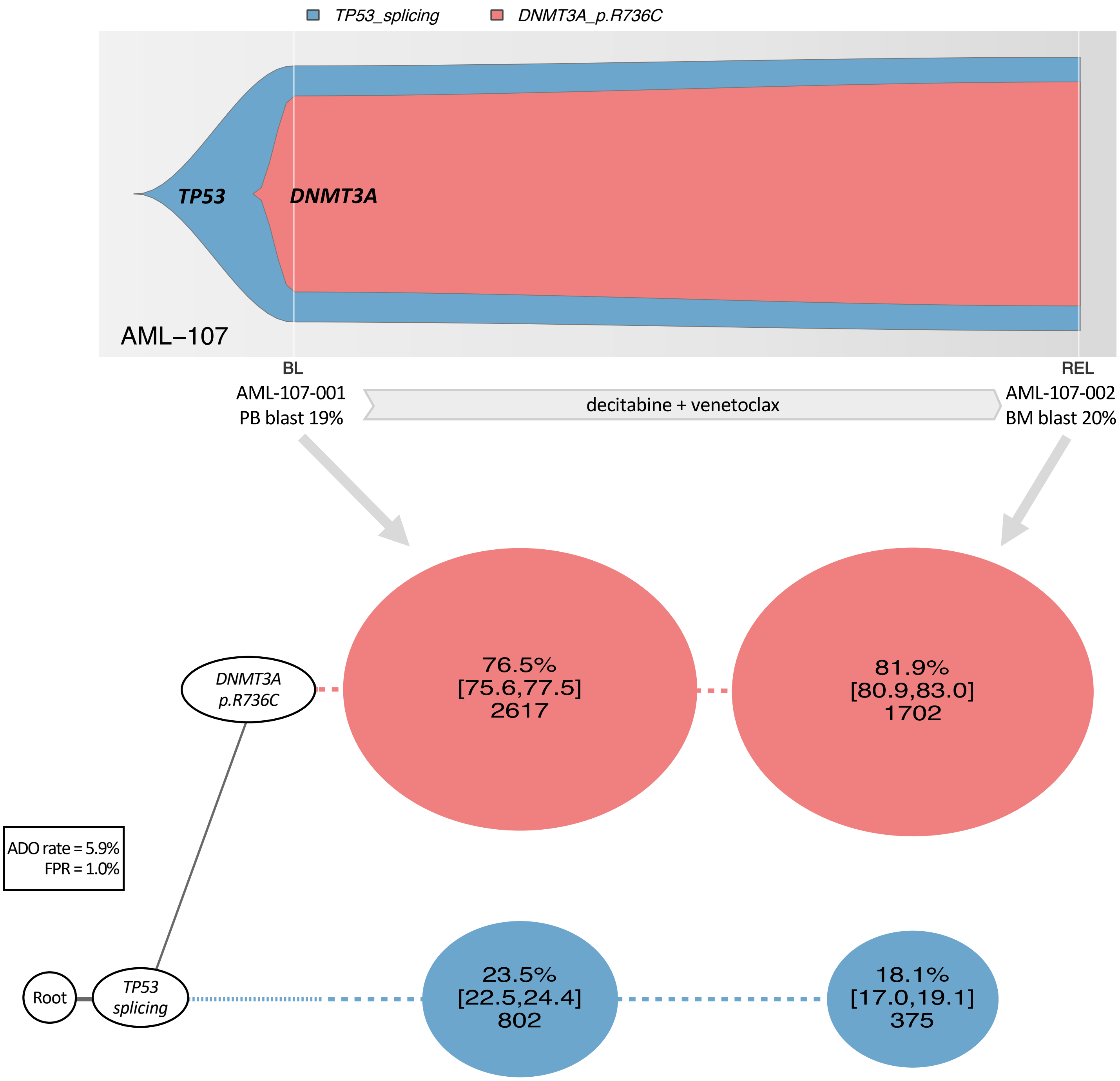
TP53_splicing
DNMT3A_p.R736C
TP53
DNMT3A
AML−107
BL
AML-107-001
PB blast 19%
decitabine + venetoclax
REL
AML-107-002
BM blast 20%
DNMT3A
p.R736C
76.5%
[75.6,77.5]
2617
81.9%
[80.9,83.0]
1702
ADO rate = 5.9%
FPR = 1.0%
Root
TP53
splicing
23.5%
[22.5,24.4]
802
18.1%
[17.0,19.1]
375

Supplementary Fig. 14. Dynamic clonal evolution patterns in AML during and after treatment. Detailed case description is available in Supplementary Methods.

Supplementary Fig. 15

AML-46-001

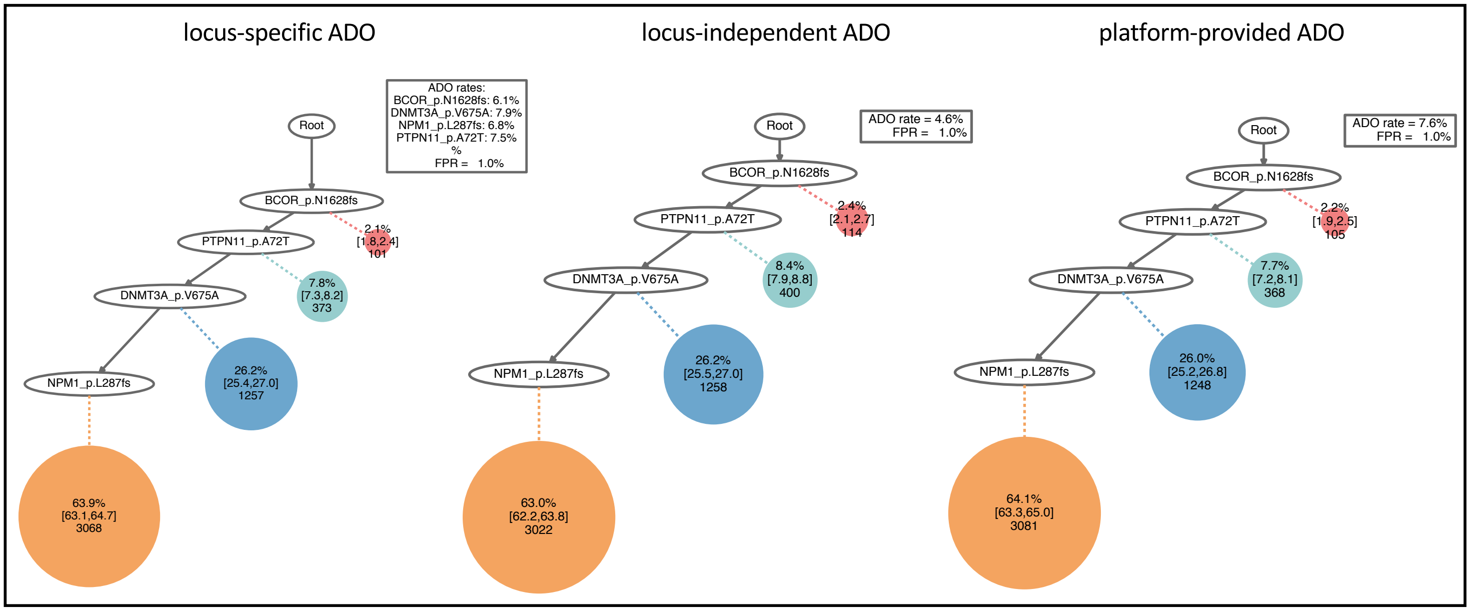

AML-55-001

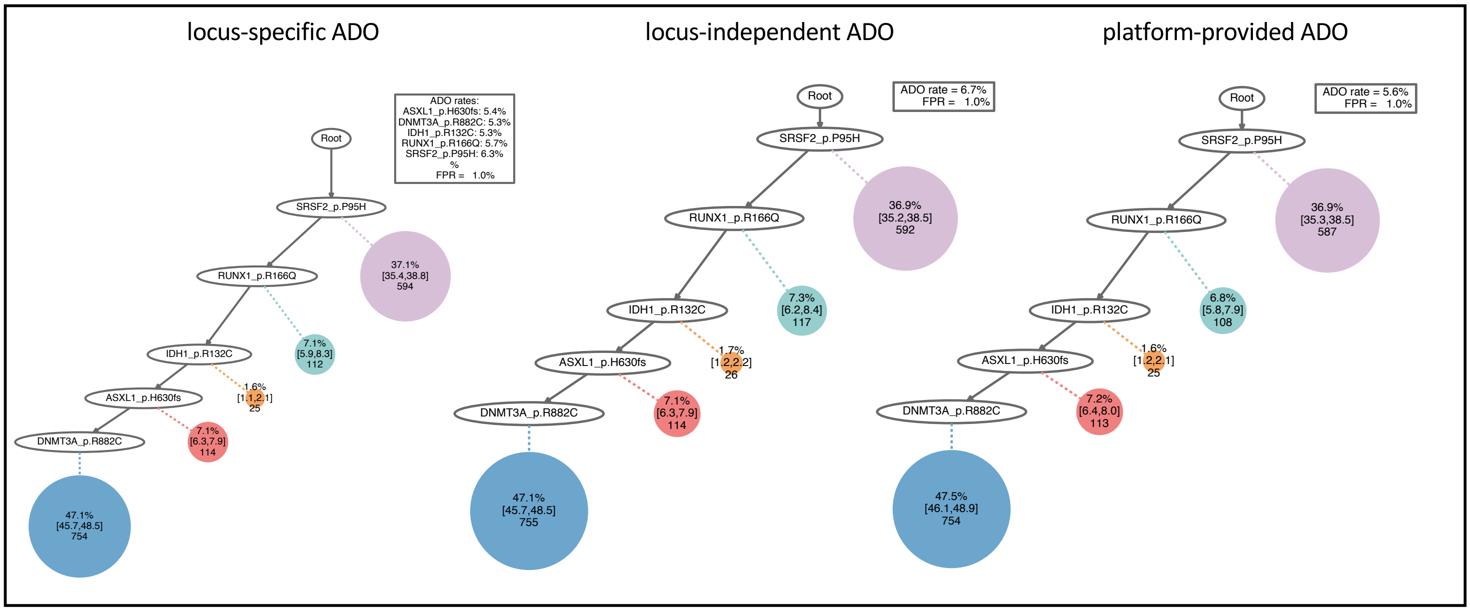

AML-51-001

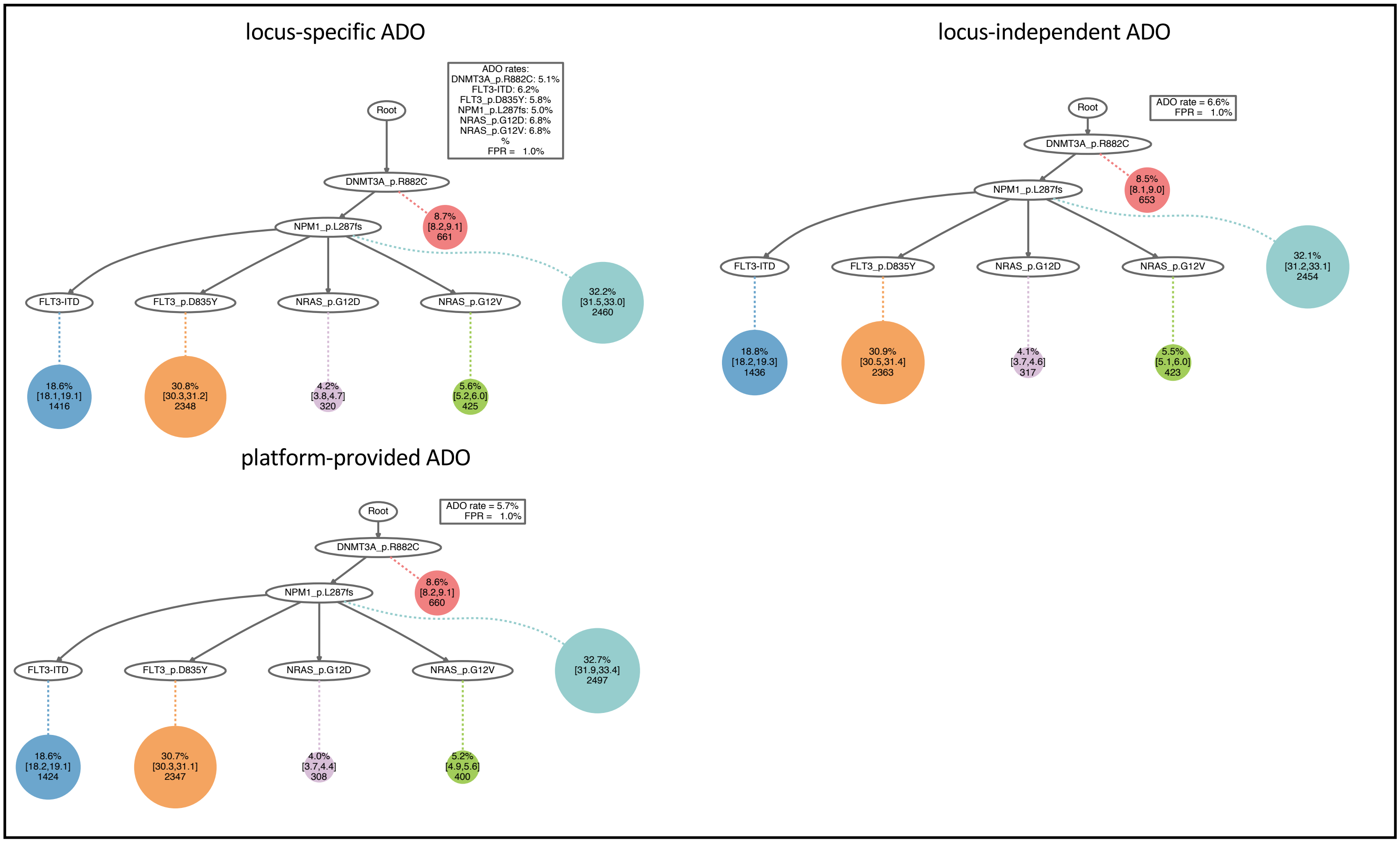

locus-specific ADO
ADO rates:
DNMT3A_p.R882C: 5.1%
FLT3-ITD: 6.2%
FLT3_p.D835Y: 5.8%
NPM1_p.L287fs: 5.0%
NRAS_p.G12D: 6.8%
NRAS_p.G12V: 6.8%
%
FPR = 1.0%
Root
DNMT3A_p.R882C
8.7% [8.2,9.1] 661
NPM1_p.L287fs
32.2% [31.5,33.0] 2460
FLT3-ITD
FLT3_p.D835Y
NRAS_p.G12D
NRAS_p.G12V
18.6% [18.1,19.1] 1416
30.8% [30.3,31.2] 2348
4.2% [3.8,4.7] 320
5.6% [5.2,6.0] 425
locus-independent ADO
ADO rate = 6.6%
FPR = 1.0%
Root
DNMT3A_p.R882C
8.5% [8.1,9.0] 653
NPM1_p.L287fs
32.1% [31.2,33.1] 2454
FLT3-ITD
FLT3_p.D835Y
NRAS_p.G12D
NRAS_p.G12V
18.8% [18.2,19.3] 1436
30.9% [30.5,31.4] 2363
4.1% [3.7,4.6] 317
5.5% [5.1,6.0] 423
platform-provided ADO
ADO rate = 5.7%
FPR = 1.0%
Root
DNMT3A_p.R882C
8.6% [8.2,9.1] 660
NPM1_p.L287fs
32.7% [31.9,33.4] 2497
FLT3-ITD
FLT3_p.D835Y
NRAS_p.G12D
NRAS_p.G12V
18.6% [18.2,19.1] 1424
30.7% [30.3,31.1] 2347
4.0% [3.7,4.4] 308
5.2% [4.9,5.6] 400

Supplementary Fig. 15. Reconstruction of mutation phylogeny using locus-specific allele dropout (ADO) rate. Phylogeny trees based on all sequenced cells using locus-specific ADO rate (left), locus-independent ADO rate (middle), and platform-provided ADO rate (right, same as model 2 phylogeny trees), are compared in representative cases.

Supplementary References

1 Martin, M. Cutadapt removes adapter sequences from high-throughput sequencing reads. EBMnet J **17**, 3 (2011).

2 Bolger, A. M., Lohse, M. & Usadel, B. Trimmomatic: a flexible trimmer for Illumina sequence data. *Bioinformatics* **30**, 2114-2120 (2014).

3 Langmead, B., Trapnell, C., Pop, M. & Salzberg, S. L. Ultrafast and memory-efficient alignment of short DNA sequences to the human genome. *Genome Biol* **10**, R25 (2009).

4 Kim, D. *et al.* TopHat2: accurate alignment of transcriptomes in the presence of insertions, deletions and gene fusions. *Genome Biol* **14**, R36 (2013).

5 McKenna, A. *et al.* The Genome Analysis Toolkit: a MapReduce framework for analyzing next-generation DNA sequencing data. *Genome Res* **20**, 1297-1303 (2010).

6 DePristo, M. A. *et al.* A framework for variation discovery and genotyping using next-generation DNA sequencing data. *Nature genetics* **43**, 491-498 (2011).

7 Van der Auwera, G. A. *et al.* From FastQ data to high confidence variant calls: the Genome Analysis Toolkit best practices pipeline. *Curr Protoc Bioinformatics* **43**, 11 10 11-33 (2013).

8 Tan, A., Abecasis, G. R. & Kang, H. M. Unified representation of genetic variants. *Bioinformatics* **31**, 2202-2204 (2015).

9 Zeisel, A. *et al.* Molecular Architecture of the Mouse Nervous System. *Cell* **174**, 999-1014 e1022 (2018).

10 Zhang, J. *et al.* Intratumor heterogeneity in localized lung adenocarcinomas delineated by multiregion sequencing. *Science* **346**, 256-259 (2014).

11 Li, H. *et al.* The Sequence Alignment/Map format and SAMtools. *Bioinformatics* **25**, 2078-2079 (2009).

12 Cibulskis, K. *et al.* Sensitive detection of somatic point mutations in impure and heterogeneous cancer samples. *Nature biotechnology* **31**, 213-219 (2013).

13 Ye, K., Schulz, M. H., Long, Q., Apweiler, R. & Ning, Z. Pindel: a pattern growth approach to detect break points of large deletions and medium sized insertions from paired-end short reads. *Bioinformatics* **25**, 2865-2871 (2009).

14 Papaemmanuil, E. *et al.* Clinical and biological implications of driver mutations in myelodysplastic syndromes. *Blood* **122**, 3616-3627; quiz 3699 (2013).
