# Supplementary material for: Clonal evolution of acute myeloid leukemia revealed by high-throughput single-cell genomics
Source: Nat Commun. 2020 Oct 21;11:5327. doi: 10.1038/s41467-020-19119-8 (PMC7577981; doi:10.1038/s41467-020-19119-8)
Supplement: Supplementary file 3 — Description of Additional Supplementary Files [file 41467_2020_19119_MOESM3_ESM.pdf]

Description of Additional Supplementary Files

File Name: Supplementary Data 1
Description: (Driver mutations detected by single-cell DNA sequencing)

File Name: Supplementary Data 2
Description: (List of 279 amplicons covered by the custom-designed panel)
