# Supplementary material for: Clonal evolution of acute myeloid leukemia revealed by high-throughput single-cell genomics
Source: Nat Commun. 2020 Oct 21;11:5327. doi: 10.1038/s41467-020-19119-8 (PMC7577981; doi:10.1038/s41467-020-19119-8)
Supplement: Supplementary file 6 — Reporting Summary [file 41467_2020_19119_MOESM6_ESM.pdf]

nature research

Corresponding author(s): Koichi Takahashi

Last updated by author(s): Aug 17, 2020

Reporting Summary

Nature Research wishes to improve the reproducibility of the work that we publish. This form provides structure for consistency and transparency in reporting. For further information on Nature Research policies, see Authors & Referees and the Editorial Policy Checklist .

Statistics

For all statistical analyses, confirm that the following items are present in the figure legend, table legend, main text, or Methods section.

n/a	Confirmed	
☐	☒	The exact sample size (*n*) for each experimental group/condition, given as a discrete number and unit of measurement
☐	☒	A statement on whether measurements were taken from distinct samples or whether the same sample was measured repeatedly
☐	☒	The statistical test(s) used AND whether they are one- or two-sided<br>*Only common tests should be described solely by name; describe more complex techniques in the Methods section.*
☒	☐	A description of all covariates tested
☐	☒	A description of any assumptions or corrections, such as tests of normality and adjustment for multiple comparisons
☐	☒	A full description of the statistical parameters including central tendency (e.g. means) or other basic estimates (e.g. regression coefficient) AND variation (e.g. standard deviation) or associated estimates of uncertainty (e.g. confidence intervals)
☐	☒	For null hypothesis testing, the test statistic (e.g. *F*, *t*, *r*) with confidence intervals, effect sizes, degrees of freedom and *P* value noted<br>*Give P values as exact values whenever suitable.*
☐	☒	For Bayesian analysis, information on the choice of priors and Markov chain Monte Carlo settings
☐	☒	For hierarchical and complex designs, identification of the appropriate level for tests and full reporting of outcomes
☐	☒	Estimates of effect sizes (e.g. Cohen's *d*, Pearson's *r*), indicating how they were calculated

Our web collection on statistics for biologists contains articles on many of the points above.

Software and code

Policy information about availability of computer code

Data collection	No codes were used for data collection.
Data analysis	R (ver. 3.4.3); GATK version 3.1; Tapestri Pipeline version 1.6.1; mutect version 1.1.4; pindel version 0.2.4; EZR version 1.4.1, ANNOVAR revision ID: 9f9e0f9efe83690a15a6aeb7714f1fc3a2341deb Date: 2018-04-16; ASCAT version 2.5.2; TrAp GUI version 0.3a; GenomeStudio v2.0, Quanta-Soft Analysis Pro software v1.0.596.

For manuscripts utilizing custom algorithms or software that are central to the research but not yet described in published literature, software must be made available to editors/reviewers. We strongly encourage code deposition in a community repository (e.g. GitHub). See the Nature Research guidelines for submitting code & software for further information.

Data

Policy information about availability of data

All manuscripts must include a data availability statement. This statement should provide the following information, where applicable:

- Accession codes, unique identifiers, or web links for publicly available datasets
- A list of figures that have associated raw data
- A description of any restrictions on data availability

Deidentified clinical and genetic data is available in supplementary information.

Field-specific reporting

Please select the one below that is the best fit for your research. If you are not sure, read the appropriate sections before making your selection.

Life sciences study design

All studies must disclose on these points even when the disclosure is negative.

Sample size	No sample size calculation was performed. We tried to analyze as many samples as possible for this study.
Data exclusions	No data exclusion was performed unless specified in the manuscript.
Replication	No replication was done, as this is human sample research. Rigor of the study was maintained by orthogonal validation of mutations.
Randomization	No randomization was done, since this study was mostly supervised without any intervention.
Blinding	No blinding was done. Blinding was no relevant as the data analysis for this study is mostly supervised.

Reporting for specific materials, systems and methods

We require information from authors about some types of materials, experimental systems and methods used in many studies. Here, indicate whether each material, system or method listed is relevant to your study. If you are not sure if a list item applies to your research, read the appropriate section before selecting a response.

Materials & experimental systems

n/a	Involved in the study	
☐	☒	Antibodies
☒	☐	Eukaryotic cell lines
☒	☐	Palaeontology
☐	☒	Animals and other organisms
☐	☒	Human research participants
☒	☐	Clinical data

Methods

n/a	Involved in the study	
☒	☐	ChIP-seq
☐	☒	Flow cytometry
☒	☐	MRI-based neuroimaging

Antibodies

Antibodies used

PDX analysis:
APC anti-human CD45 Antibody, Cat # 304012, clone HI30, Biolegend.

single-cell DNA/protein analysis:
Oligonucleotide-conjugated antibodies (CD13, CD33, CD3, CD19, CD22, CD11b, CD14, CD64, CD34, HLA-DR, CD117, CD123, CD38, CD90, CD45), Cat# MB04-0012, Mission Bio.
The antibodies that were sourced to build the above oligonucleotide-conjugated antibodies:
Anti-human CD13 Antibody, Cat# 301708, clone WM15, Biolegend
Anti-human CD33 Antibody, Cat# 303410, clone WM53, Biolegend
Anti-human CD3 Antibody, Cat# 300314, clone HIT3a, Biolegend
Anti-human CD19 Antibody, Cat# 302214, clone HIB19, Biolegend
Anti CD22 Antibody, Cat# ab213038, clone MYG13, abcam
Anti-mouse/human CD11b Antibody, Cat# 101214, clone M1/70, Biolegend
Anti-human CD14 Antibody, Cat# 301810, clone M5E2, Biolegend
Anti-human CD64 Antibody, Cat# 305016, clone 10.1, Biolegend
Anti-human CD34 Antibody, Cat# 130-108-040, clone AC136, Miltenyi
Anti-human HLA-DR Antibody, Cat# 307648, clone L243, Biolegend
Anti-human CD117 Antibody, Cat# 323404, clone A3C6E2, Biolegend
Anti-human CD123 Antibody, Cat# 130-108-026, clone AC145, Miltenyi
Anti-human CD38 Antibody, Cat# 130-122-307, clone REA572, Miltenyi
Anti CD90 Antibody, Cat# ab212885, clone AF-9, abcam
Anti-human CD45 Antibody, Cat# 130-108-020, clone 5B1, Miltenyi

Validation

Antibodies were validated by the manufacturer who provided references on their websites using the catalog number provided above:
Biolegend - https://www.biolegend.com/
Abcam - http://www.abcam.com/products
Miltenyi biotec - https://www.miltenyibiotec.com/US-en/

Animals and other organisms

Policy information about studies involving animals; ARRIVE guidelines recommended for reporting animal research

Laboratory animals	NOD.Cg-PrkdcscidIl2rgtm1WjlTg(CMV-IL3,CSF2,KITLG)1Eav/MloySzJ female mice (NSG-SGM3; 8–12 weeks of age; JAX 013062; The Jackson Laboratory) were used for xenotransplantation. Mice were housed in AAALAC-accredited, specific-pathogen-free animal care facilities at Baylor College of Medicine (BCM). Room temperatures were maintained around 70 degrees Fahrenheit plus or minus 2 degrees. Humidity for animal holding rooms ranged from 30 to 70%. The standard light timer was set on a 14-hour light cycle with the lights coming on at 6 am and off at 8 pm. All procedures were approved by BCM Institutional Animal Care and Use Committees.
Wild animals	The study did not involve wild animals.
Field-collected samples	The study did not involve samples collected from the field.
Ethics oversight	Baylor College of Medicine

Note that full information on the approval of the study protocol must also be provided in the manuscript.

Human research participants

Policy information about studies involving human research participants

Population characteristics	The study population consisted of adult patients (age >20) with newly-diagnosed or relapsed/refractory acute myeloid leukemia. 48 (39%) patients were female, and 75 (61%) patients were male. 49 (40%) patients had NPM1 mutations, 47 (38%) with FLT3, 45 (37%) with DNMT3A, 45 (37%) with NRAS, 33 (27%) with IDH2, 25 (20%) with RUNX1, 25 (20%) with SRSF2, 20 (16%) with TET2, 19 (15%) with KRAS mutations. 88 (72%) patients were previously untreated, and 35 (28%) patients had relapsed/refractory diseases. 49 (40%) patients were treated with idarubicine/cytarabine-based chemotherapy, 12 (10%) with cytarabine-based chemotherapy, 27 (22%) with decitabine and venetoclax, 24 (20%) with hypomethylating agents without venetoclax, 11 (9%) with other drugs.
Recruitment	We studied patients with AML who received therapy in our institution and gave consent to sample/data collection. We tried to capture as many samples as possible without self-selection bias. This is a descriptive study of the single-cell genomic landscape of AML, and the potential selection bias are unlikely to impact the results.
Ethics oversight	The University of Texas MD Anderson Cancer Center

Note that full information on the approval of the study protocol must also be provided in the manuscript.

Flow Cytometry

Plots

Confirm that:

- [x] The axis labels state the marker and fluorochrome used (e.g. CD4-FITC).
- [x] The axis scales are clearly visible. Include numbers along axes only for bottom left plot of group (a 'group' is an analysis of identical markers).
- [x] All plots are contour plots with outliers or pseudocolor plots.
- [x] A numerical value for number of cells or percentage (with statistics) is provided.

Methodology

Sample preparation	human:<br>Bone marrow aspirate samples were used.<br>PDX:<br>Bone marrow cells from tibias, femurs, pelvic bones, and vertebrae were used.
Instrument	human:<br>Flow cytometry was performed on FACSCanto II instruments (BD Biosciences, San Diego, CA).<br>PDX:<br>Flow cytometry was performed on FACSAria II (BD Biosciences).
Software	human:<br>Data analysis was performed using FCS Express 6 (De Novo Software, Pasadena, CA).<br>PDX:<br>Data analysis was performed using FlowJo X 10.0.

Cell population abundance	human:<br>Not applicable since the human clinical flow cytometry was bulk analysis, and the samples were not sorted.<br>PDX:<br>Purity of sorted samples were not checked. Since the sequencing data was mapped to human reference genome, potential residual mice cells had minimal affect on data interpretation.
Gating strategy	human:<br>Patients' bone marrow cells were gated on live cells based on FSC-A and SSC-A, then<br>gated on singlets based upon FSC-A and SSC-H, then gated on nucleated cells based on CD45, then leukemia blasts based on CD45 dim expression.<br><br>PDX:<br>FSC and SSC plot was created to ensure all the expected populations were visible and the debris and laser noise were removed. Cells were then gated on singlets based upon FSC-H and FSC-A. Viable patients' derived cells (DAPI-hCD45+) were then sorted based on SSC-A and CD45 for the analysis of engrafted genotype. An unstained control and biological comparison controls (un-transplanted) was used to determine the level of background fluorescence or autofluorescence and set voltages and negative/positive gate.

☒ Tick this box to confirm that a figure exemplifying the gating strategy is provided in the Supplementary Information.
