# Supplementary material for: Clonal evolution of acute myeloid leukemia revealed by high-throughput single-cell genomics
Source: Nat Commun. 2020 Oct 21;11:5327. doi: 10.1038/s41467-020-19119-8 (PMC7577981; doi:10.1038/s41467-020-19119-8)
Supplement: Supplementary file 2 — Peer Review File [file 41467_2020_19119_MOESM2_ESM.pdf]

Editorial Note: This manuscript has been previously reviewed at another journal that is not operating a transparent peer review scheme. This document only contains reviewer comments and rebuttal letters for versions considered at Nature Communications.
